# Cryptic Distant Relatives Are Common in Both Isolated and Cosmopolitan Genetic Samples

**DOI:** 10.1371/journal.pone.0034267

**Published:** 2012-04-03

**Authors:** Brenna M. Henn, Lawrence Hon, J. Michael Macpherson, Nick Eriksson, Serge Saxonov, Itsik Pe'er, Joanna L. Mountain

**Affiliations:** 1 23andMe, Inc., Mountain View, California, United States of America; 2 Department of Genetics, Stanford University, Stanford, California, United States of America; 3 Department of Computer Science, Columbia University, New York, New York, United States of America; University of Utah, United States of America

## Abstract

Although a few hundred single nucleotide polymorphisms (SNPs) suffice to infer close familial relationships, high density genome-wide SNP data make possible the inference of more distant relationships such as 2^nd^ to 9^th^ cousinships. In order to characterize the relationship between genetic similarity and degree of kinship given a timeframe of 100–300 years, we analyzed the sharing of DNA inferred to be identical by descent (IBD) in a subset of individuals from the 23andMe customer database (n = 22,757) and from the Human Genome Diversity Panel (HGDP-CEPH, n = 952). With data from 121 populations, we show that the average amount of DNA shared IBD in most ethnolinguistically-defined populations, for example Native American groups, Finns and Ashkenazi Jews, differs from continentally-defined populations by several orders of magnitude. Via extensive pedigree-based simulations, we determined bounds for predicted degrees of relationship given the amount of genomic IBD sharing in both endogamous and ‘unrelated’ population samples. Using these bounds as a guide, we detected tens of thousands of 2^nd^ to 9^th^ degree cousin pairs within a heterogenous set of 5,000 Europeans. The ubiquity of distant relatives, detected via IBD segments, in both ethnolinguistic populations and in large ‘unrelated’ populations samples has important implications for genetic genealogy, forensics and genotype/phenotype mapping studies.

## Introduction

The emergence of genotyping platforms that assay hundreds of thousands of sites across the genome has facilitated the rapid growth of databases with genome-wide data for human populations [1]–[3]. These large databases may include data for pairs of individuals who are cryptically related over the last 10 generations, e.g., on the order of 2^nd^ to 9^th^ cousins. Such cousins often share extended segments of DNA that are identical except for recent mutations. Genetic similarity metrics, such as the length of DNA segments that are consistent with identity by descent (IBD) from a common ancestor, can be used to detect relatively distantly related individuals [1], [4]. Because recombination breaks down these shared segments rapidly, pairs of relatives vary in the number and length of shared segments that they inherit from a common ancestor. Fourth cousins, for example, may or may not share any long segments that are identical by descent [5].

For a given pair of individuals, the inferred IBD pattern depends on a broad set of factors, including not only the rate and pattern of recombination, but also the accuracy of the IBD detection algorithm and the type of genetic data (e.g., autosomal SNPs) under consideration. The pattern of IBD also depends on the number of ancestors that the two individuals have in common and the number of generations since each of these common ancestors. These factors, in turn, depend on the structure of the relevant population. In a finite, panmictic population, for a random pair of individuals from the same generation, the expected degree of cousinship, that is, the *n*+1 number of generations back to the most recent common ancestor, is a function of the effective population size, N_e_ [6], [7]. For some human populations, the average pair of individuals is related as closely as 2^nd^–4^th^ cousins, and in fact, may be related through multiple shared, recent ancestors. [8], [9]. However, for many large, panmictic human populations a pair of individuals chosen at random is unlikely to be closer than 10^th^–20^th^ cousins [10].

Detecting these IBD patterns and predicting relationships on the basis of these patterns can be complicated by endogamy, the tendency to choose a mate from the same ethnic group or geographic location, that is associated with reduced effective population size. In smaller endogamous populations, any two individuals are likely to be genetically similar because they share either one or *multiple* recent common ancestors. The level of homozygosity within endogamous populations is therefore higher than in large populations and those receiving gene flow from neighboring groups [11]. Malecot [12] introduced the concept of an inbreeding coefficient, the probability of inheriting identical alleles from both parents. The inbreeding coefficient of an individual is equivalent to the coancestry, or kinship, coefficient of that individual's parents. The higher the kinship coefficient of a pair of individuals, the more likely they are to share DNA that is IBD. Inferring that DNA segments from two individuals are IBD is conceptually similar to another widely used metric of genetic similarity, runs of homozygosity. Runs of homozygosity within an individual indicate that the homologous segments trace back to a recent common ancestor.

Many genomic studies of population history have focused on analyses of genetic diversity that reflect ancient demographic events [13], [14]. However, endogamy in human populations likely reflects demographic processes occurring only on the order of hundreds, rather than thousands of years [15]–[17]. Patterns of IBD within populations provide the opportunity to examine the relationship between genetic similarity and kinship based on common ancestry between 100 and 300 years ago. In turn, the number and sizes of segments shared IBD provide a basis for estimating the kinship relationship between any two individuals.

Mountain and Ramakrishnan [18] explored the difference between ethnolinguistically- and continentally-defined populations in their distributions of genomic similarity between individuals using short tandem repeats (STRs) analyzed in the Human Genome Diversity Panel populations (HGDP-CEPH). In this study we characterize the levels of kinship within ethnolinguistically and more broadly defined populations using extensive, genome-wide single nucleotide polymorphism (SNP) data. Specifically, we infer identity by descent from unphased autosomal SNP data in order to explore the distribution of IBD segments in 121 populations from the HGDP-CEPH (n = 952) and a 23andMe dataset (n = 22,757), a subset of genomic data from the 23andMe customer database. The latter set includes data from Ashkenazi Jewish and broadly-defined “European” and “Asian” populations. In order to interpret the patterns of population-level sharing, we simulate extended pedigrees for different ethnic groups and estimated the expected extent of sharing for 1^st^ through 9^th^ cousins. Based on these simulations, we infer the likely relationship between each pair individuals within each population sample. This approach is an alternative to methods that use allele frequencies and identity by state (IBS) to infer relationships from low-density genetic data [19]. We show that it is possible to assign kinship with reasonable accuracy for relationships dating back 100–300 years even for a population that exhibits high levels of homozygosity.

## Results

### Population Structure among Ethnolinguistically- and Continentally-defined Groups

**Figure 1:**
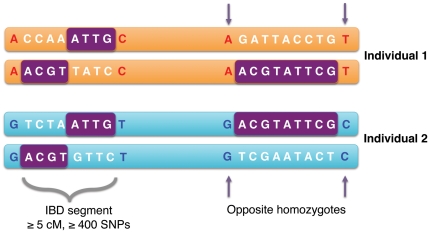
Schematic of IBD_half_ inference method. IBD_half_ segments were inferred from unphased genotype data where a series of alleles were identical by state for *at least one* of the homologous chromosomes in a given pair of individuals. IBD segments are indicated in purple. The boundaries of the IBD segments are defined by “opposite homozygotes”. Additionally, an IBD region had to be minimally 5 cM in length and contains >400 genotyped SNPs that were homozygous in at least one of the two individuals being compared (see *Methods*).

To infer identity by descent, we scanned each pair of genomes for long runs of genotype pairs that lack “opposite homozygotes” (Figure 1). We define inferred “IBD_half_” as the sum of the lengths of genomic segments where two individuals share DNA identical by *state* for at least one of the homologous chromosomes (see also [20]). This method is computationally feasible in large sample sets of thousands of individuals because we do not need to phase individuals; phasing is necessary for other methods of accurately inferring short IBD segments (e.g. BEAGLE, GERMLINE [1], [4], [5], [21]). We find our approach is accurate down to 7 cM per pair when we simulate the number of false positives seen in only one member of a family a trio and an un-related individual (see *Methods*).

For each population we characterize the distribution of inferred IBD, after excluding individuals inferred to be first cousin or closer [22]. The values of mean IBD_half_, that is the average extent of inferred IBD_half_ across all pairs of individuals within a population, vary widely across a number of different ethnolinguistic and broadly-defined populations (Table 1). Tables 1 and S1 summarize genomic identity by descent for pairs of individuals sampled from 121 populations from across the globe. The large 23andMe population samples of individuals with European and Asian ancestry exhibit very low levels of mean IBD_half_, ranging from about 0.05 to 0.15 cM. In contrast, estimates of mean IBD_half_ for Native American population samples from the HGDP-CEPH set range from about 400 to 1900 cM. Apart from extremely high estimates of mean IBD_half_ in the Native American samples, there is no clear geographic pattern to the rankings of IBD_half_ for ethnic groups across different world regions. This lack of a geographic pattern suggests that mean IBD_half_ is largely independent of general levels of heterozgosity in the population [14]. The correlation between haplotype heterozygosity [23] and mean IBD_half_ is modest (r^2^ = 0.45), and that correlation is driven primarily by the inclusion of Native American samples (r^2^ = 0.14 without Native Americans, Figure S2A).

**Table 1:** IBD statistics for a subset of HGDP-CEPH and 23andMe population samples^a^.

Population	Region of Ancestry	Mean IBD_half_ b	Sample size	F_IBD_ c	Total Non-zero Pairs	Source
Surui	S. America	1870.5	8	1.00	28	CEPH/HGDP
Karitiana	S. America	1229.5	14	0.88	80	CEPH/HGDP
Kalash	C. Asia	260.0	23	1.00	253	CEPH/HGDP
Yakut	E. Asia	85.4	25	0.92	276	CEPH/HGDP
Biaka Pygmies	C. Africa	73.2	21	0.96	202	CEPH/HGDP
Maya	N. America	47.3	21	0.47	99	CEPH/HGDP
Sardinian	Europe	12.4	28	0.38	143	CEPH/HGDP
Tuscan	Europe	9.3	8	0.43	12	CEPH/HGDP
Ashkenazi	Europe/Near East	23.0	847	0.85	304,539	23andMe
Finland	Europe	10.0	149	0.53	5844	23andMe
Yoruba	W. Africa	1.0	21	0.06	12	CEPH/HGDP
Canada	Mixed	0.6	373	0.04	2775	23andMe
Han Chinese	E. Asia	0.3	44	0.01	13	CEPH/HGDP
Italy	Europe	0.1	386	0.01	743	23andMe

a See also Supporting Information, Table S1, for IBD statistics in all 121 populations.

b “IBD_half_” is defined as the sum of the lengths of genomic segments where two individuals are inferred to share DNA identical by state for at least one of the homologous chromosomes.

c “F_IBD_” is defined as the fraction of pairs that share at least one IBD_half_ segment greater than or equal to 7 cM.

**Figure 2:**
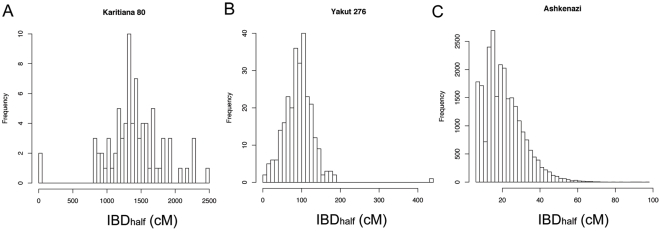
Distributions of IBD_half_ for pairs of individuals within three human populations. The average amount of DNA that is identical by descent (mean IBD_half_) varies widely across HGDP-CEPH, European, Asian and Ashkenazi populations. We present distributions of pairwise comparisons with IBD_half_ segments ≥7 cM for the (a) Karitiana Native Americans, (b) Yakut of Siberia, (c) Ashkenazi Jews primarily from the United States. Prior to the analysis, individuals were eliminated in order to remove close relationships (sibling, parent-child, avuncular, grandparent-grandchild, and 1st cousin pairs) (see *Methods*). Pairs with less than 7 cM IBD_half_ are not displayed. Distributions of IBD_half_ for additional HGDP-CEPH samples are presented in Supplementary Material (Figure S1).

Figures 2A–2C and S1 present distributions of IBD_half_ for all pairs of individuals within our sampled population. We define “F_IBD_” as the fraction of pairs within a population that share non-zero IBD, standardized by the total number of pairwise comparisons. Population samples where only a few pairs show evidence of IBD, that is where F_IBD_ is less than 25%, have pairwise distributions that come close to being exponentially distributed (Figure S1). However, some population samples, including several Native American groups (Figure 2A), the Yakut (Figure 2B), and the Kalash, are largely composed of pairs with IBD_half_ greater than 7 cM. Almost all pairwise values of IBD_half_ within the Native American Karitiana, Pima and Surui samples fall between 500 and 2500 cM (Figure 2A, Figure S1). The Mayan IBD_half_ values, in contrast, appear to be exponentially distributed (Figure S1). These differences across the mean IBD_half_ distributions of the Native American groups suggest that the IBD patterns reflect either recent population endogamy or non-random population sampling rather than the ancient population founder event that occurred during initial migration into the Americas, which would have influenced all populations similarly [24], [25].

### Assigning Degrees of Kinship using IBD

**Figure 3:**
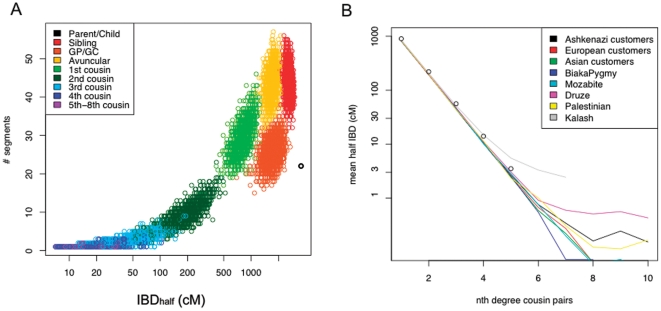
Relationship between degree of cousinship and IBD_half_ metrics. We used pedigree-based simulations to characterize the relationship between IBD_half_ metrics and degrees of cousinship for multiple population samples. a) Genomic data from a European sample were used to simulate an 11-generation pedigree. The joint distribution of IBD_half_ and number of IBD_half_ segments is shown for each pairwise comparison from the pedigree simulations. GP/GC indicates grandparent/grandchild pairs. b) For each of eight populations, we summarize the distribution of IBD_half_ by plotting IBD_half_(n) for the population by degree of cousinship. The degrees of cousinship distinguished by IBD_half_(n) asymptotes at different levels of IBD in ethnolinguistically-defined populations. Simulations were run on phased samples from several HGDP-CEPH population samples and European, Asian and Ashkenazi samples from a 23andMe customer dataset. Simulations were conducted by specifying an extended pedigree structure and simulating genomes for the pedigree by mating individuals drawn from a pool of empirical genomes (see *Methods*).

We simulated genetic data within extended family pedigrees in order to characterize the correspondence between the number of IBD_half_ segments or IBD_half_ and the degree of cousinship for a pair of individuals from the same population. Drawing from pools of computationally-phased haplotypes from HGDP-CEPH and 23andMe population samples, we simulated extended family pedigrees of 11 generations assuming random mating of individuals from the sample (see *Methods*). Using the specified family pedigree and the data simulated on the basis of that pedigree, we estimated the number of shared segments and IBD_half_ for cousinship degrees of *n* (where *n* = 1–10) (Figure 3A). Our simulations suggest that IBD_half_(n) decreases as the *n*th cousinship increases from 1st to 5th cousins (Figure 3B). The decay in mean IBD_half_(n) asymptotes at different levels across a set of ethnolinguistically-defined populations, starting at *n* = 5 (e.g., Kalash) (Figure 3B). Therefore, in populations with moderate to high mean IBD_half_ (Table 1), IBD_half_ alone cannot be used to distinguish accurately between 5^th^ or greater cousinships.

**Figure 4:**
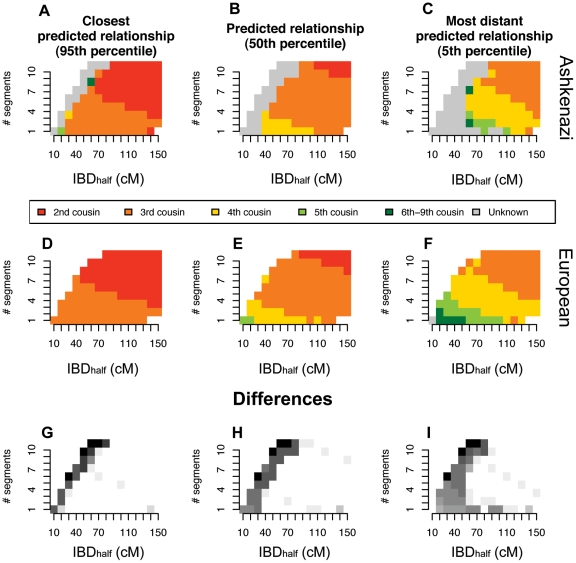
Distributions of IBD_half_ by degree of cousinship, assessed with simulated pedigrees for Ashkenazim and Europeans. Plotted, for each combination of IBD_half_ and number of IBD segments, are the 95th percentile, 50th percentile and 5th percentile degrees of cousinship based on 1 million simulated pedigrees. A–C) Ashkenazi pairs, D–F) European pairs, G–I) The differences between Ashkenazi and European results, presented in the prior panels, are represented in grey. Darker grey indicates higher number of differences. Each *n*th cousinship category was scaled by the expected number of *n*th degree cousins given a model of population growth (Table 2, *Methods*). Simulations were conducted by specifying an extended pedigree and creating simulated genomes for the pedigree by mating individuals drawn from a pool of empirical genomes. Pairs of individuals who appear to share IBD_half_ that was not inherited through the specified simulated pedigree are marked in grey in the A–F panels.

We explored in greater detail the data simulated for 2^nd^–9^th^ cousins based on the Ashkenazi (Figure 4A–C) and European (Figure 4D–F) samples. These figures reveal that there is a relatively clear relationship between IBD_half_ plus number of IBD segments and degree of cousinship for 2nd–5th cousinships. For example, the median relationship for a pair of Ashkenazi individuals with IBD_half_ of 100 cM and >9 segments is 2^nd^ cousinship, with 1^st^–3^rd^ cousin bounds (Figure 4B, 95% and 5% bounds presented in Figure 4A,C). For pairs from both populations, 3^rd^ cousinships encompass a wide range of segmental variation, generally greater than 25 cM IBD_half_ and fewer than 10 distinct IBD segments (Figure 4B,E). For the European sample, just one or two shared segments totaling less than 15 cM in length yield a median relationship of 5th cousin (Figure 4E). However, low amounts of IBD_half_ (e.g., less than 4 segments totaling less than 40 cM in length) in simulated Ashkenazi pairs were not associated with an identifiable cousinship within our simulated pedigree (Figure 4A–C).

### Expected and Predicted Distant Cousins

The relationship between IBD metrics and degree of relationship indicated in Figures 3 and 4 can be leveraged to predict degree of relationship on the basis of IBD metrics. After quantifying the bounds of IBD sharing for 1^st^–9^th^ cousins for European and Ashkanazi population samples, we determined how common it was to find a distant relative in a heterogenous database of a specified size. In order to estimate the number of individuals who have a *distant* relative in the 23andMe dataset, we first removed individuals in pairs with close kinship (i.e., parent/child, sibling, grandparent/grandchild, avuncular, 1st cousin). Then, for all pairs of individuals in each of the European and Ashkenazi samples, we counted the fraction of pairs with IBD_half_ ≥7 cM. Sample size was initially restricted to n = 300 for each population.

The fraction of 23andMe individuals with at least one predicted 2^nd^–9^th^ degree cousin varied across the European and Ashkenazi populations. Virtually all (99%) individuals of Ashkenazi ancestry had a least one relative detectable in the 23andMe dataset. Interestingly, 70% of individuals in our 23andMe European sample were also predicted to have at least one 2^nd^–9^th^ cousin (or similarly distant relationship) even with a dataset size as small as 300.

**Figure 5:**
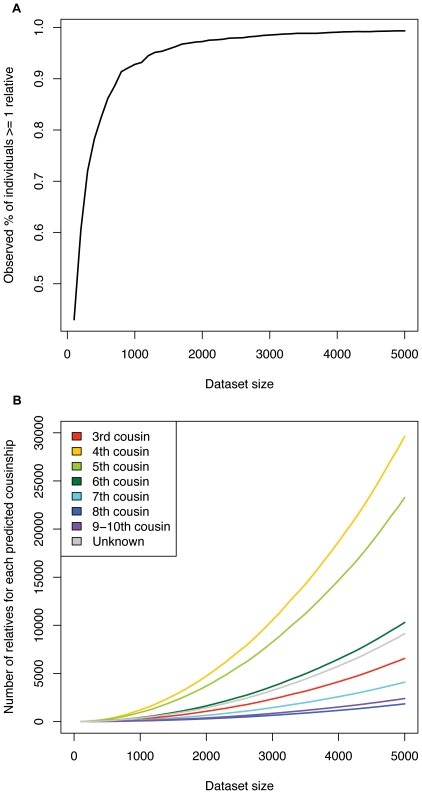
Fraction of 23andMe individuals with detectable distant relatives within subsamples inferred using IBD_half_. A) The fraction of individuals with at least one predicted relative (2^nd^–9^th^ cousin) given datasets of varying size. All datasets were drawn from a dataset of 5000 individuals with European ancestry. All closely related individuals (i.e., 1^st^ or 2^nd^ generation family) were removed before performing the analysis. B) The number of predicted cousins of each degree of cousinship given the dataset size. Predictions based on parameters obtained from simulations (Figure 4e).

In order to examine the relationship between database size and number of detected cousins per individual, we assembled 5,000 individuals with European ancestry and randomly drew subsets of this dataset (Figure 5A). For each subset we calculated the fraction of individuals with at least one relative (2^nd^–9^th^ degree cousin) given an IBD_half_ threshold of ≥7 cM. As the dataset size increased, the fraction of individuals with at least one distant relative increased logarithmically (Figure 5A). With a sample size of 1,000 heterogenous Europeans, more than 90% of individuals had a predicted cousin in the 2^nd^–9^th^ degree range. With a sample size of 5,000 individuals, virtually every individual had at least one predicted relative. We also catalogued the number of individuals for each *n*th degree of cousinship for datasets of increasing sizes in our database (Figure 5B). The 4^th^–6^th^ cousinships made up the bulk of predicted relationships; there were approximately 30,000 predicted 4^th^ cousin pairs in a dataset of 5,000 Europeans (Figure 5B). The ranking of the number of detectable cousins (Table 2) differs from our ranking of observed cousinships (Figure 5b) [please note that N^dc^ refers to the number of expected cousins for a single individual, while Figure 5b shows the observed number of cousin pairs.] This difference in the ranking of the expected number of detectable cousins and the observed number of cousin pairs in our dataset may be due to: deviation from the assumed 2.5 mean number of historical offspring in our model, higher than expected IBD due to sharing multiple ancestors rather than a single common ancestor, and variability in the recombination map. In sum, there are an extensive number of cousins for any given individual sampled from a large population (Table 2).

**Table 2:** Expected extent of IBD and number of cousins for 1st–10th degrees of cousinship.

Degree of cousinship	Expected amount of IBD (cM)^a^	Chance of detecting *n*th cousin (%) with IBD_half_ b	Expected number of cousins^c^	Expected number of detectable cousins (N^dc^)^d^
1	900	100	7.5	7.5
2	225	100	38	38
3	56	89.7	190	170.4
4	14	45.9	940	431.5
5	3.5	14.9	4,700	700.3
6	0.88	4.1	23,000	943
7	0.22	1.1	120,000	1,320
8	0.055	0.24	590,000	1,416
9	0.014	0.06	>10^6^	NA^e^
10	0.0034	0.002	>10^6^	NA^e^

a Theoretical expectation of the amount of IBD across the genome shared between *n*th cousins, assuming 3600 cM across the entire genome. It should be emphasized this description assumes a single common ancestor for a pair of cousins; multiple shared common ancestors will increase the predicted IBD sharing.

b The fraction of *n*th degree cousins detected using our IBD algorithm and based on simulated pedigrees of up to 10th degree cousins (see *Methods*).

c Assuming a specific model of pedigree and population growth over the past 11 generations (see *Methods*).

d The expected number of cousins detectable with our IBD algorithm (N^dc^) was calculated by multiplying the probability of detecting an *n*th cousin by the number of *n*th cousins obtained from our pedigree model of population growth (see *Methods*).

e Given the variation in population growth at >9 generations ago, combined with a low power of detection for 9th or 10th cousins, we have indicated the number of detectable cousins for those categories as not applicable, “NA”.

We then compared the distributions of predicted kinship with expectations based on population history and database sampling. Specifically, we modeled the number of cousins (2^nd^–9^th^ degree) expected for any given individual (*Methods*). The model predicted that 99% of Ashkenazim and 91% of individuals with European ancestry would have at least one 2^nd^–9^th^ degree cousin in the datasets representing 300 and 5000 individuals, respectively. These predictions closely matched our observed values: 99% for Ashkenazim and 99% for Europeans. The predictions are robust to several choices of parameters (i.e., number of children per generation, shared ancestors [not shown]), but the choice of N_pop_ parameter (i.e., the current size of the pool of potential relatives) significantly influences the theoretical predictions (Table S2).

## Discussion

For any given set of genome-wide genotype data for a population sample, one can observe the number and length of DNA segments shared IBS across the entire genome for a pair of individuals and, from these observations, infer IBD. Our simulations indicate that one can then predict the degree of cousinship for each pair of individuals, at least when our IBD_half_ statistic is sufficiently large (Figure 4). The total length of homologous identical by descent DNA segments that two individuals share is similar to a classical metric, runs of homozygosity (ROHs), which are inferred by comparing allele states extending along a locus within an individual. ROHs are readily calculated without phasing of genotype data, and when averaged over all individuals in a sample, have been used successfully to track population processes, such as consanguinity [8], [13], [26], [27]. An advantage of using a pairwise IBD approach rather than ROHs is that pairwise IBD comparison yields discovery of many relatives within a population, whereas ROHs only allow inference of relatedness for an individual's parents.

### Population Structure among Ethnolinguistically- and Continentally-defined Groups

The characterization of genomic similarity across samples of the HGDP-CEPH collection has revealed about 100 cryptically related or duplicate samples [18], [22]. Prior analyses focused on identifying familial relationships such as sibships, parent-offspring pairs, etc. (i.e., 1^st^–3^rd^ degree relatives). We extended this analysis through pairwise IBD comparisons and characterized pairs of individuals who are related as 1^st^ through 9^th^ cousins within each HGDP population (Figure 2, Figure S1). When datasets include samples from populations where many of the individuals are closely related, analyses of population structure tend to cluster these populations more discretely than populations with greater genetic variation. For example, previous analyses of genome-wide microsatellite data identified the Kalash of Pakistan as a distinct global population in a *STRUCTURE* analysis of HGDP, k = 6 [28]. Our pairwise IBD metrics suggest an explanation for this result; the Kalash share on average 260 cM IBD_half_ (Table 1) and thus any given pair of individuals is the genomic equivalent of second cousins. In both microsatellite and SNP genotype-based *STRUCTURE* plots, Native American HGDP populations emerged as a population subset at k = 4 [23], [28]. Our IBD_half_ estimates for the Native Americans (Figure 2A, S1, Table 1) are consistent with an interpretation that the majority of individuals within these populations are related as the genomic equivalent of 2^nd^ cousins or closer. We note that although putative close relatives were removed with the HGDP952 dataset, our 700 cM cutoff employed for the 23andMe dataset would have removed most pairs in the Native Americans populations (though not from most HGDP groups, see Figure S1). Populations such as the Karitiana or Pima display levels of mean IBD_half_ that are 3–4 orders of magnitude higher than many HGDP Asian populations such as the Han, Japanese, Mongolians. As such, population genetic analyses of the HGDP-CEPH Native Americans will need to account for the IBD derived from their elevated levels of historic endogamy or uneven population sampling.

The high levels of mean IBD_half_ and F_IBD_ of many of the 121 globally distributed populations we analyzed are consistent with substantial structure among populations within continents. (Table 1, Table S1). On the other hand, populations that were sampled randomly across a wide ethnolinguistic and geographic space (i.e., 23andMe European and Asian continental samples, Table 1) have very low estimates of mean IBD_half_. The elevated amount of IBD_half_ in the majority of ethnolinguistic populations from HGDP-CEPH, and in some 23andMe sub-continental samples, compared to that of samples of general European or Asian ancestry is indicative of small effective population size, often reflecting endogamy. Our IBD metrics complement and extend other genomic analyses, such as principal components analysis or clustering algorithms, that have recently indicated fine-scale population structure within continents [16], [29], [30]. We show that IBD distributions can be used to characterize patterns of kinship within groups over the past 100–300 years, and these genetic-based indications of endogamy can be helpful for explaining the processes generating population structure. Our results also suggest that exploring *within*-population structure, occurring during the last few hundred years, may be fruitful avenue for future research [31].

### Differences in IBD Metrics Across Ethnolinguistically-defined Populations

Although there is no clear geographic patterning, populations practicing hunting-gathering or pastoralist subsistence strategies tend to have higher mean IBD_half_. African hunter-gatherers, former hunter-gatherers from South America, Yakut, Mozabite Berber, and Bedouin pastoralists are ranked in the top 50% of our sample set in terms of mean IBD_half_ estimates (Table 1). A high estimate of mean IBD_half_ suggests that the population has a relatively small effective size or has been sampled in such a manner as to over represent related individuals. Small effective size for human populations often reflects a high level of endogamy, a population bottleneck, or both.

In order to illustrate the effect of recent demographic fluctuations on IBD distributions, we highlight two populations: the Yakut and Ashkenazi Jews. The Yakut, a population of nomadic pastoralists in Siberia, have an IBD_half_ distribution that is approximately normally distributed, with a mean of 85 cM (Figure 2b). Recent demographic inference from mitochondrial DNA simulations suggests that a small group of individuals (∼150 females) in southern Siberia founded the ancestral Yakut population only about 1,000 years ago (ya) [32]. Their IBD_half_ distribution includes a few low identity pairwise comparisons (8%) and the remaining pairwise comparisons are more normally distributed (F_IBD_ = 92%, Figure 2b). The high proportion of pairs of individuals who share some fraction of their genome IBD is consistent with the results from mtDNA mismatch simulations indicating that the Yakut experienced a founder event occurring within the past 1,000 years, followed by population isolation.

Historically Ashkenazi Jews have had endogamous mating patterns and are thought to have experienced two major founder events approximately 650ya and 2,000ya [33], [34]. We studied a set of over 150 individuals in the 23andMe database who reported having four Ashkenazi grandparents. Mean IBD_half_ is in the bottom quartile of our population samples, 8 cM, similar to that of other European populations such as the Tuscans or Sardinians. The F_IBD_ statistic (F_IBD_ = 37%) for the Ashkenazi is similar to that of many other single ethnolinguistically-defined European or Asian populations (Table S1). Although the Ashkenazi and the Yakut have similar purported population histories, their patterns of genome-wide IBD differ substantially. The differences may reflect different initial population sizes or recent gene flow into the Ashkenazim.

### Assigning Degrees of Kinship using IBD

Through simulation we demonstrated that it is possible use IBD_half_ to accurately predict a probable level of kinship for 2^nd^–5^th^ cousins. Cousinships between 6^th^–9^th^ degrees are assigned less accurately due to varying levels of background relatedness in different populations and stochasticity in IBD segment lengths over many generations (Figure 3 and Figure 4). We focused on the metrics IBD_half_ and number of IBD_half_ segments shared by a pair of individuals in order to identify recent common ancestry. We verified the accuracy of the IBD_half_ segments by checking whether the segment was found in either of the simulated parents of the pedigree. The paucity of predictions at high *n*th degrees suggests that our IBD_half_ metrics and IBD algorithm is accurate only within the bounds of 1^st^–9^th^ cousins.

A large fraction of simulated Ashkenazi pairs shared IBD_half_ segments that showed no descent through the common ancestor specified in our simulated extended pedigree (Figure 4a–c, i.e., indicated by “unknown”). We initially assumed that all pairs of Ashkenazi used to seed the simulations were unrelated (after pruning close relatives, *Methods*). However, the high fraction of Ashkenazi pairs with short IBD_half_ segments (5–20 cM, Figure 2C) indicates that many individuals in our dataset are already related on the order of 2^nd^–9^th^ cousins. Thus, a random sample of Ashkenazim, such as that incorporated into our simulations, includes pairs of individuals who share multiple, long segments that are identical by descent and whose inferred relationship, in some cases, is more recent than the cousinship specified in the simulation. Ancestry on the order of 2nd–9th cousins, or about 200 years, is considerably more recent than a common ancestor dating back to the proposed founder event for the Ashkenazim 1,500 years ago. This finding illustrates the impact of endogamy and possibly non-random mating (i.e., sub-structure) within the Ashkenazi population over the past few hundred years on patterns of genetic variation.

Alternatively, the pattern of “unknown” matches may reflect the high homozygosity among the Ashkenazim. Short, distinct but overlapping IBD segments that would otherwise not be detectable by an IBD algorithm can appear together as a longer, detectable tract of IBD with another “unrelated” individual. Such matches reflect identity by state rather than identity by descent. Preliminary simulations suggest that this is a potential explanation for the excess “unknown” matches in the Ashkenazi population sample [35] (Figure 4).

### Expected and Predicted Distant Cousins

We determined how many individuals in a population sample have a predicted 2^nd^ to 9^th^ degree cousin in the same sample. We observed that 99% of Ashkenazim and 99% of Europeans have at least one distant relative (e.g., 2^nd^–9^th^ cousin) in the 23andMe datasets (of 300 and 5,000 individuals respectively). It is possible that our dataset is enriched for second cousins, as 23andMe customers may have suggested that extended family members join the 23andMe service. However, most IBD_half_ matches occurred with very low amounts of IBD_half_ (e.g., 10–20 cM) (Figure 5a) indicative of likely 3rd–9th cousinship (Figure 3, Figure 4). As most Americans are unlikely to know their 3^rd^ cousins or more distant relatives (i.e., 7 or more generational degrees of separation), the general finding is relevant to random sampling of individuals in these populations. Using identity by descent, Kong et al. [36] examined a 10 cM segment in the MHC region within a sample of Icelandic individuals. In a database of 6,300 samples, they found that 78% of Icelandic individuals share a long IBD segment with at least one other person. Both our results and those of Kong et al. [36] suggest that a large sample of individuals with European ancestry is likely to include many pairs of 2^nd^ through 9^th^ cousins.

Factors that contribute to the large number of relative pairs within moderately sized datasets include the large numbers of actual cousinships, especially beyond 4^th^ cousins (Table 2), and the small effective sizes of some populations. Given a model of 2.5 children per generation for every mating event, the number of relatives in the 5th through 10th degree cousinship categories runs from the thousands into the millions (Table 2). While we made a number of simplifying assumptions (i.e., perfect survivorship, non-overlapping generations), the expected number of detectable cousins between 1^st^ and 8^th^, (N_dc_) for a given individual is very high (Table 2). The expected number of individuals with at least one predicted relative in a dataset of a given size using our N_dc_ estimates, was similar to the observed values.

Additionally, the exponentially growing number of ancestors for any individual, constrained by finite population size means that the most recent common ancestor for any two individuals can be quite recent, especially in small populations. Indeed, Rohde et al. [37] used a spatially structured model to calculate that every pair of living humans has a most recent common ancestor as few as 200 generations ago. The number of individuals with at least one detected relative in our dataset varies between populations (Figure 5a). Ashkenazim are more likely than other Europeans or Asians to have at least one cousin in a dataset of 300 (99%) because the effective size of the Ashkenazi population is small. This small effective size most likely reflects population bottlenecks and low rates of migration into the population.

We predicted the number of distant cousins we could detect for a given individual with a moderately sized database given simulations and a model that takes into account average sibship size and effective population size (see below). We found that our expectations matched empirical results when we assumed that we are drawing individuals from population that is 10% of the actual US census sizes. Two confounding factors are relevant in the comparison of the number of observed and expected cousins: population size and population structure (i.e. random mating). We assumed panmictic mating in our calculation of the exponential number of ancestors for each individual. However, we did not assume panmictic mating for the pool of individuals who have opted in to the 23andMe customer dataset presented here; we reduced the population size to account for population stratification.

If we were to incorporate non-random mating (i.e. population structure) into our calculation for the number of ancestors for a given individual, then in small populations a pair of individuals would share multiple ancestors thus reducing the ancestral population size and consequently the number of *n*th degree cousins for that population. For timescales on the order of 10 generation and populations larger 100,000, a random pair of individuals will rarely have overlapping ancestors [10].

### Broad Significance

By identifying genomic segments that are inherited identically by descent we can characterize kinship among individuals within the last three hundred years. We observed tens of thousands of 2^nd^ to 9^th^ degree cousin *pairs* within a heterogenous set of 5,000 Europeans. The high frequency of such relative pairs in some populations is likely due to population bottlenecks followed by recent population growth over the past 10 generations and/or to endogamous mating within ethnolinguistic populations. Patterns of inferred IBD suggest, for example, that the Ashkenazi Jewish population has remained relatively small and endogamous over the last few centuries.

We emphasize that the high prevalence of IBD in many populations can advance disease association studies conducted within those populations. Similarly, disease phenotype mapping research has demonstrated the feasibility of homozygosity haplotype analysis in autism, Parkinson's disease and breast cancer studies, among others [38]. Homozygosity haplotype mapping identifies a disease locus with extended runs of homozygosity *within* an affected individual, indicative of a recent common ancestor for the segment. Homozygosity haplotype mapping studies have typically focused on autosomal recessive diseases and/or family pedigrees with known consanguinity. However, this approach can be extended to incorporate IBD between affected individuals, i.e., pairwise homozygosity mapping or relatedness mapping [39], [40]. Recent pairwise IBD analysis of individuals with breast cancer suggests that there are significant differences in local IBD between cases and controls [39]. We have demonstrated that, even among individuals from broadly-defined population samples, IBD segmental detection and inference of recent shared ancestry on the order of 10 generations is quite common (Figure 5). Thus, an IBD-based approach can facilitate disease association even in large genomic databases where the family pedigrees are unknown or individuals are considered unrelated.

## Materials and Methods

### Datasets

Our data consist of genotypes for 650,000 SNPs (publicly available) from the Illumina 650K platform typed in 52 worldwide human populations from the HGDP-CEPH collection [23]. Additionally, genotypes for about 580,000 SNPs typed on the 23andMe customized Illumina 550K+v1 or +v2 platforms were obtained from populations in the 23andMe customer database. All individuals provided informed consent and answered surveys online according to our human subjects protocol, which was reviewed and approved by Independent Review Consulting, now part of Ethical & Independent Review Services, a private institutional review board (http://www.eandireview.com). Consent was obtained electronically; in a subset of cases a waiver of consent was obtained. All consent processes were approved by the above-named institutional review board. In order to protect the privacy of 23andMe participants, the 580K data are not publicly available. We used the overlapping 550,000 SNPs from HGDP and 23andMe for analyses presented herein. We selected 5,000 unrelated individuals inferred to be of European ancestry, 300 inferred to be unrelated Ashkenazim, and 300 inferred to be unrelated East Asians. Most of these individuals were likely born in the United States. An additional set of # individuals is included in Table S1.

Ancestry was assigned using two methods. We used self-reported ancestry as obtained from customer surveys, where membership in a given ancestry was defined by an individual reporting all four grandparents born in the same country. For Ashkenazi Jewish, we identified a grandparent to be of Ashkenazi descent if this was stated in a field denoting ethnic identity. In order to increase the numbers of some subpopulations we also used principal components analysis to partition individuals into clusters. Individuals falling into predefined northern or southern European, Asian or Ashkenazi clusters were grouped for subsequent analyses. In order to obtain “unrelated” individuals, we removed close relationships (i.e., sibling, parent, grandparent, avuncular, 1st cousin) among 23andMe customer groups by employing a greedy search that chooses individuals who have less than 700 cM IBD shared between each person already in the set of conforming individuals. Similarly, we used the HGDP952 subset identified by Rosenberg [22] to eliminate closely related individuals (between siblings, parent-offspring, grandparents) among HGDP-CEPH individuals.

### Identification and Calculation of IBD Segments

**Figure 6:**
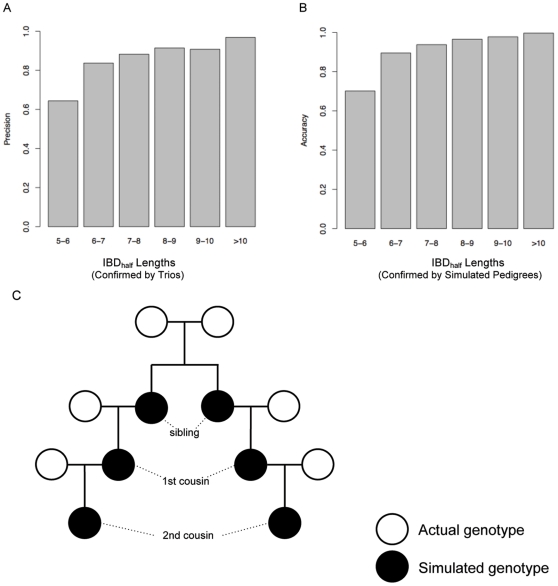
Precision and accuracy of implemented IBD algorithm. A) We considered how accurately we detect IBD segments that were transmitted between parent and child. We compared distant cousins with trios; we expect to observe sharing between a distant cousin and the child to also be observed in one of the parents for the same (or a longer) segment. Using this approach, we calculated the precision of our algorithm at different IBD segment lengths in a large sample of European-Americans. IBD segment lengths greater than 7 cM were observed 90% of the time in at least one parent. Preliminary data suggest that 7 cM segments shared between a distant cousin and child that were *not* observed in the parents were due to false negatives in the parents. B) We also examined our ability to detect IBD segments in simulated genotypes. After simulating large pedigrees, we examined 30,000 segments shorter than 200 cM resulting from 1^st^ to 10^th^ cousin relationships. We calculated the percentage of true IBD segments were detected by the IBD_half_ algorithm at different cM lengths. IBD segment lengths greater than 7 cM were detected over 90% of the time. C) This schematic illustrates the pedigree simulations, where actual genotypes reflect individuals randomly sampled from a given population and simulated children with known degree relationships were tracked. The simulated genotypes were then analyzed using the IBD algorithm (see *Methods* for additional details).

We inferred that two individuals share DNA IBD from unphased data. We inferred boundaries of IBD by comparing two individuals' genotypes at a locus and identifying SNPs where one individual's genotype is homozygous for one allele and the other individual's genotype is homozygous for a second allele. By characterizing stretches that *lacked* these “opposite homozygotes”, we defined regions that contain at *least half IBD* between two individuals (Figure 1, see also similar method in [36]). That is, an IBD_half_ segment was characterized by a series of alleles that were identical by state for at least one of the homologous chromosomes in a given pair of individuals. We define “IBD_half_” as the sum of the lengths of genomic segments where two individuals are inferred to share DNA identical by descent for at least one of the homologous chromosomes. We additionally enforced two criteria to increase our confidence that a region represents DNA that is IBD: first, the region is minimally 5 cM in length (Figure 6) and second, it contains >400 genotyped SNPs that are homozygous in at least one of the two individuals being compared, ensuring that there is both sufficient genotype coverage and genetic distance defining the IBD region. Finally, we accepted a comparison as IBD if the longest segment in the comparison was at least 7 cM (Figure 6, see below). Pairs of individuals sharing less than the above IBD were considered to have zero IBD_half_. A recently published method that accounts for LD between SNPs has accuracy for IBD segments as low as 2 cM, however this also requires phasing large datasets [4]. We used the June 2006 version of the HapMap genetic map for estimates of genetic distance [41]. This genetic map was used for all populations in our dataset.

We minimized the effects of genotyping error in several ways: 1) we removed genotyped SNPs from consideration (for all individuals) if there were a large number of Mendelian errors when comparing data from known parent/offspring trios (more than 1 in 1000), 2) we removed SNPs that had a high “no call” rate (>40% no calls at a site) or otherwise failed quality control measures, and 3) in a putative IBD segment, we allowed for one opposite homozygotes if there was a region of at least 3 cM, containing 300 SNPs, surrounding the opposite homozygote that were free of opposite homozygotes. Segments identified as sharing IBD via our IBD algorithm and criteria described above are referred to as “IBD_half_” segments in this paper; note however, the lack of opposite homozygotes in a segment is evidence for IBD across either one or both chromosomes.

### Accuracy of IBD detection algorithm

We assessed the accuracy of the IBD algorithm in two ways. First, we considered how accurately IBD segments were transmitted between parent and child. Given real IBD sharing, we expect that when comparing a distant cousin with a trio, sharing observed in the child should also be observed in one of the parents for the same (or longer) segment. We calculated the accuracy of the algorithm for a broad range of IBD segment lengths. IBD segment lengths greater than 7 cM showed 90% accuracy (Figure S6A).

We also compared the inferred IBD segments with IBD segments generated via simulation. By looking at a series of 30,000 simulated IBD segments shorter than 200 cM, we determined the percentage of IBD segments that were detected by our IBD algorithm for different simulated IBD lengths. IBD segment lengths greater than 7 cM were detected over 90% of the time (Figure S6B). Given the similar results of the two analyses, we concluded that a minimum longest segment length of 7 cM provided sufficient evidence for IBD and therefore removed pairwise relationships that did not meet this threshold in subsequent analyses.

### IBD metrics by population and pairwise distributions

We define mean IBD_half_ as average IBD_half_ across all pairs of individuals within a population sample. Considering 7 cM of IBD_half_ segment length identified between individuals to be a minimum, we calculated the mean IBD_half_ for all HGDP-CEPH and 23andMe customer populations. Many pairwise comparisons within a population were characterized by less than 7 cM IBD_half_; these pairs were considered to have zero DNA IBD. We define a F_IBD_ as the fraction of pairs within a population that share non-zero IBD, standardized by the total number of pairwise comparisons.

For each HGDP-CEPH and 23andMe customer populations we characterized the pairwise IBD distribution within each sample via mean IBD_half_, distributions of IBD_half_, and F_IBD_. We removed closely related individuals as described above, ensuring that the majority of individuals in each population sample should be only cryptically related.

### Simulating IBD patterns from different genomic datasets

We expected that for different populations in our dataset the amount of DNA shared IBD by *n*th cousins would depend upon the amount of inbreeding the population has experienced in its recent past. To calculate population-specific expectations for the amount of DNA shared IBD between distant cousins, we simulated inheritance along extended pedigrees for *each* population of interest, starting from reference genotype data of known ancestry, and resulting in simulated genotypes of individuals with known relationships (Figure 6C). We generated a pedigree for pairs of *n*th cousins in the following manner: we began with two parents, at the top of the pedigree, who had two children. The two children then mated with two randomly-chosen individuals, sampled without replacement, from the population sample and produced only one child per union, resulting in a pair of individuals who are first cousins. By mating each of the two first cousins with a randomly-chosen individual from the population, producing exactly one child in each union, we obtained second cousins. Further repetitions resulted in more distant simulated cousins of known degree. Our method assumes that the composition of currently available genomes is representative of genomic diversity throughout the past 200 years, because we paired contemporary individuals with simulated ones throughout the different generations of the pedigree.

To simulate data for a pair of *n*th cousins, we required 2*n*+2 phased autosomal genomes. We used SNP genotypes from the 23andMe database that were of inferred Ashkenazi ancestry and European ancestry and from a subset of the HGDP-CEPH populations (see *Datasets* above). Genotype data were phased using version 3.0 of BEAGLE [42]. Data were phased in groups of 1000 subjects, grouped by ethnicity. Within each group, chromosomes were split into regions of 10,000 SNPs (with a 1000 SNP overlap between adjacent regions). All regions were phased using the default settings of BEAGLE. The resulting overlapping haplotypes were resolved into entire chromosomes by selecting the orientation that minimized differences between the overlapping haplotypes. Each population sample was screened for parent-offspring pairs, 1st degree cousins, grandparent and avuncular relationships. Populations shown in Figure 2,3 were simulated separately. It is expected that phasing error would result in an underestimate of shared IBD since errors would introduce false breakpoints in a shared IBD segment.

With each mating specified by the pedigree, crossovers were introduced between the two haplotypes in each parent, and each parent then contributed one of their haplotypes to the offspring. Crossover breakpoints were distributed according to the HapMap fine-scale recombination map [41], where the number of crossovers per chromosome per generation was modeled as a Poisson random variable with mean equal to the total genetic distance spanned by that chromosome. The same genetic map was used for all populations in our dataset.

The simulated genotypes were then analyzed using the IBD algorithm. The detected IBD segments were then compared to the “theoretical” IBD segments, since we kept track of the breakpoint locations.

We ran the simulations across both the HGDP-CEPH and 23andMe population samples. For samples that had fewer than 22 genomes (required for generating a 10th cousin), we generated as deep of a cousinship as possible. For the 23andMe datasets, the simulation was repeated one million times for each population sample; for the HGDP-CEPH datasets, the simulation was repeated 1,000 times.

### Calculation of expected bounds of relationships using simulated data

We estimated the expected relationship range for a pair of distant relatives based on the simulation results. We first counted the number of times each relationship type led to different combinations of IBD_half_ (rounded to the nearest 10 cM) and number of IBD segments. Pairs of individuals with IBD segments that did not match the IBD segments in their simulated ancestors were categorized as “unknown”, reflecting the possibility of having a common ancestor not due to the simulated pedigree. Because the simulations generated an equal number of cousins per cousinship type, we additionally weighted each cousinship type by the expected number of cousins for each cousinship type in order to account for the fact that there are many more cousins at distant degrees of relationship than at close degrees (Table 2); we estimated the number of each type of cousins using a model of pedigree. For each amount of IBD_half_ and number of segments, we calculated a distribution of 1st through 10th cousins (and unknown cousins) using the simulation results and the pedigree model. From this distribution, we extracted relationships found at the 95th and 5th percentile, which represent the expected bounds of the relationship, as well as at the 50th percentile, which represents the median expected relationship.

### Calculation of expected number of individuals sharing DNA IBD

We developed theoretical expectations for the number of *n*th cousins for any given individual assuming an average sibship size, and, given these expectations plus simulation results, calculated the probability of detecting a cousin via inference of sharing of DNA IBD. We began by calculating the number of *i*th degree cousins “N_c_(*i*)” for one individual (equation 1). For each degree, “*i*” there are 2*^i^* possible pairs of ancestors, each of which can generate multiple children. For instance, for *i* = 1 (1^st^ cousin), the number of couples in the relevant ancestral generation is 2 (two sets of grandparents) and a 1^st^ cousin could descend from either the individual's mother's or father's siblings. If each set of parents produces some number of children “z”, then the number of cousins for one side of the family is equal to the product of the number of non-ancestral offspring of the ancestral pair (z−1) and the total number of ith degree cousins generated by those non-ancestral offspring (z^i^): 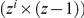. Therefore, N_c_(*i*) can be calculated as follows:
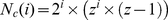
(1)We assumed, for z, bounds of 2–3 children per parent couple, where 2 children per couple is consistent with constant population size and 3 children per couple is equivalent to a population growth rate of 40% per generation, as discussed by Slatkin [20] for recent population history among the Ashkenazim. Estimates of population growth for Europeans and East Asians calculated using coalescent models tend to be closer to 2.01–2.15 children per couple, that is 0.7–7% growth per generation assuming 30 years per generation [43], [44]. We present results for the median estimate of 2.5 children per generation per couple (Table S2).

We then asked how many relatives should be detectable in a large heterogenous database using our IBD algorithm and data on 580,000 SNPs per individual. The fraction of detectable *i*th cousins “f(*i*)” was calculated based on results of simulations (described above). Those simulations yielded the fraction of *i*th cousins who share IBD greater than 5 cM and are therefore detectable using our IBD algorithm (Table 2). N_dc_(*i*), the number of detectable *i*th degree cousins, is defined as the product of the total number of *i*th cousins (N_c_(*i*)) and the fraction of those cousins that is detectable (f(i)):
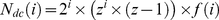
(2)We then calculated the probability of detecting at least one *i*th cousin for a given individual in a database of size N_db_. If the relevant population is of size N_pop_, the chance that a given member of the population is a detectable, *i*th cousin is N_dc_/N_pop_. Therefore the chance that a given member of the population is not a detectable *i*th cousin is 1−(N_dc_(i)/N_pop_). The chance that none of the individuals in a database of size N_db_ is a detectable *i*th cousin is (1−(N_dc_(*i*)/N_pop_))∧N_db_. The chance of detecting a cousin is, therefore:
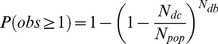
(3)


We assumed that the number of ancestors for a given individual increases at a constant rate going back in time. At some point in the past, the number of ancestors for two individuals overlaps and the number of ancestors for an individual ceases to grow exponentially. However, Derrida et al. [10] showed that the proportion of ancestors that overlap over 10 generations, given a large present-day population size (>100,000) and panmixia, is quite minimal. Thus, an exponential model is appropriate for our calculation of the number of ancestors for a pair of individuals when we assume random mating within populations throughout the past 10 generations.

In order to determine the relevant values of N_pop_, we first approximated the size of the Ashkenazi population of the United States as 6 million, and the size of the European-American population as 200 million [45]. Individuals who self-select for 23andMe may tend to have ancestry from particular subsets of the United States population. Ethnic ancestry is not homogeneously distributed throughout the United States [45]. For example, individuals from Maine are more likely to have multiple pairs of ancestors tracing back to England, while individuals from Wisconsin are more likely to have ancestry from Germany. Since our dataset is not a random subset of individuals from the European-American or Ashkenazi population, we then calculated reductions in population size from which potential relatives are drawn by 5^th^ percentiles of 200 million (see Table S2 for percentile calculations given our Ashkenazi data). Since the US population is unlikely to be characterized by truly random mating, we chose our N_pop_ to be 10% of US census sizes.

## Supporting Information

Figure S1 **Distributions of IBD_half_ for pairs of individuals within HGDP-CEPH populations.** The average amount of DNA that is identical by descent varies widely among HGDP-CEPH, European, Asian and Ashkenazi populations. We present distributions of pairwise comparisons with IBD_half_ segments ≥5 cM for all HGDP populations. Prior to the analysis, individuals were eliminated in order to remove close relationships (sibling, parent-child, avuncular, grandparent-grandchild, and 1st cousin pairs) (see *Methods*). Segments of less than 5 cM are not displayed.
(PDF)

Figure S2 **Comparison and correlation of HGDP-CEPH population statistics.** a) We compare the mean haplotype heterozygosity statistic obtained from Li et al. [23] with the mean IBD_half_ for each of 46 populations from HGDP-CEPH. Native American populations (Karitiana, Surui, Pima) were removed from the analysis due to their extreme levels of IBD_half_, indicating many close relatives within each sample. b) The length of runs of homozygosity (ROHs) for each individual were calculated following the same procedure as Nalls et al. [26]. ROHs were averaged for each population and presented as percent of the genome. We contrast the percent of the genome with ROHs to mean IBD_half_ for each population. The correlation between is driven primarily by populations with very high IBD_half_, such as Native Americans.
(TIFF)

Table S1 IBD and ROH statistics for HGDP-CEPH and 23andMe population samples.
(DOC)

Table S2 Detectability of Ashkenazi Relatives.
(DOC)
