# Supplementary material for: Cryptic Distant Relatives Are Common in Both Isolated and Cosmopolitan Genetic Samples
Source: PLoS One. 2012 Apr 3;7(4):e34267. doi: 10.1371/journal.pone.0034267 (PMC3317976; doi:10.1371/journal.pone.0034267)
Supplement: Figure S1 — Distributions of IBDhalf for pairs of individuals within HGDP-CEPH populations. The average amount of DNA that is identical by descent varies widely among HGDP-CEPH, European, Asian and Ashkenazi populations. We present distributions of pairwise comparisons with IBDhalf segments ≥5 cM for all HGDP populations. Prior to the analysis, individuals were eliminated in order to remove close relationships (sibling, parent-child, avuncular, grandparent-grandchild, and 1st cousin pairs) (see Methods ). Segments of less than 5 cM are not displayed. (PDF) [file pone.0034267.s001.pdf]

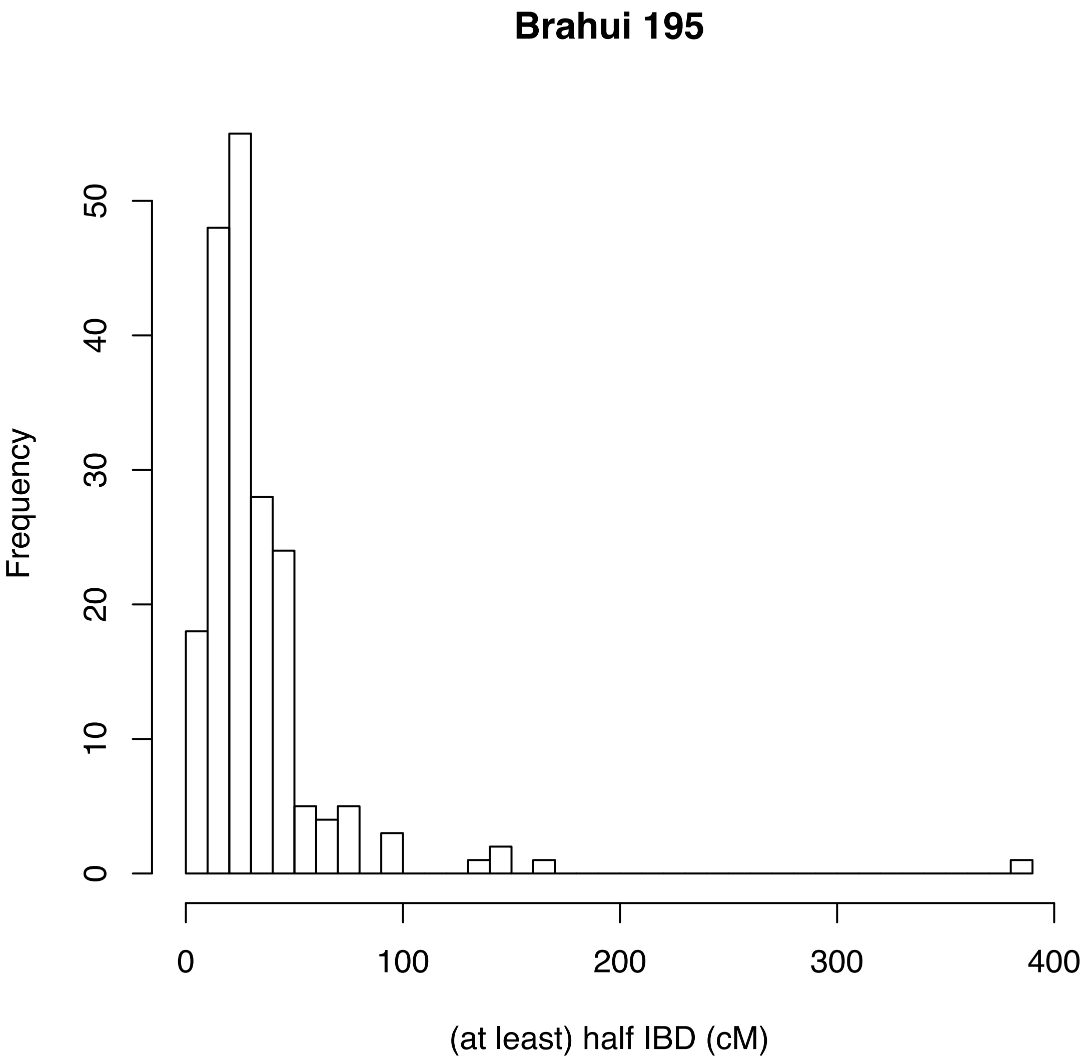

Brahui 195
Frequency
0
10
20
30
40
50
0
100
200
300
400
(at least) half IBD (cM)

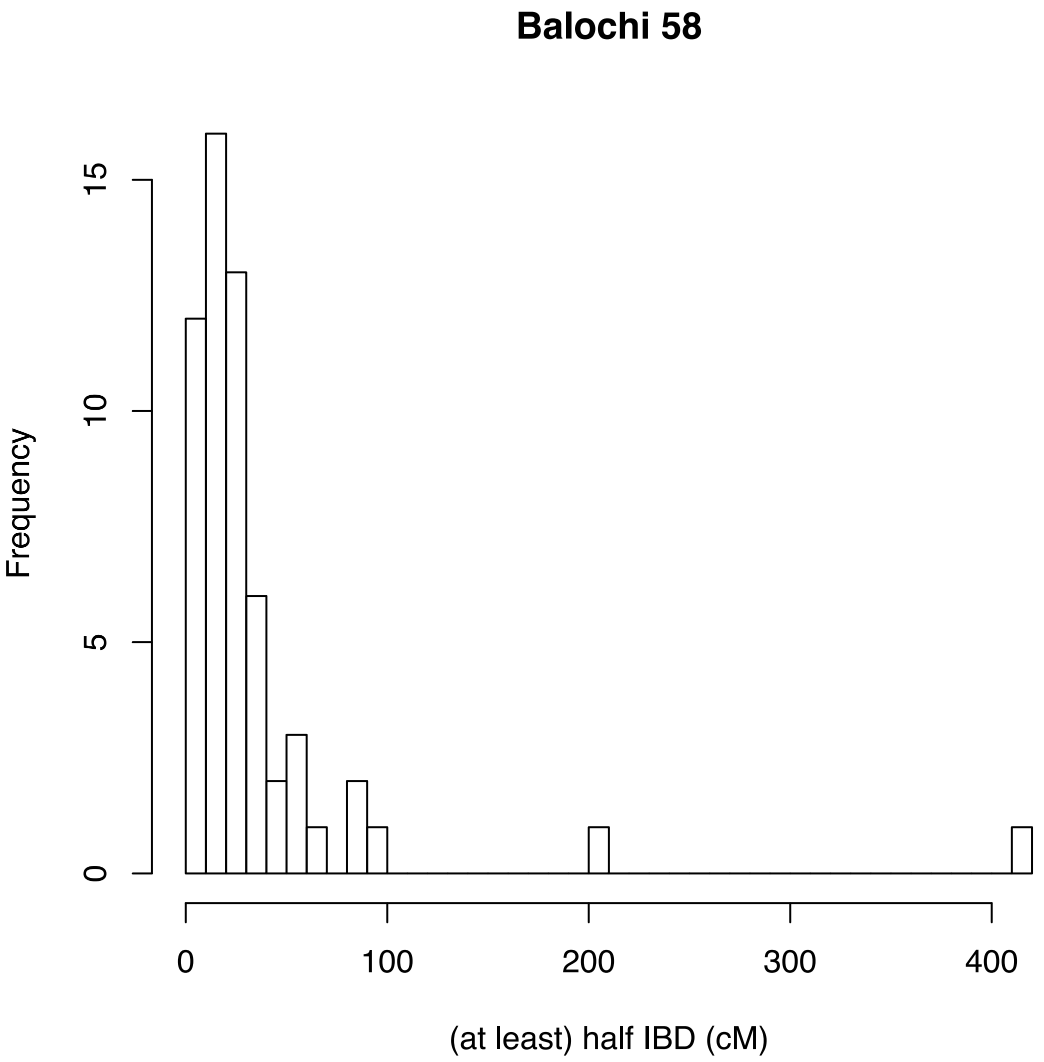

Balochi 58
Frequency
0
5
10
15
0
100
200
300
400
(at least) half IBD (cM)

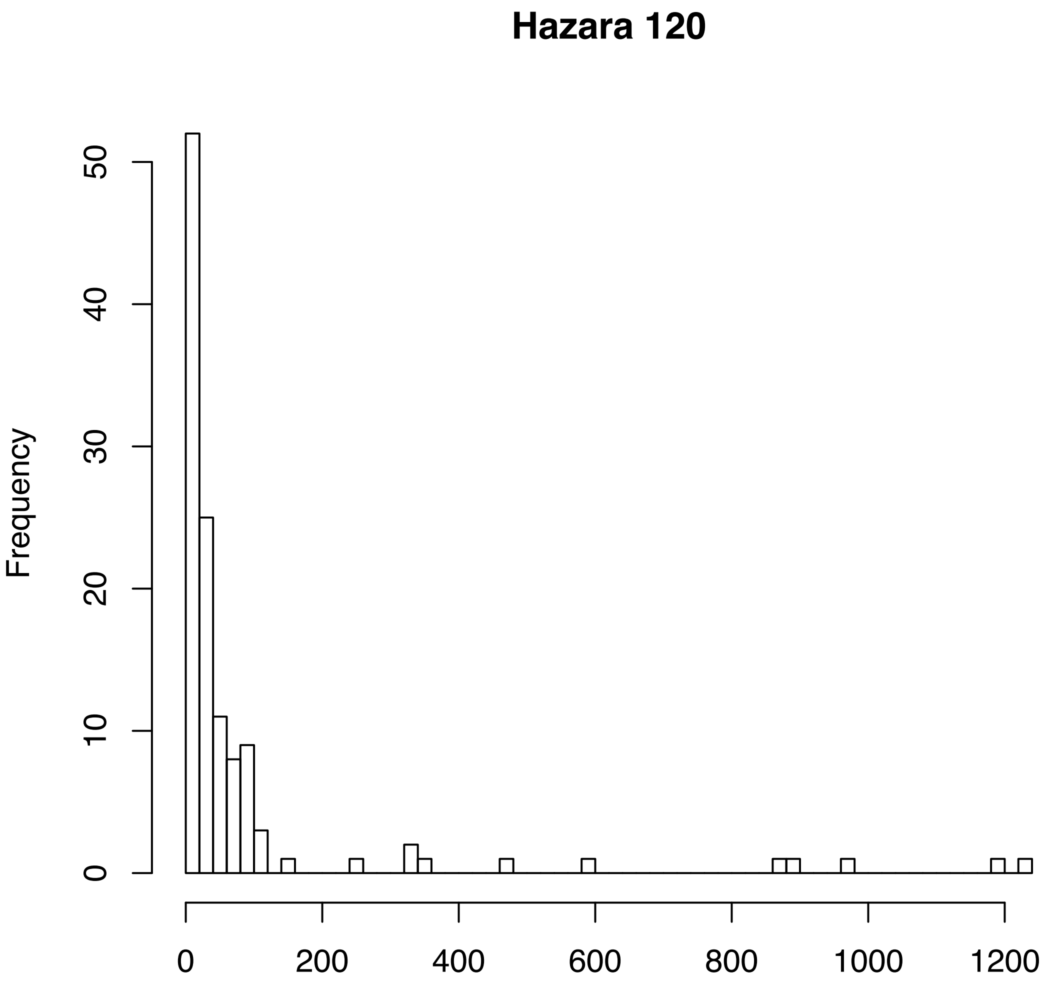

Hazara 120
Frequency
0
10
20
30
40
50
0
200
400
600
800
1000
1200
(at least) half IBD (cM)

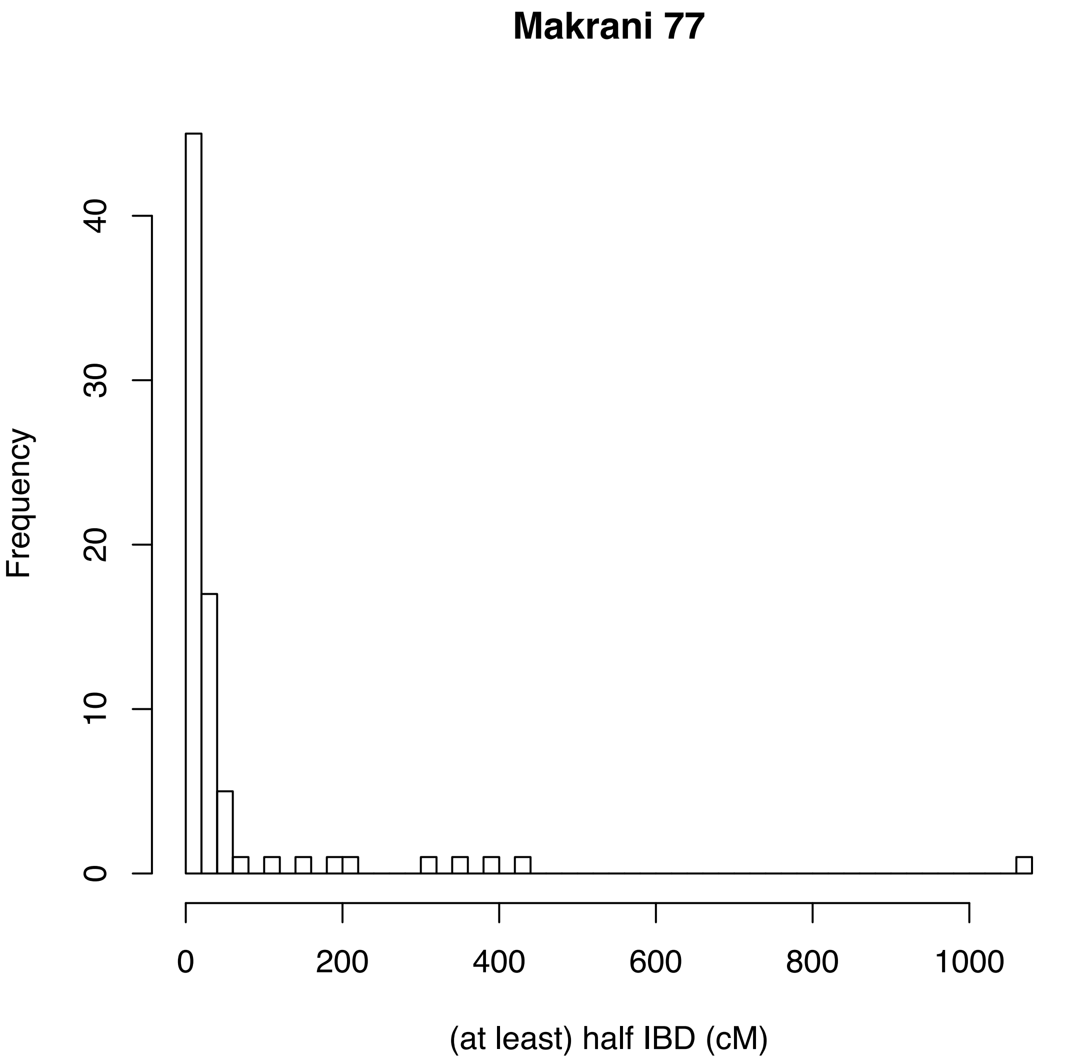
Makrani 77
Frequency
0
10
20
30
40
0
200
400
600
800
1000
(at least) half IBD (cM)

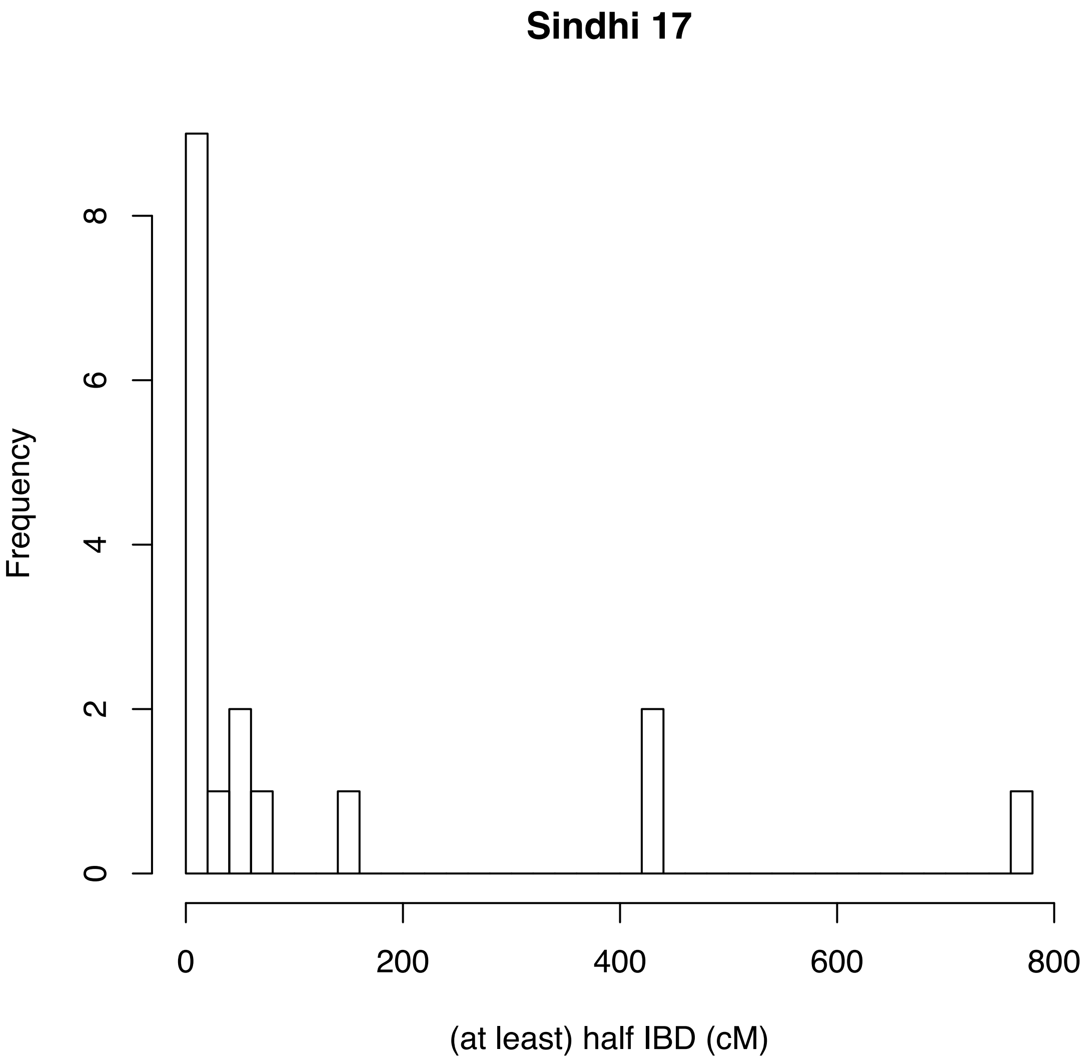

Sindhi 17
Frequency
0
2
4
6
8
0
200
400
600
800
(at least) half IBD (cM)

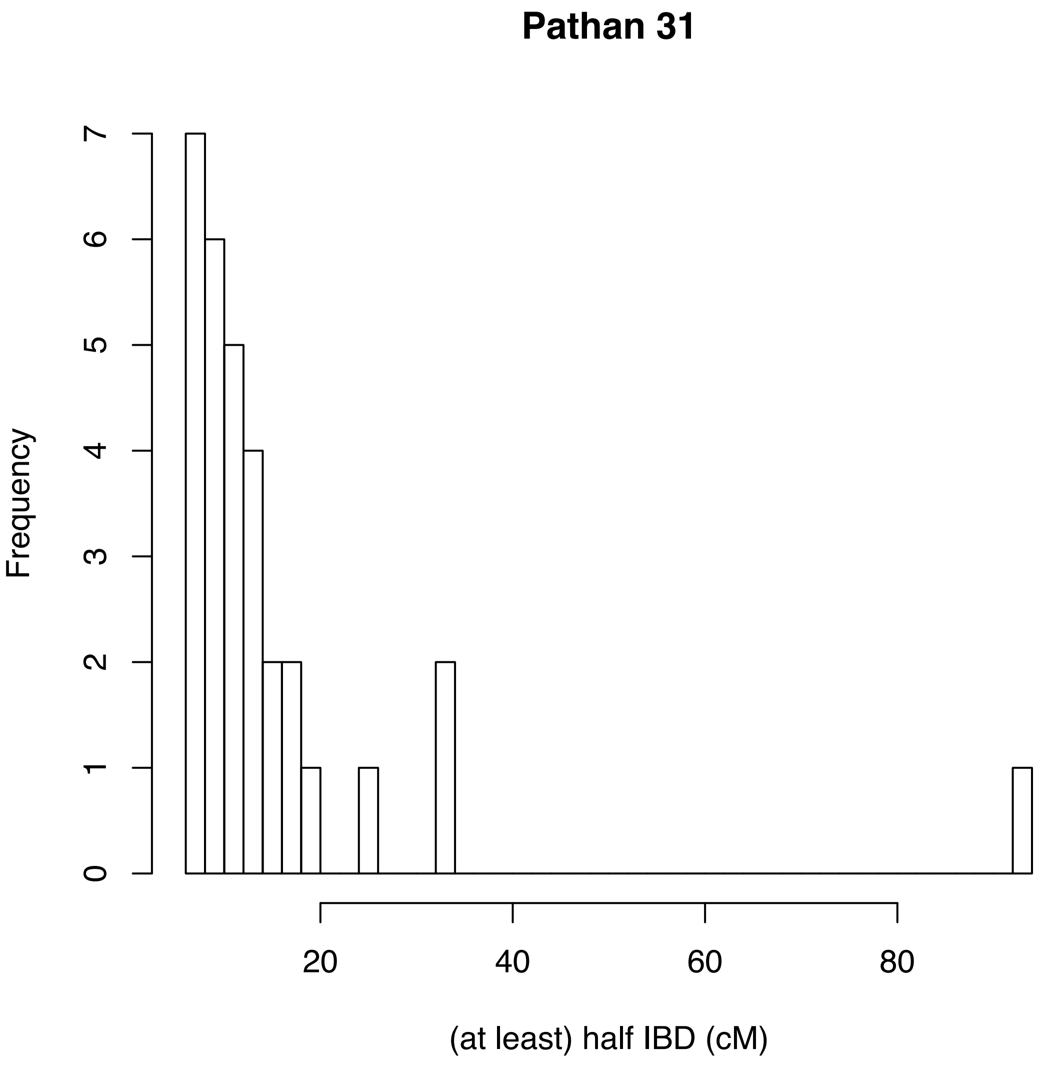
Pathan 31
Frequency
0
1
2
3
4
5
6
7
20
40
60
80
(at least) half IBD (cM)

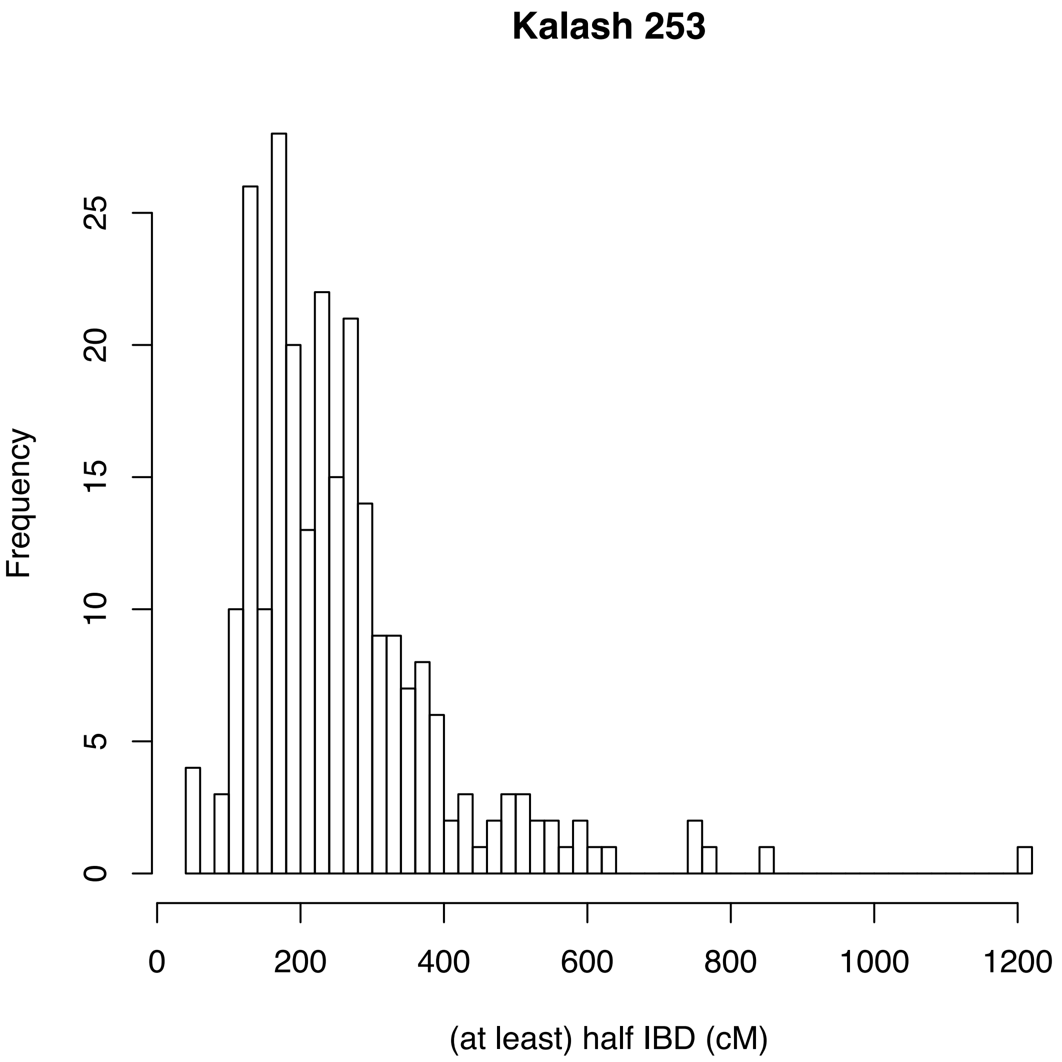
Kalash 253
Frequency
0
5
10
15
20
25
0
200
400
600
800
1000
1200
(at least) half IBD (cM)

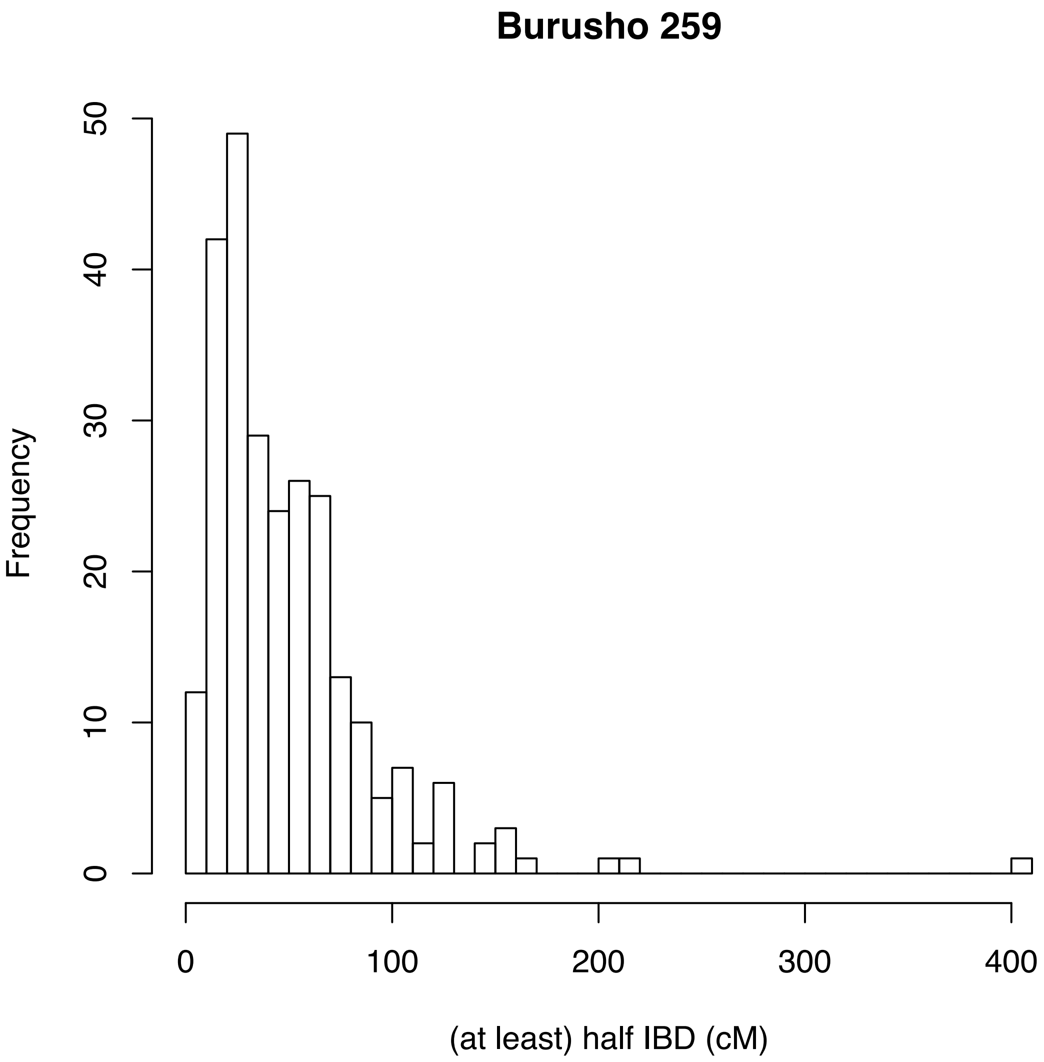

Burusho 259
Frequency
0
10
20
30
40
50
0
100
200
300
400
(at least) half IBD (cM)

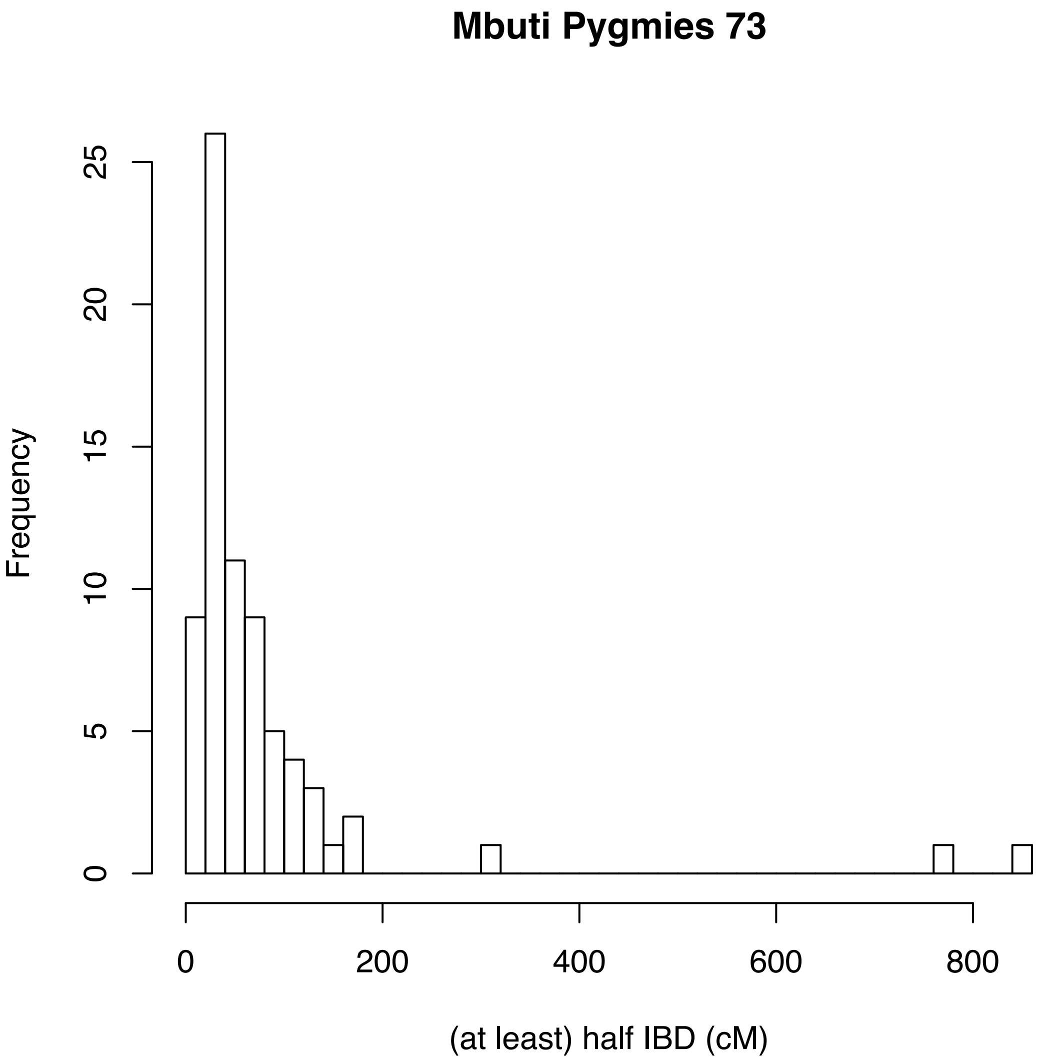
Mbuti Pygmies 73
Frequency
0
5
10
15
20
25
0
200
400
600
800
(at least) half IBD (cM)

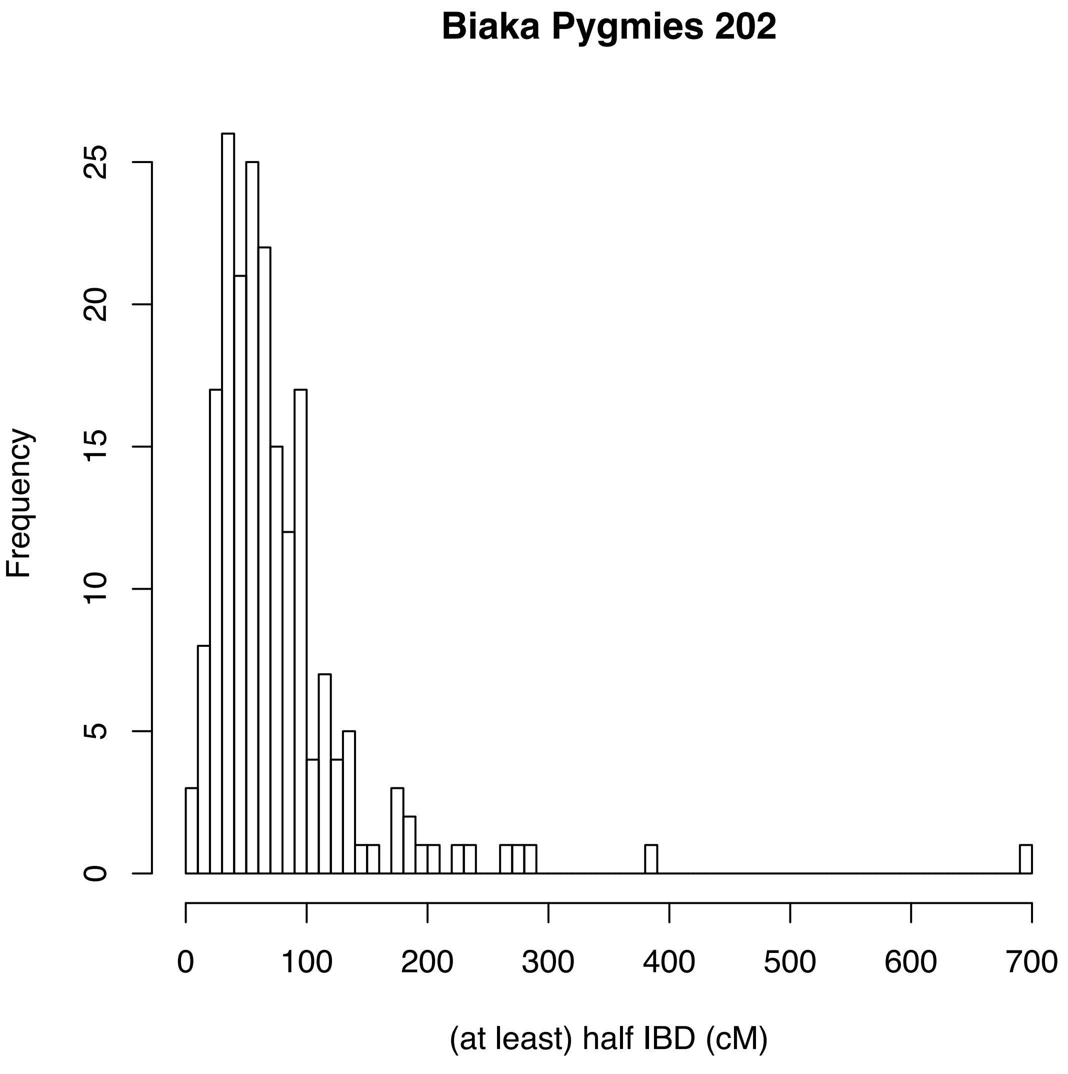

Biaka Pygmies 202
Frequency
0
5
10
15
20
25
0
100
200
300
400
500
600
700
(at least) half IBD (cM)

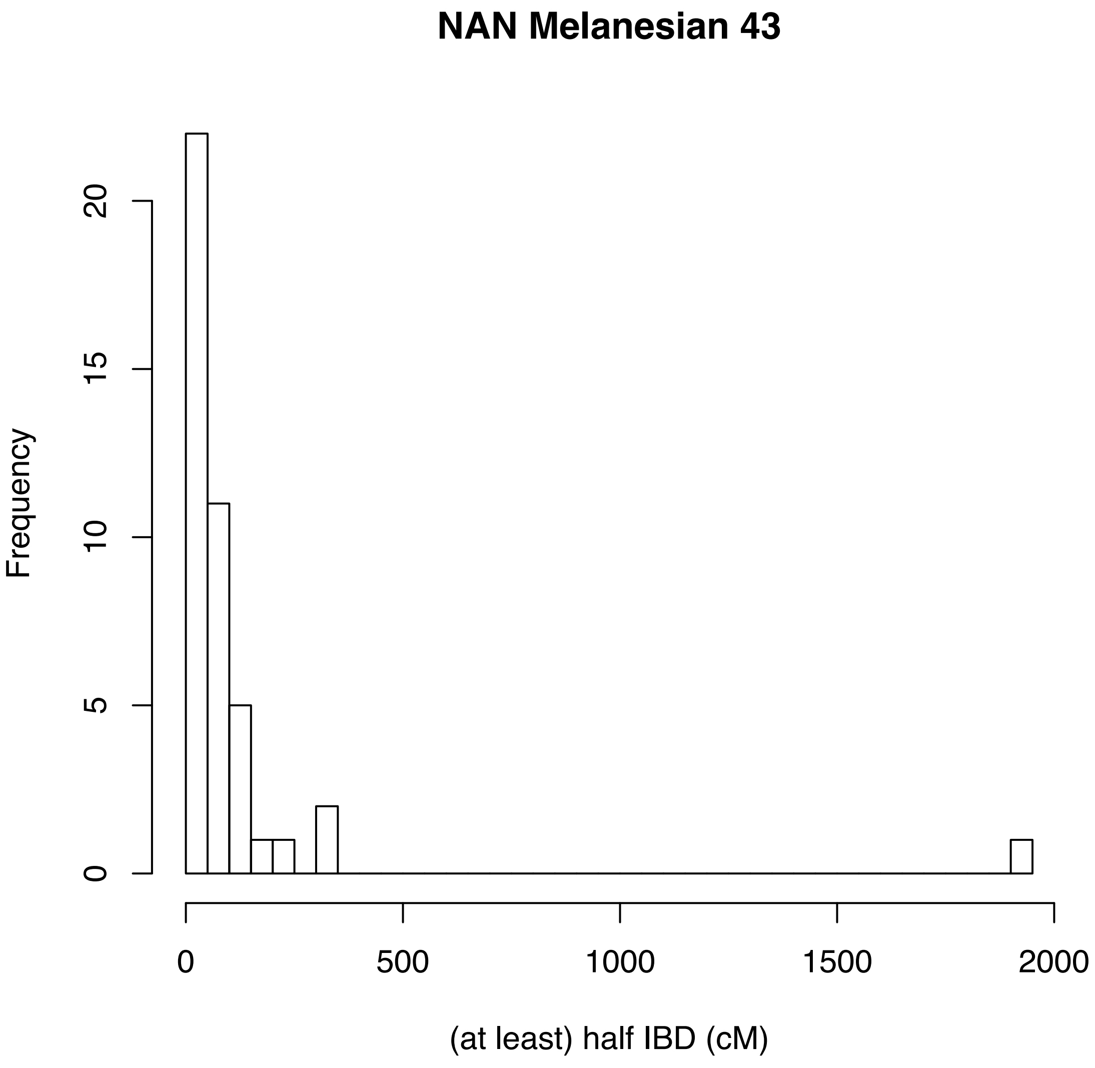
NAN Melanesian 43
Frequency
0
5
10
15
20
0
500
1000
1500
2000
(at least) half IBD (cM)

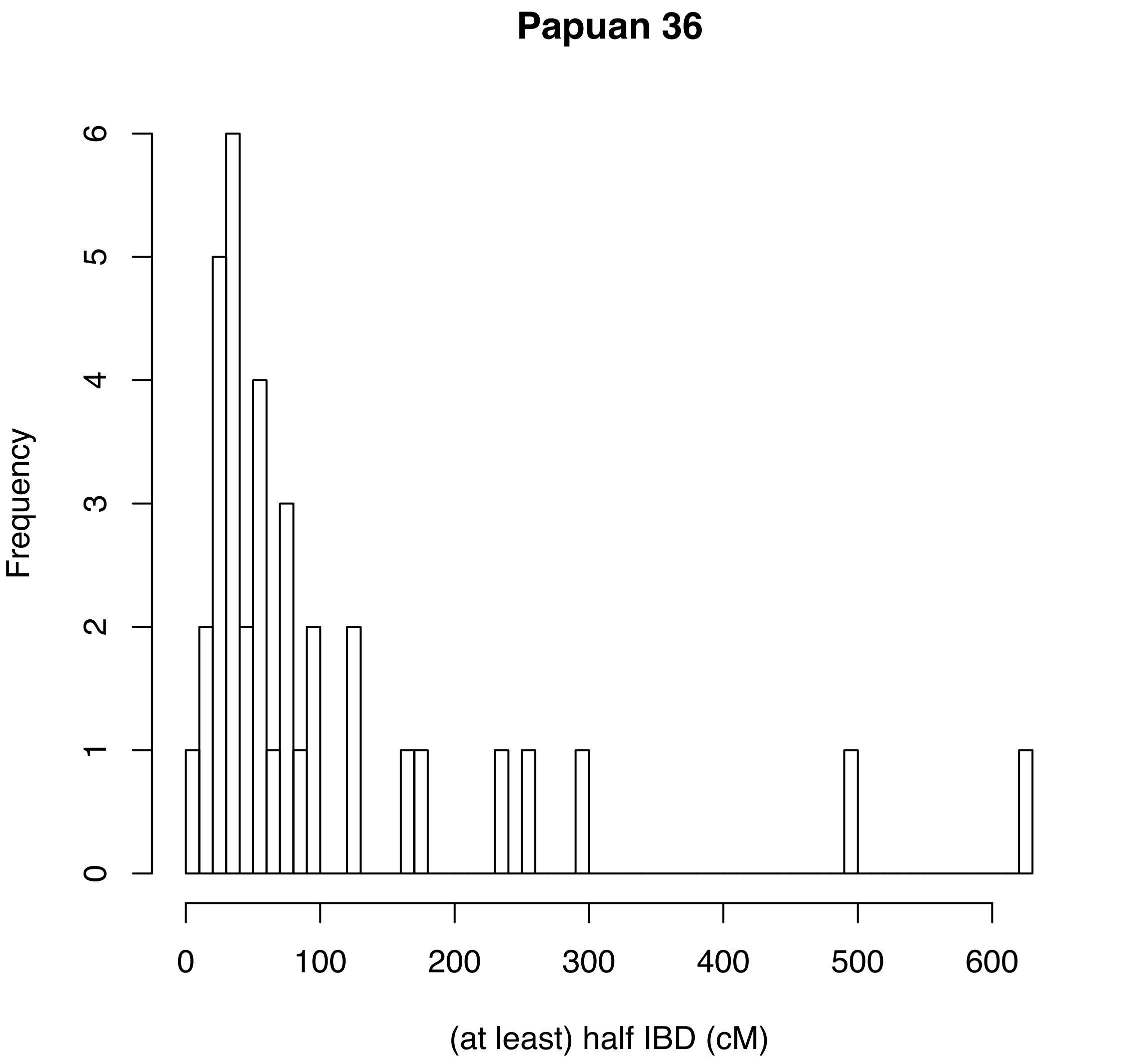

Papuan 36
Frequency
0
1
2
3
4
5
6
0
100
200
300
400
500
600
(at least) half IBD (cM)

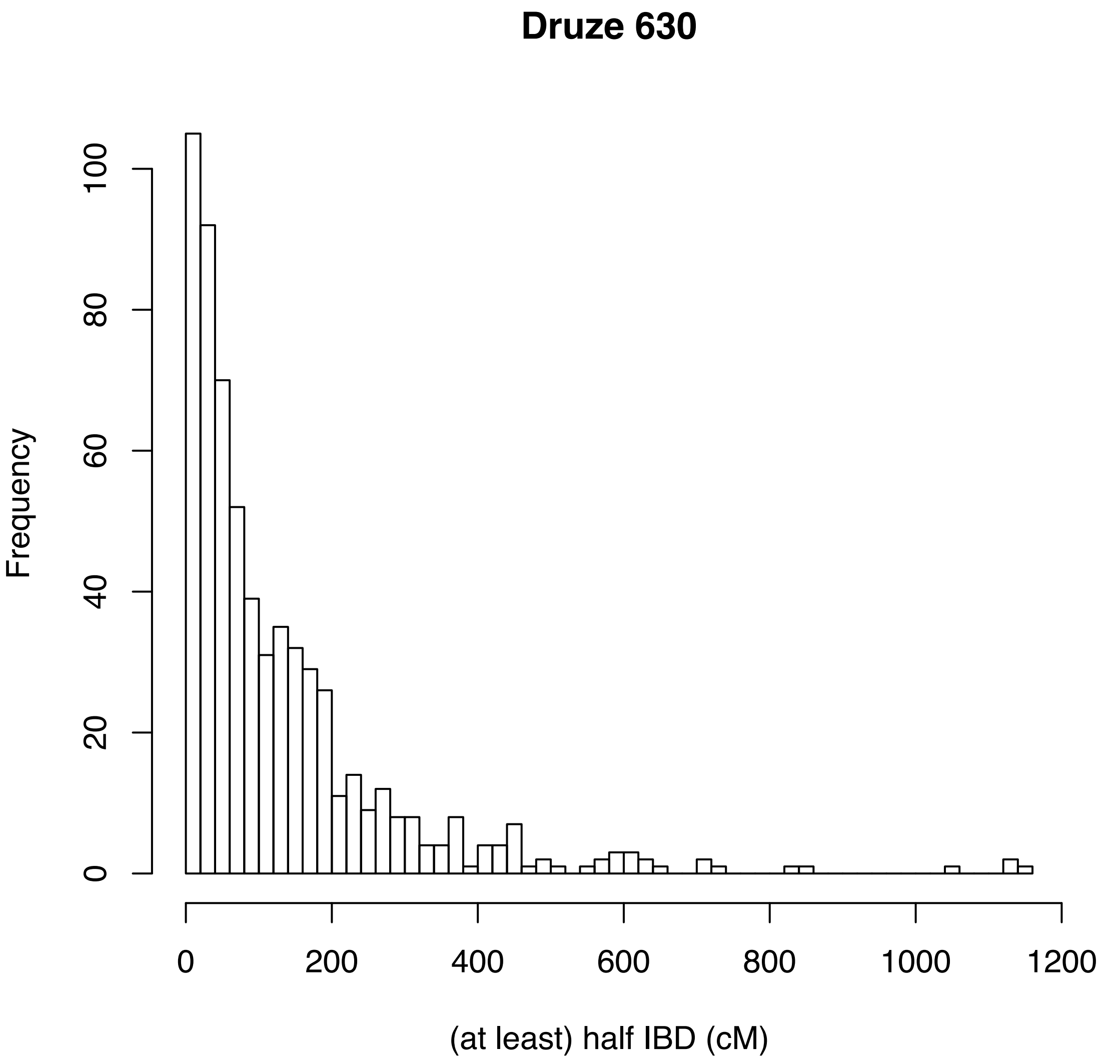
Druze 630
Frequency
0
20
40
60
80
100
0
200
400
600
800
1000
1200
(at least) half IBD (cM)

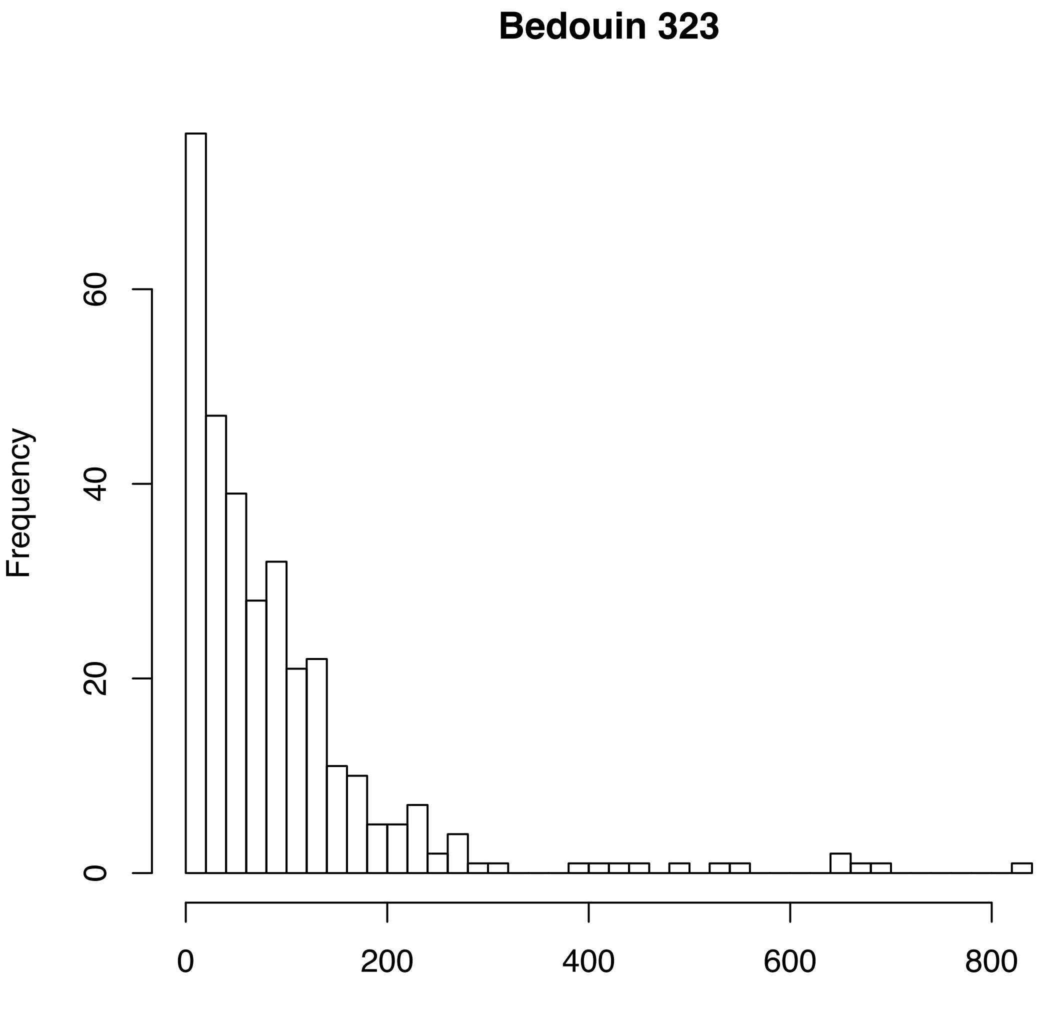
Bedouin 323
Frequency
0
20
40
60
0
200
400
600
800
(at least) half IBD (cM)

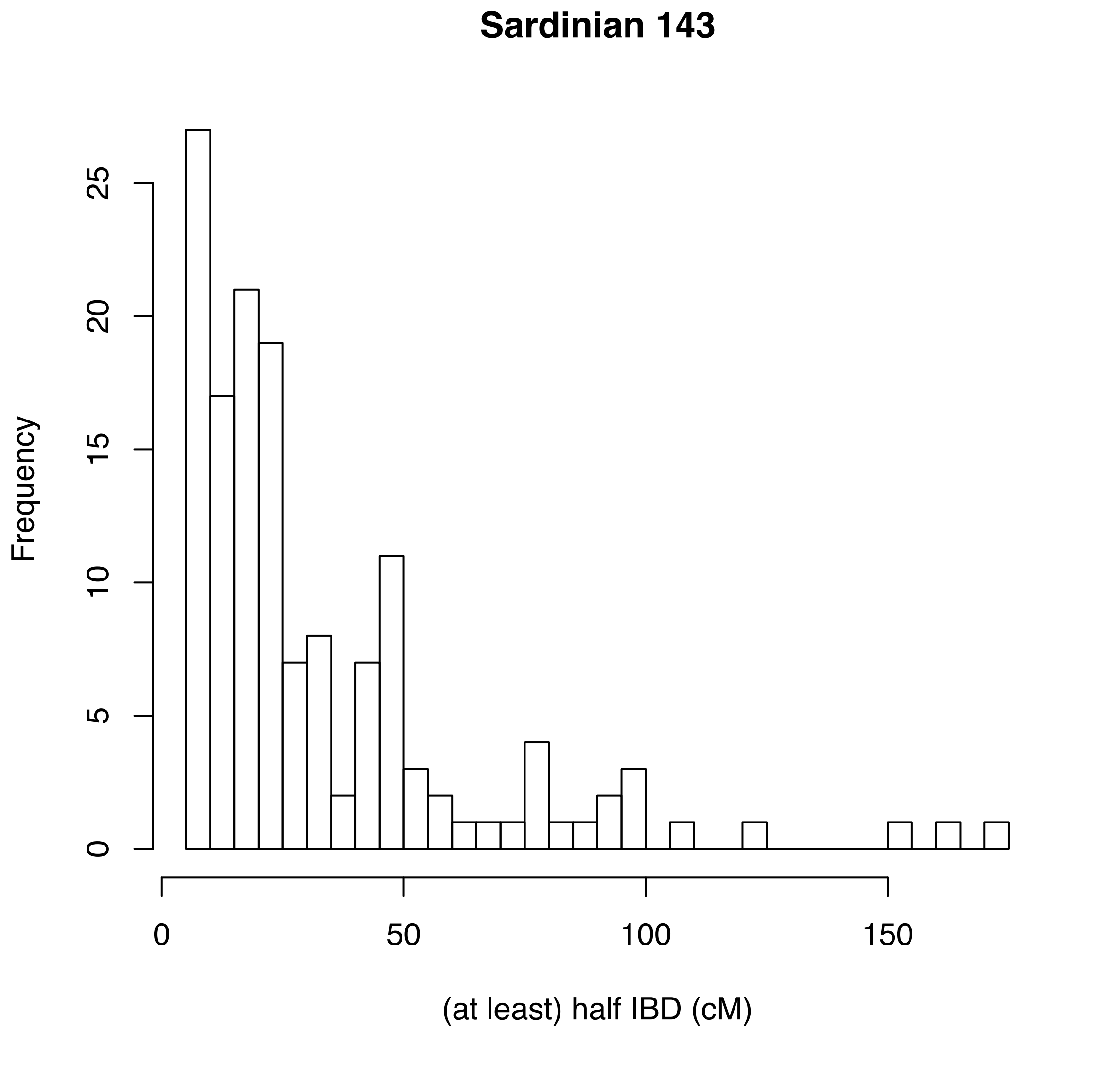

Sardinian 143
Frequency
0
5
10
15
20
25
0
50
100
150
(at least) half IBD (cM)

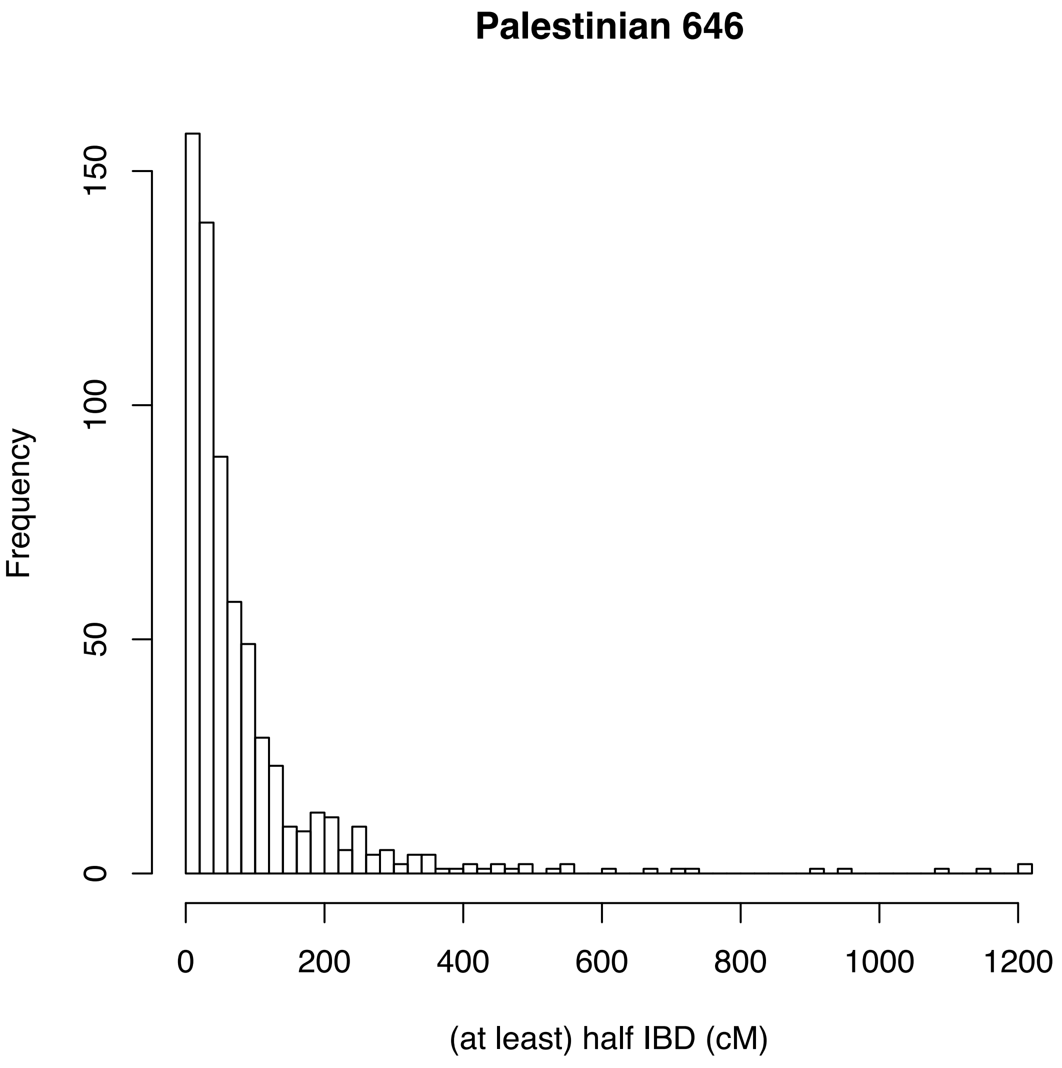
Palestinian 646
Frequency
0
50
100
150
0
200
400
600
800
1000
1200
(at least) half IBD (cM)

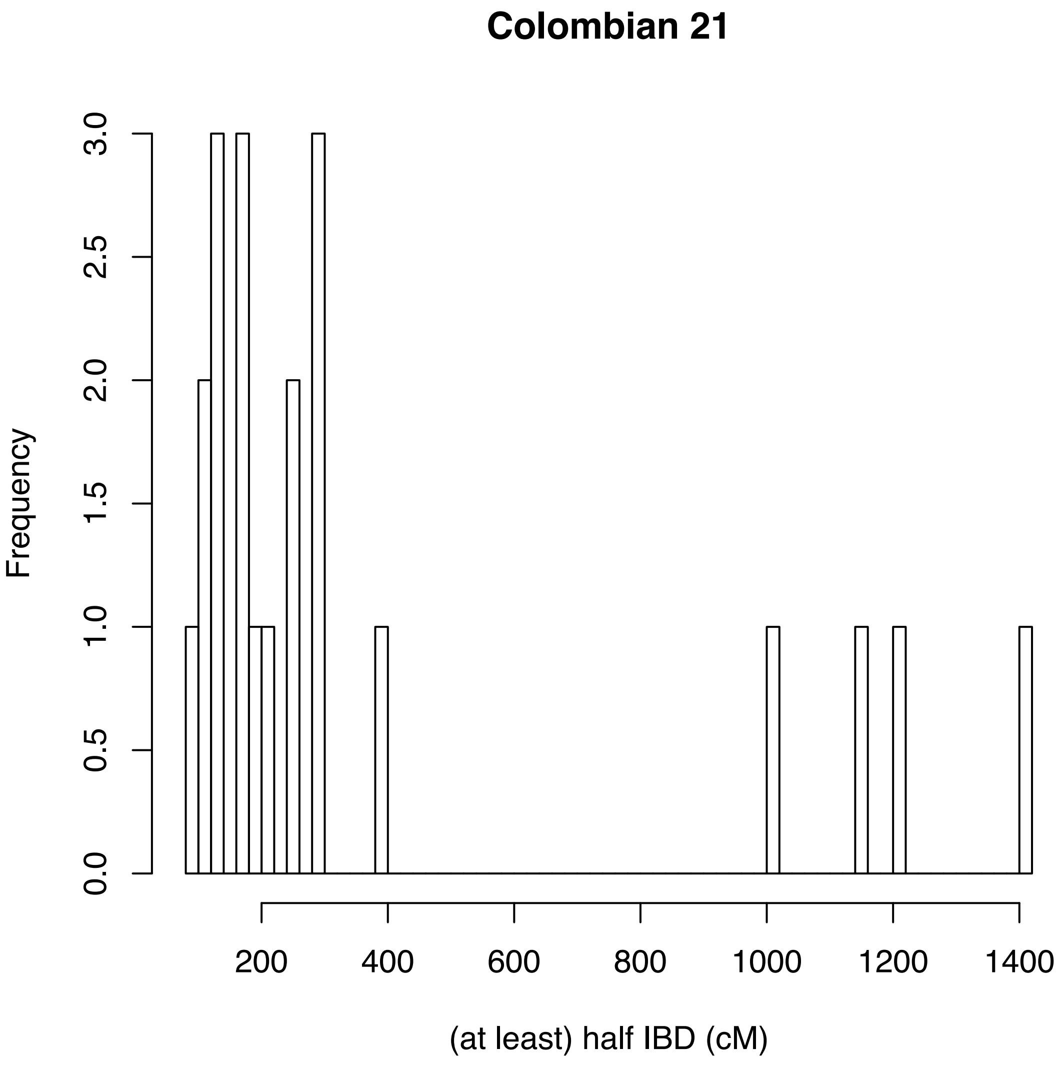
Colombian 21
Frequency
0.0
0.5
1.0
1.5
2.0
2.5
3.0
200
400
600
800
1000
1200
1400
(at least) half IBD (cM)

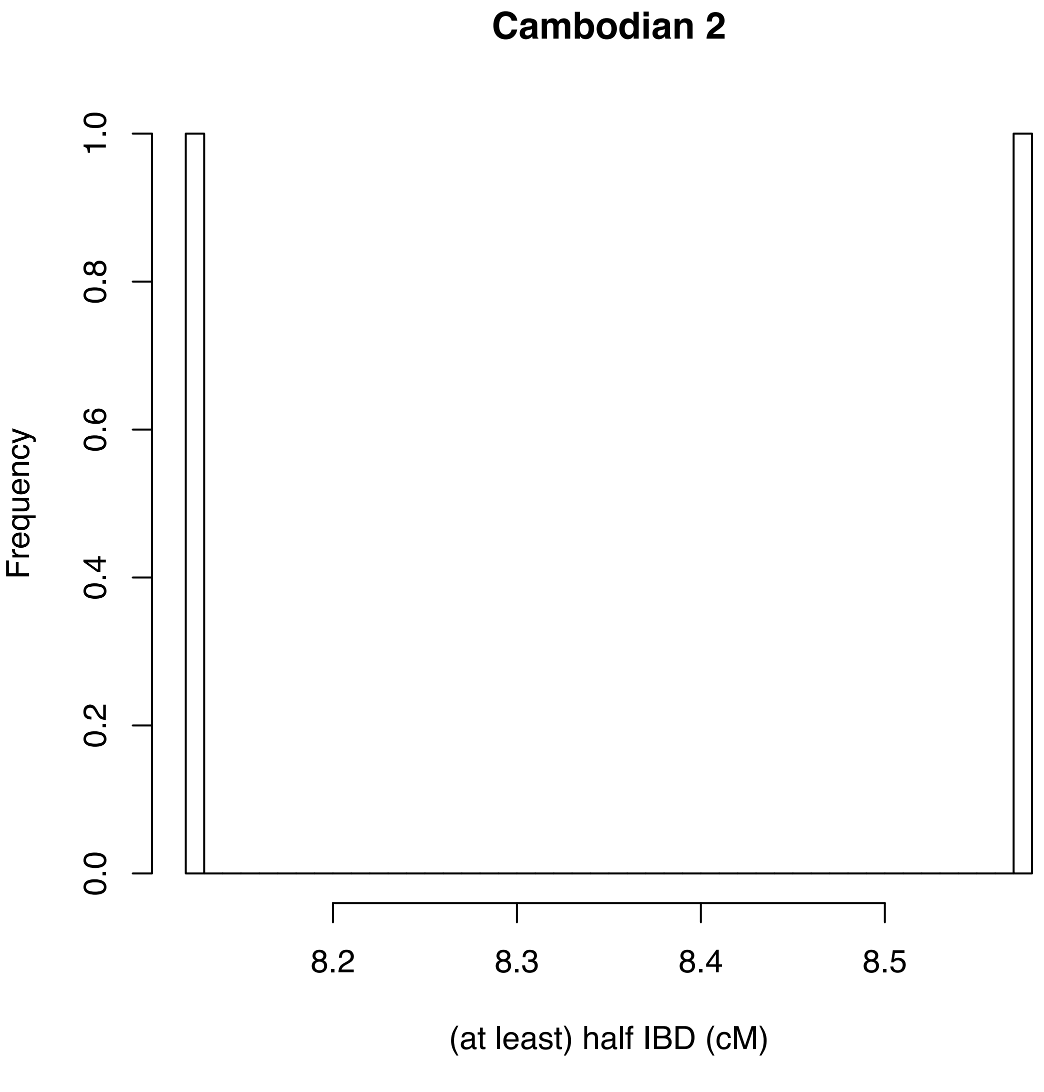
Cambodian 2
Frequency
1.0
0.8
0.6
0.4
0.2
0.0
8.2
8.3
8.4
8.5
(at least) half IBD (cM)

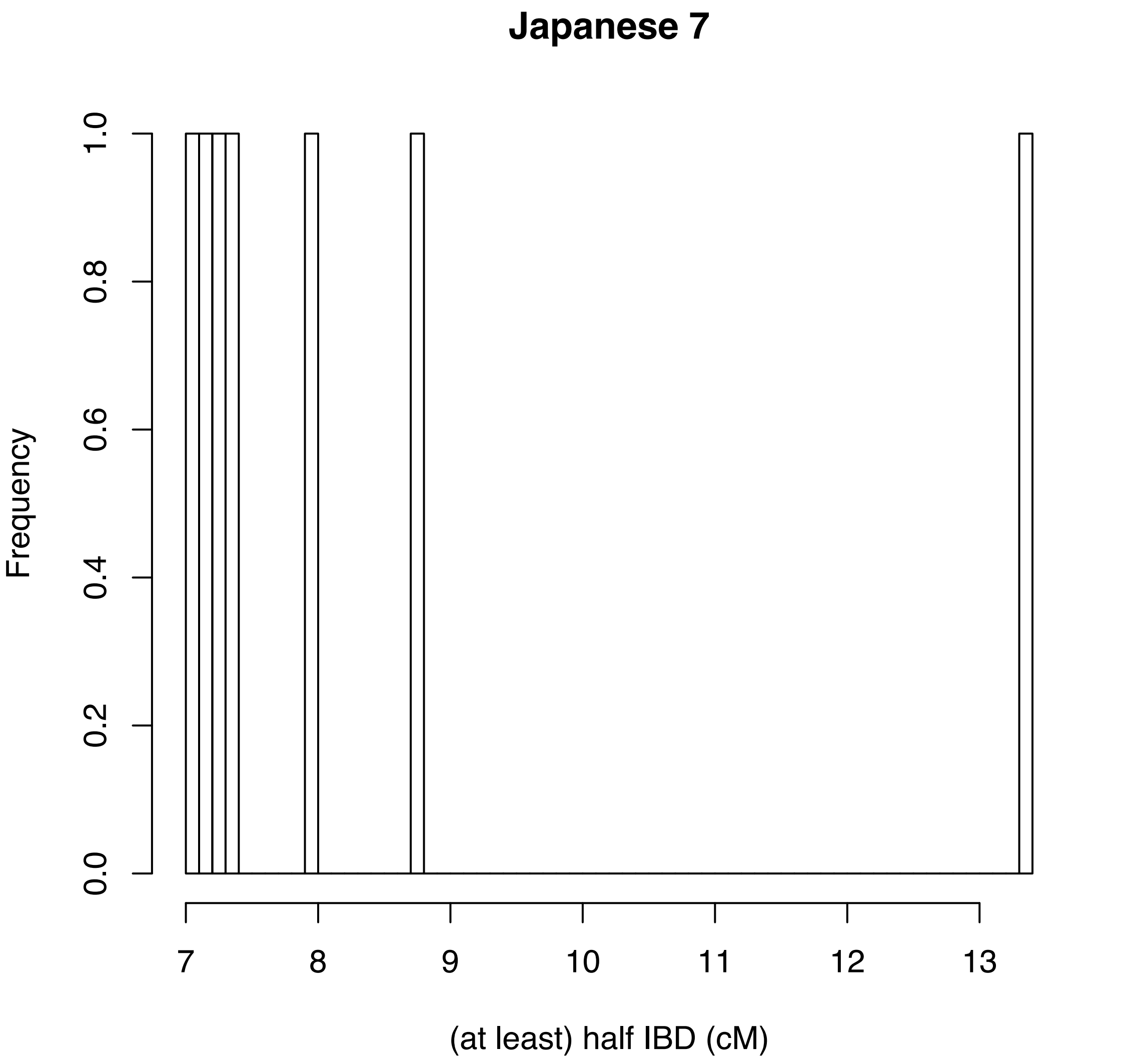
Japanese 7
Frequency
0.0
0.2
0.4
0.6
0.8
1.0
7
8
9
10
11
12
13
(at least) half IBD (cM)

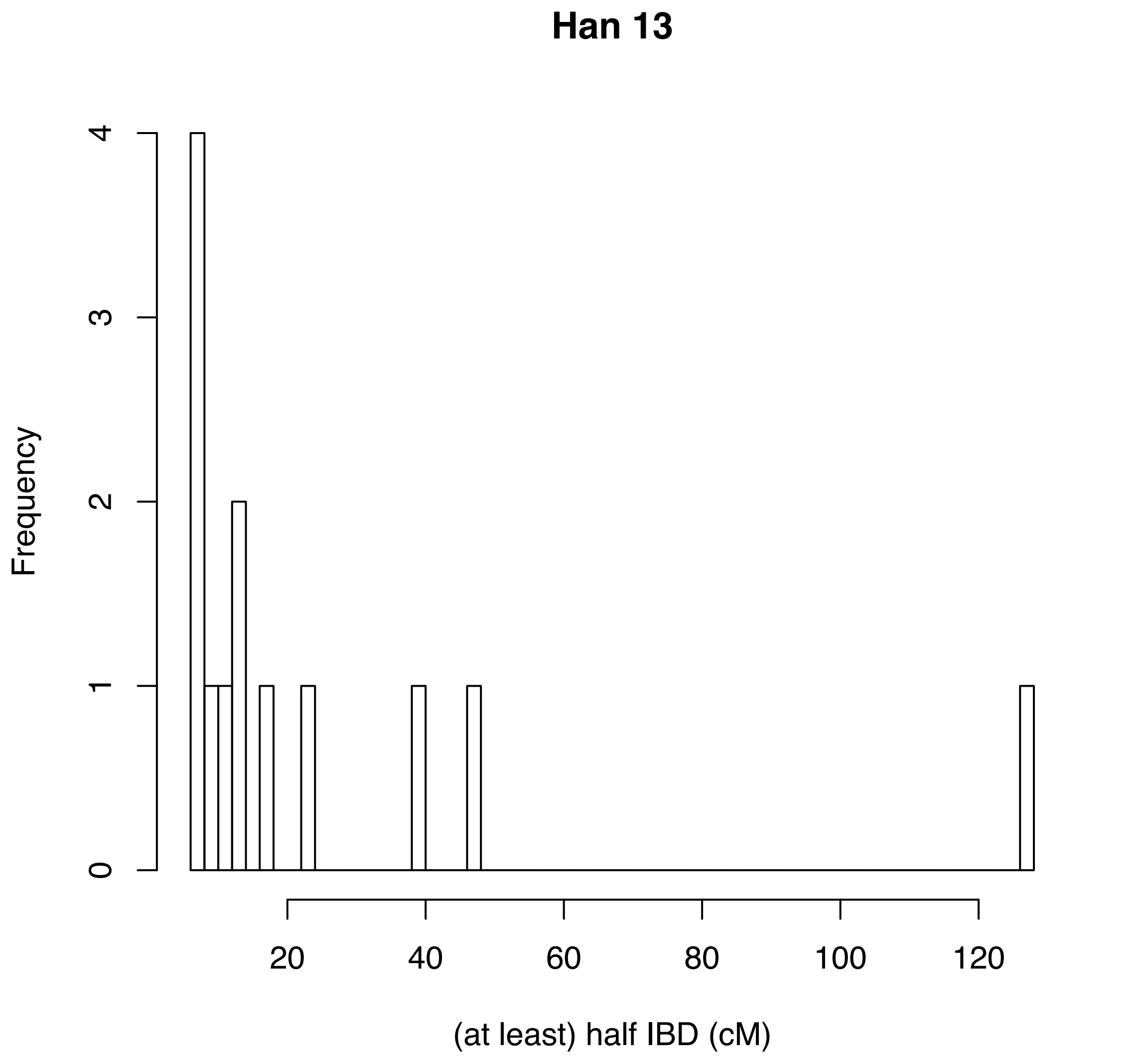

Han 13
Frequency
0
1
2
3
4
20
40
60
80
100
120
(at least) half IBD (cM)

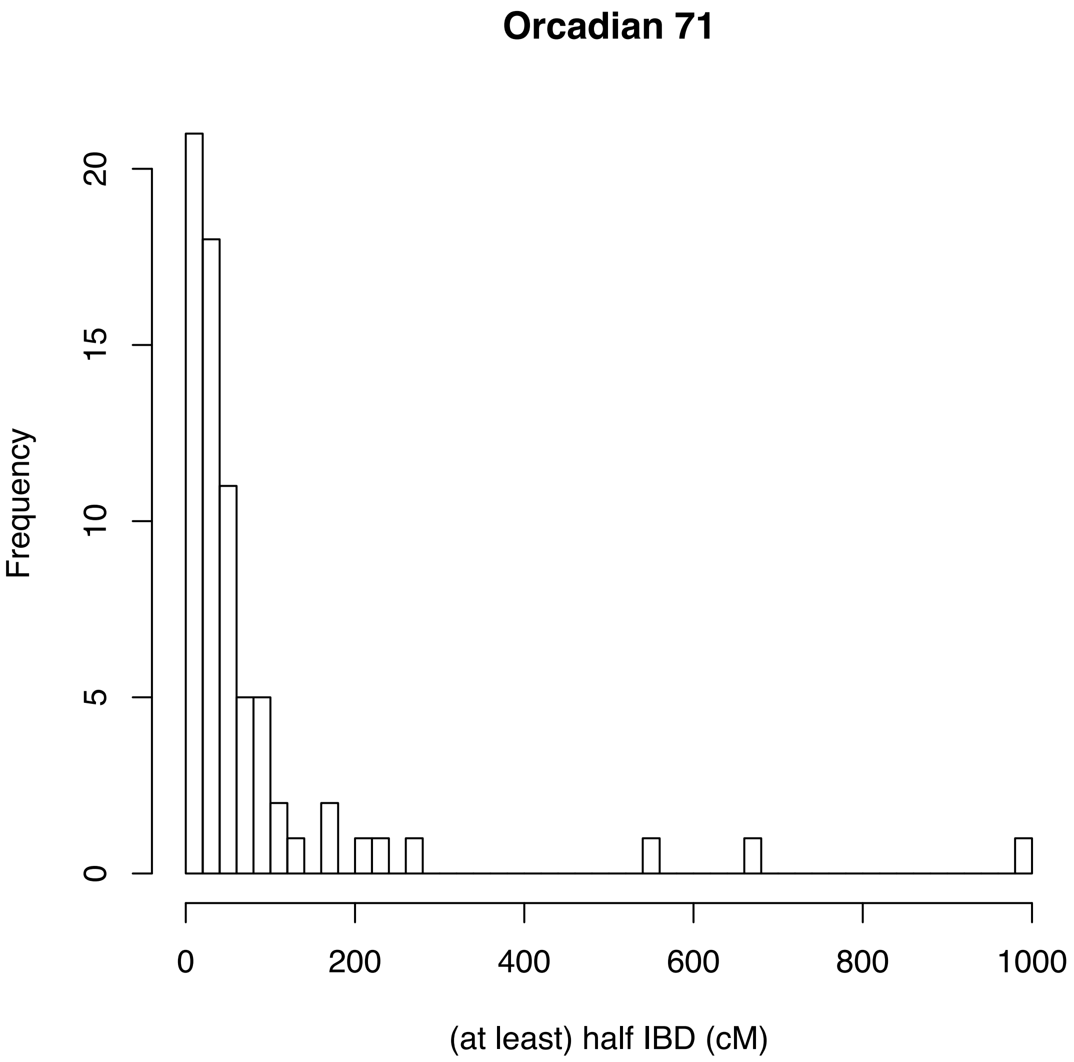
Orcadian 71
Frequency
0
5
10
15
20
0
200
400
600
800
1000
(at least) half IBD (cM)

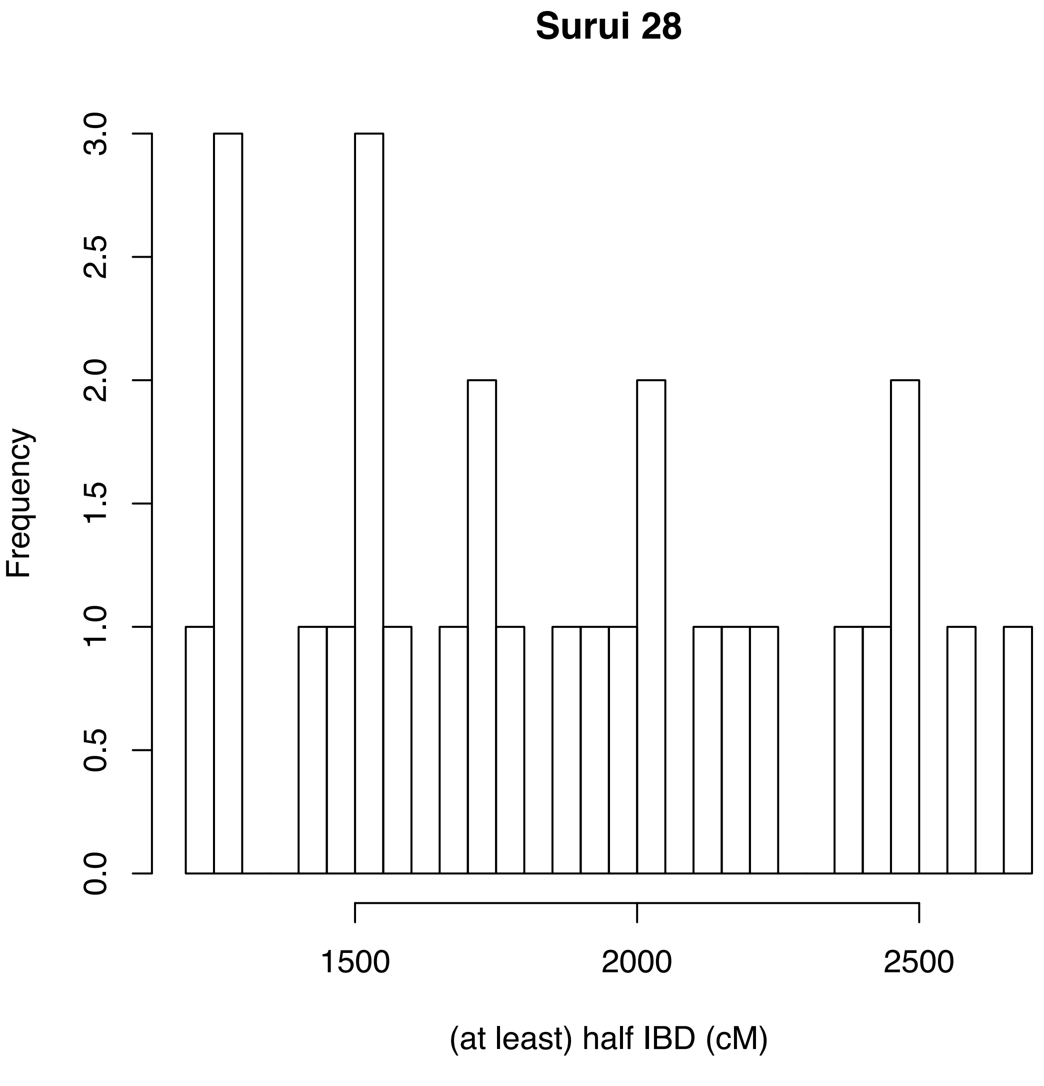

Surui 28
Frequency
0.0
0.5
1.0
1.5
2.0
2.5
3.0
1500
2000
2500
(at least) half IBD (cM)

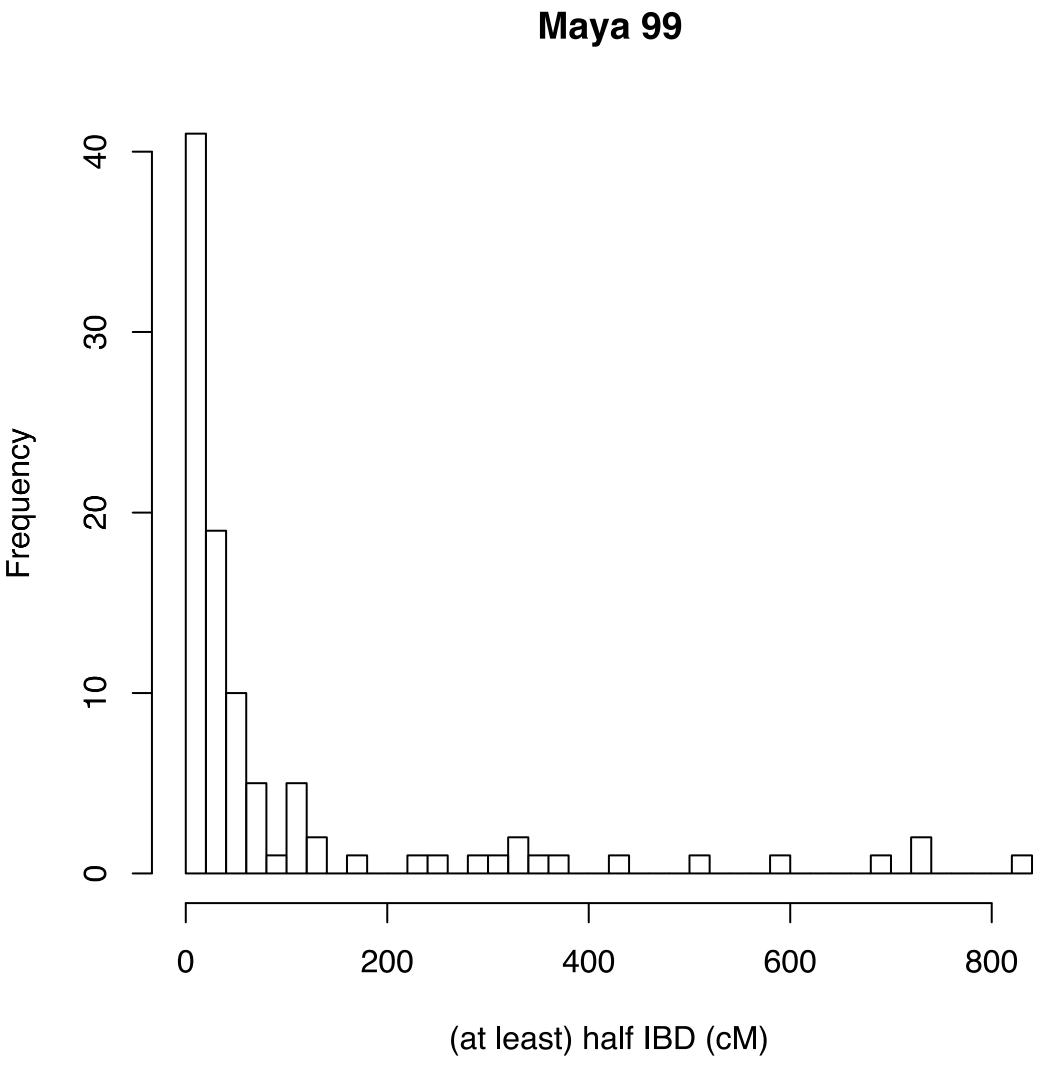

Maya 99
Frequency
0
10
20
30
40
0
200
400
600
800
(at least) half IBD (cM)

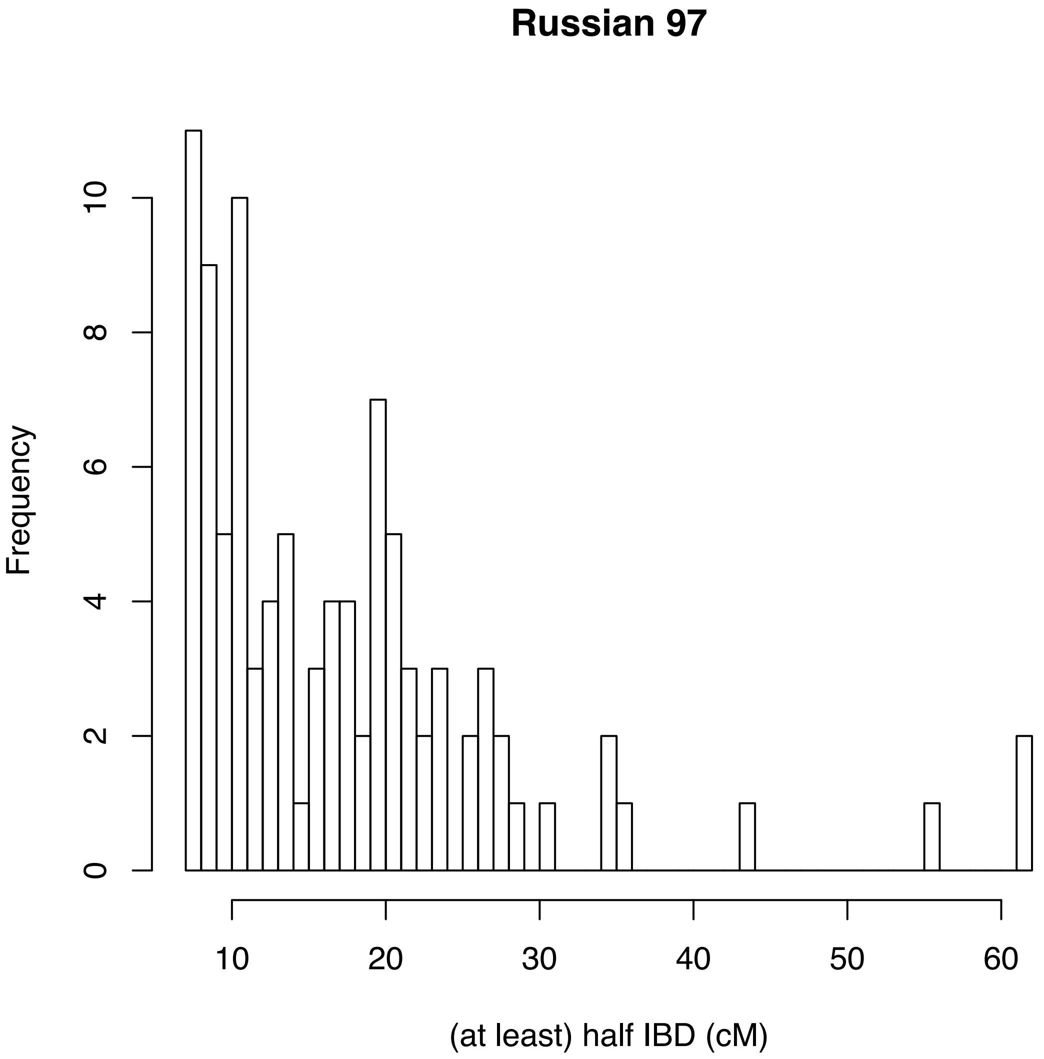

Russian 97
Frequency
0
2
4
6
8
10
10
20
30
40
50
60
(at least) half IBD (cM)

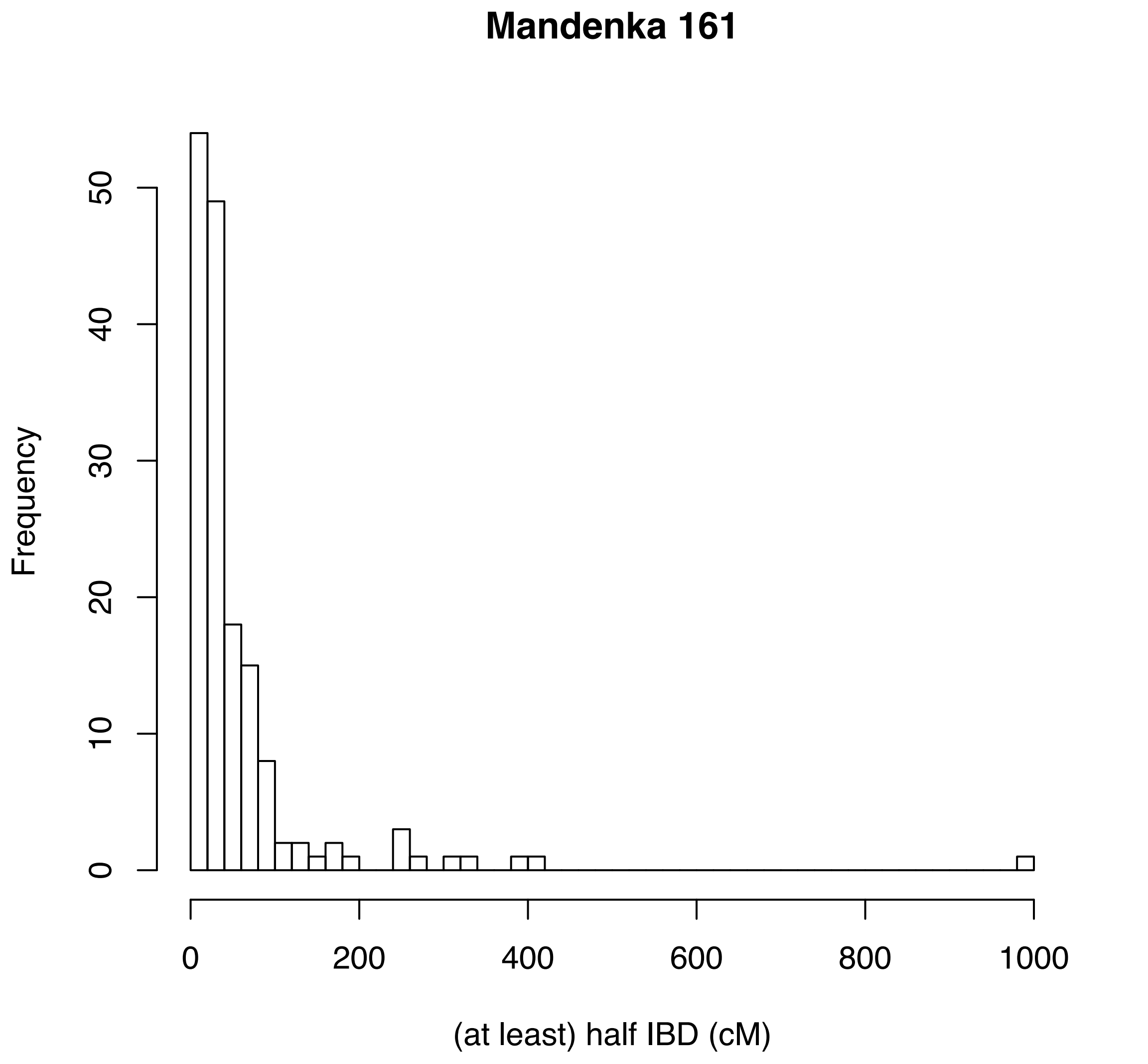

Mandenka 161
Frequency
0
10
20
30
40
50
0
200
400
600
800
1000
(at least) half IBD (cM)

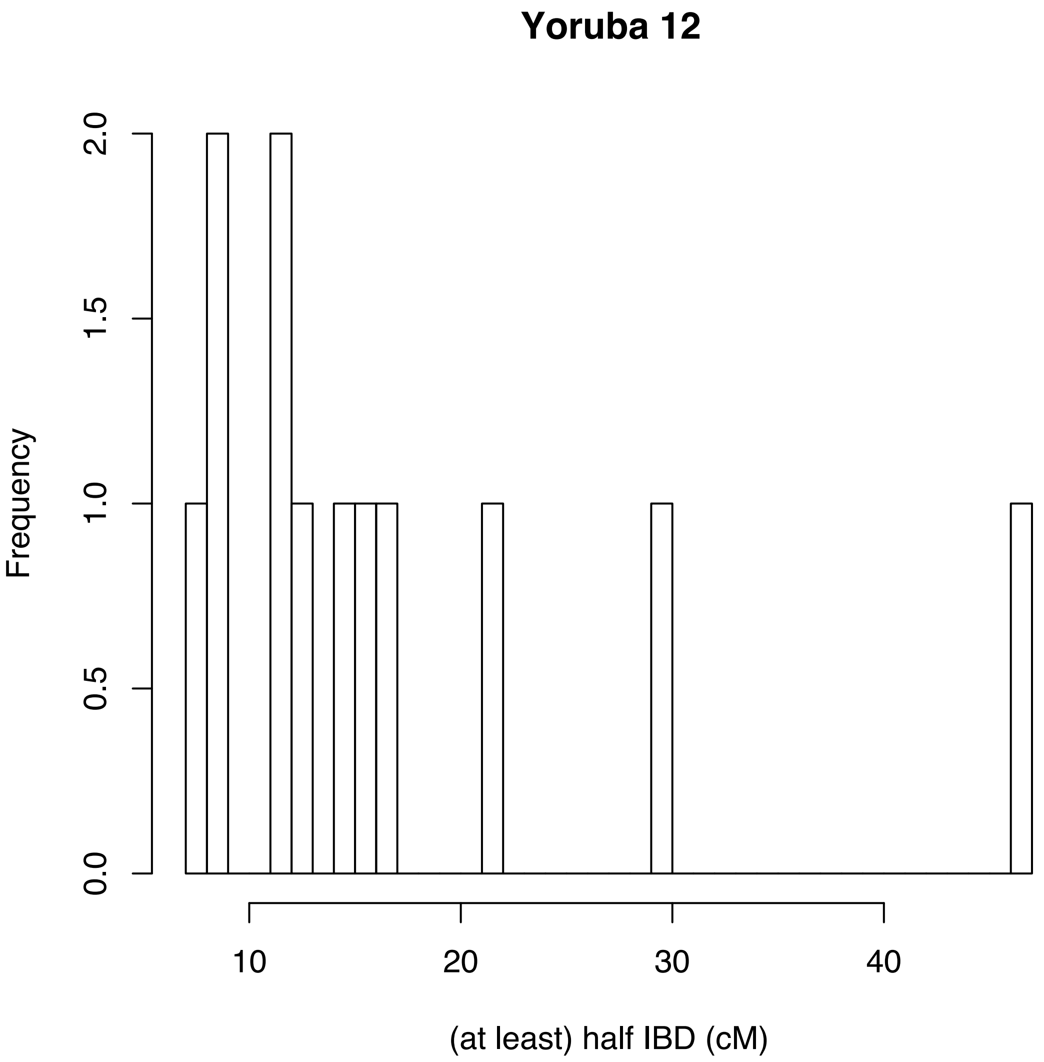
Yoruba 12
Frequency
0.0
0.5
1.0
1.5
2.0
10
20
30
40
(at least) half IBD (cM)

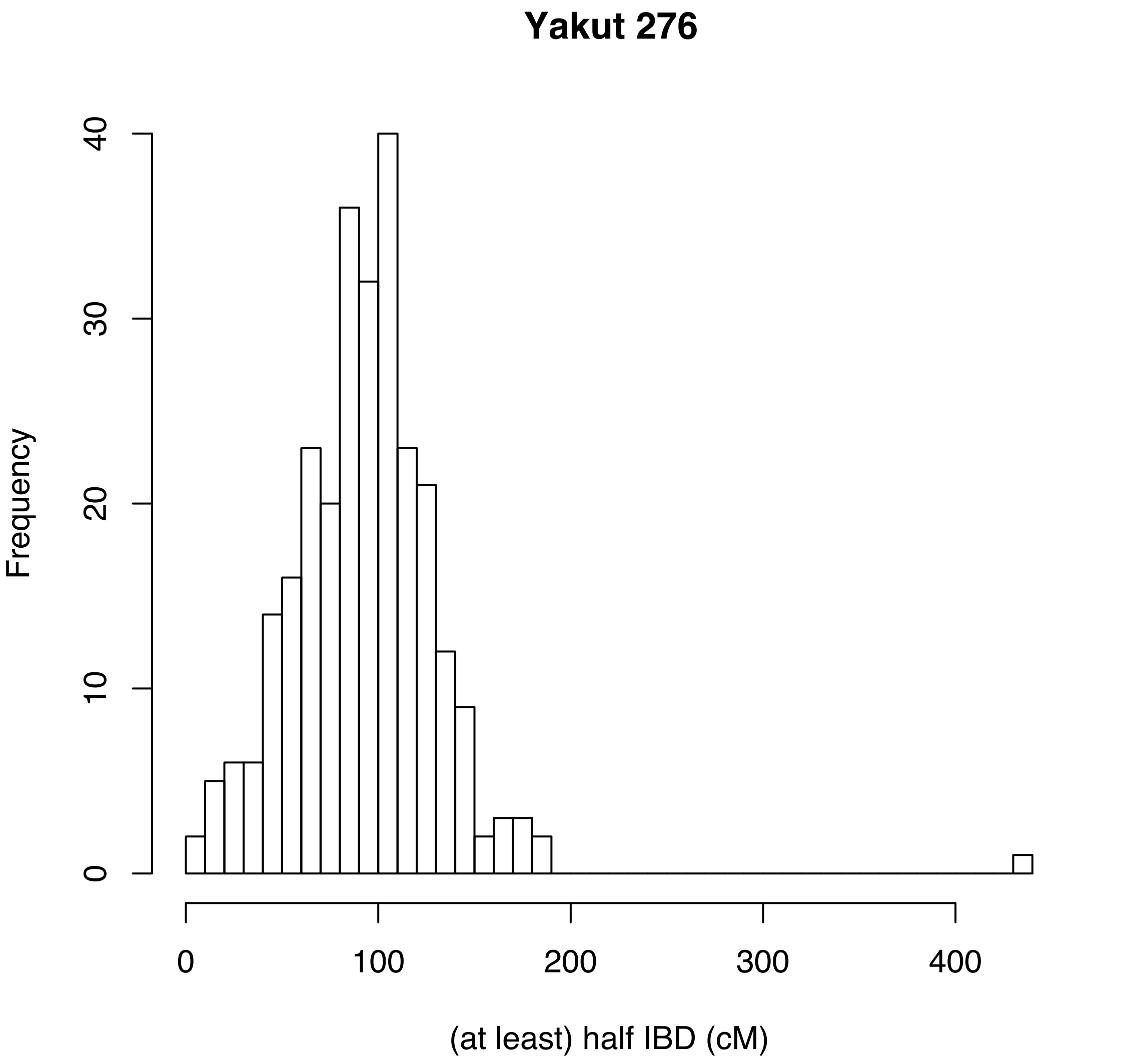

Yakut 276
Frequency
0
10
20
30
40
0
100
200
300
400
(at least) half IBD (cM)

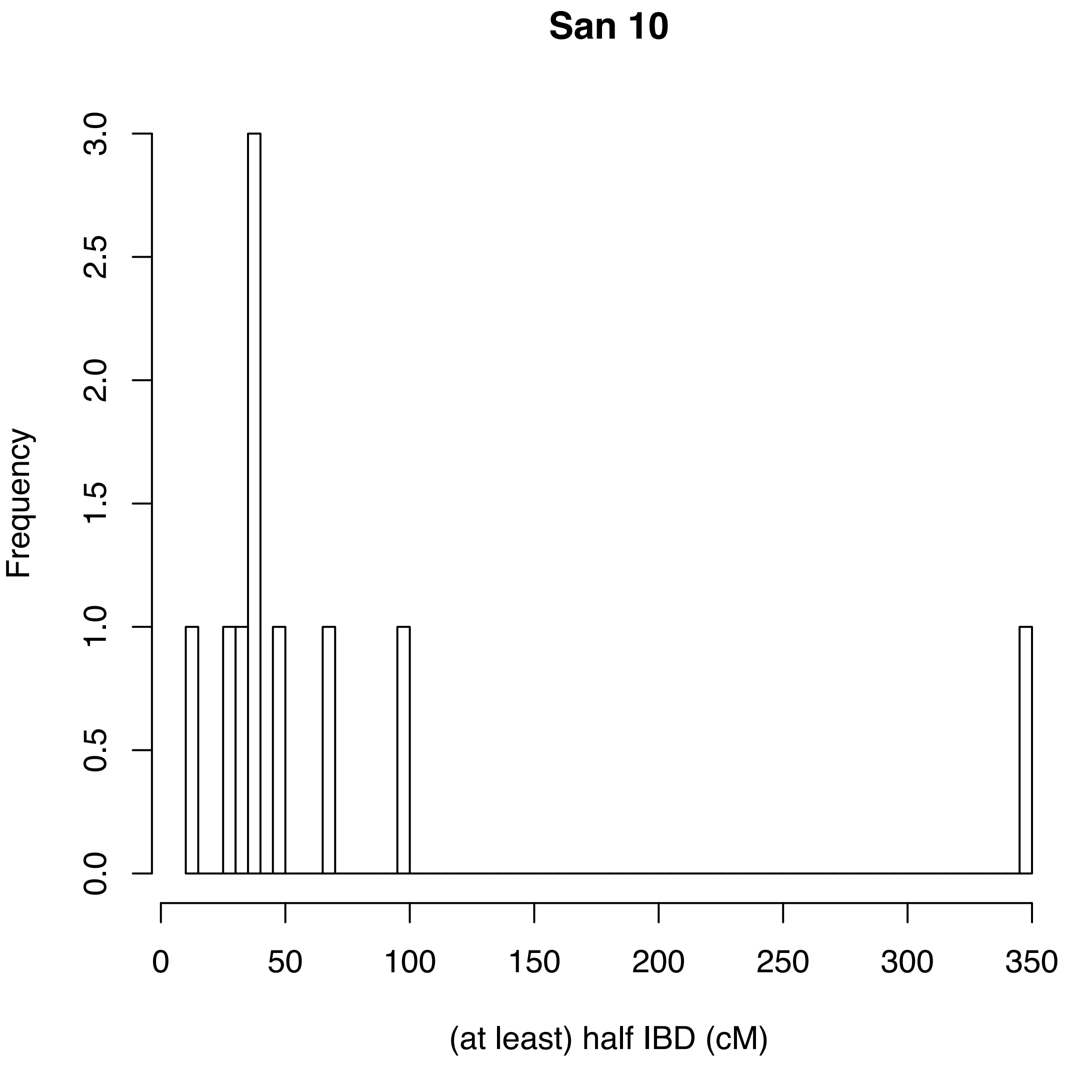
San 10
Frequency
0.0
0.5
1.0
1.5
2.0
2.5
3.0
0
50
100
150
200
250
300
350
(at least) half IBD (cM)

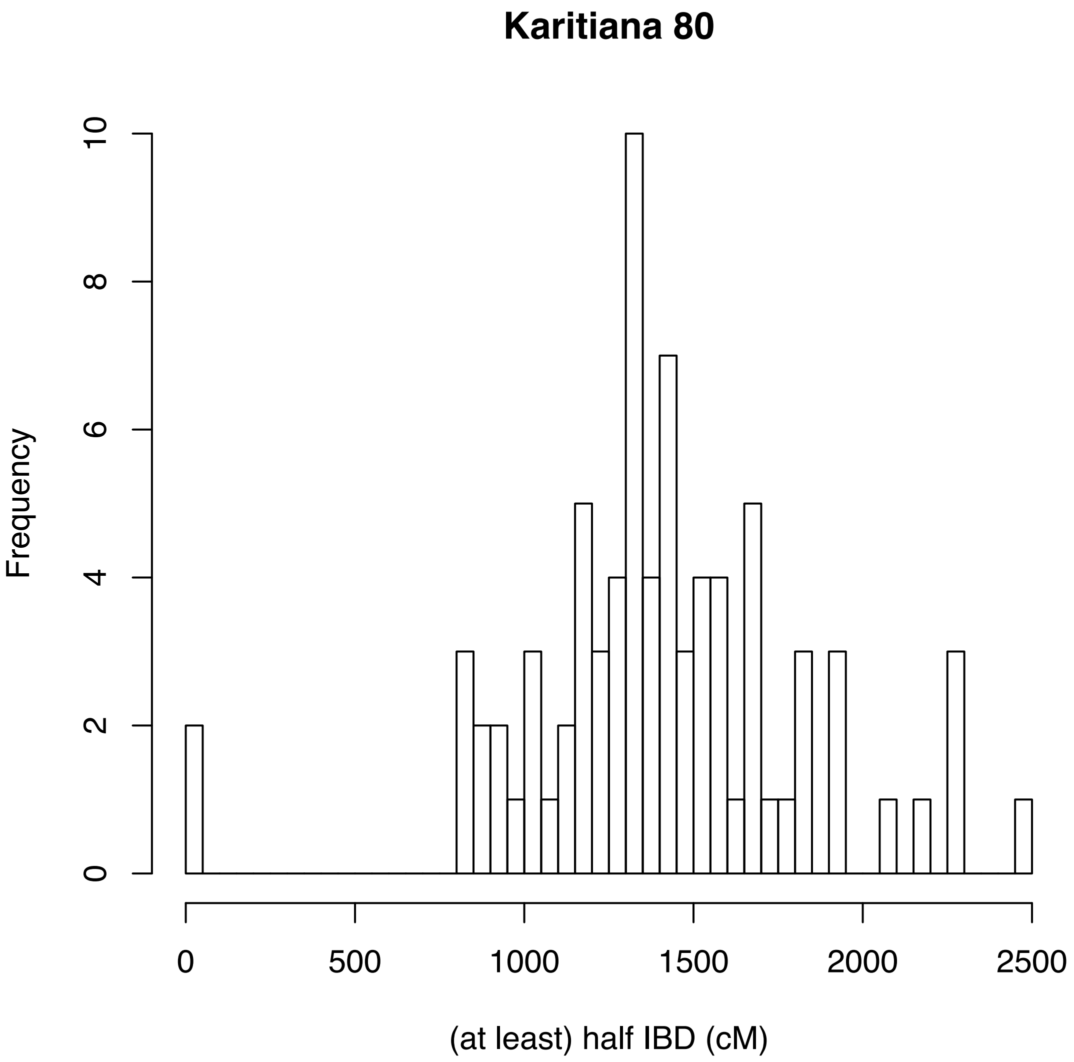

Karitiana 80
Frequency
0
2
4
6
8
10
0
500
1000
1500
2000
2500
(at least) half IBD (cM)

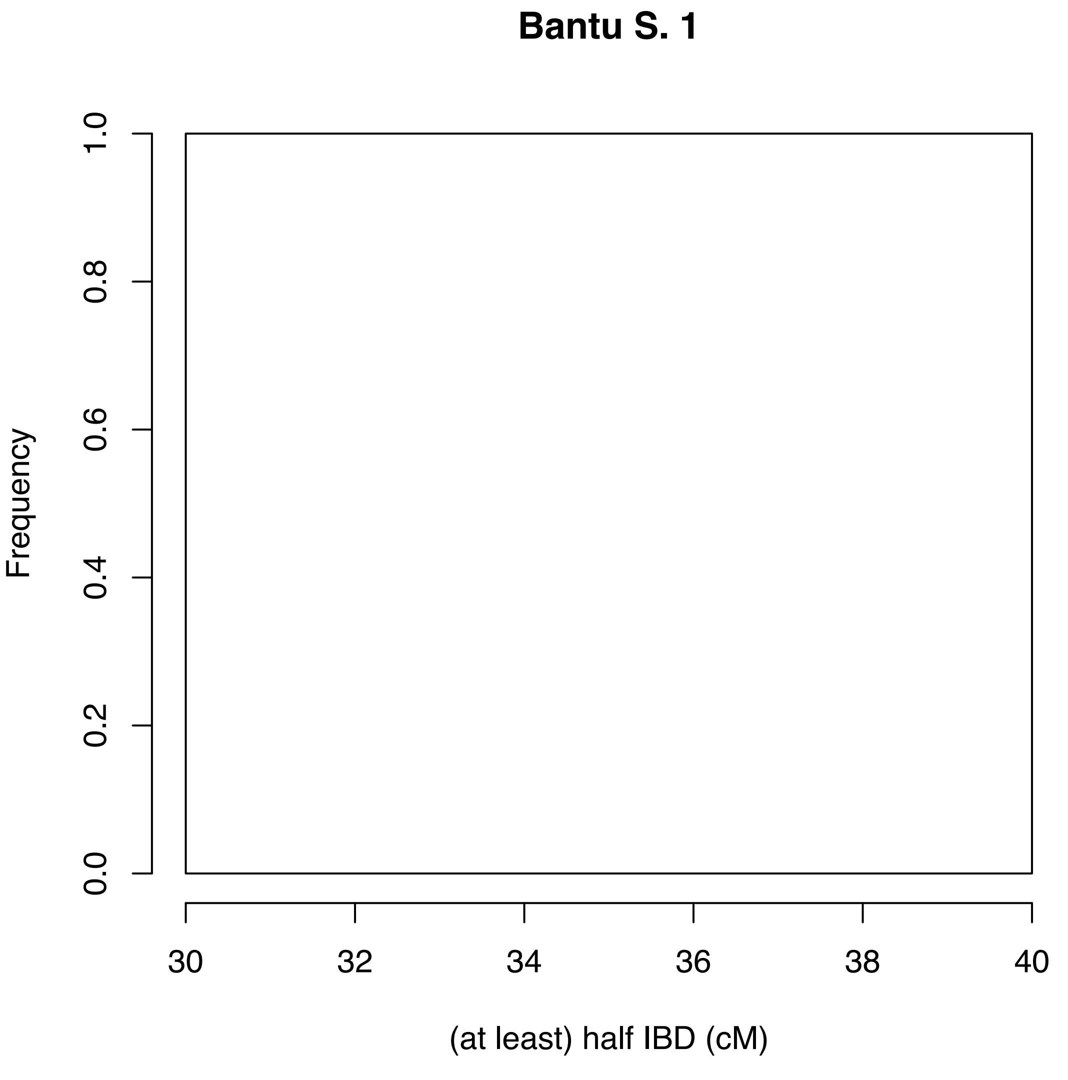
Bantu S. 1
Frequency
0.0
0.2
0.4
0.6
0.8
1.0
30
32
34
36
38
40
(at least) half IBD (cM)

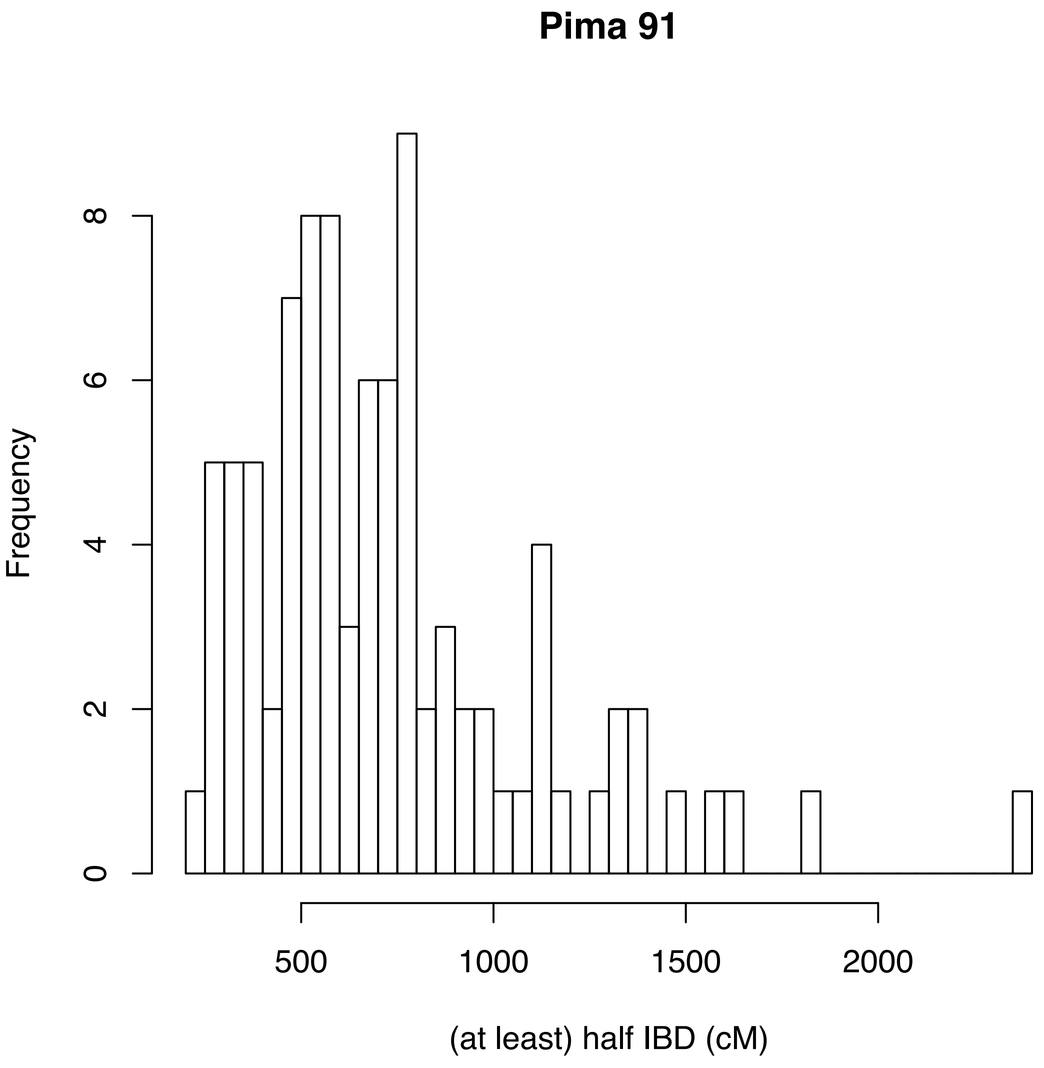

Pima 91
Frequency
0
2
4
6
8
500
1000
1500
2000
(at least) half IBD (cM)

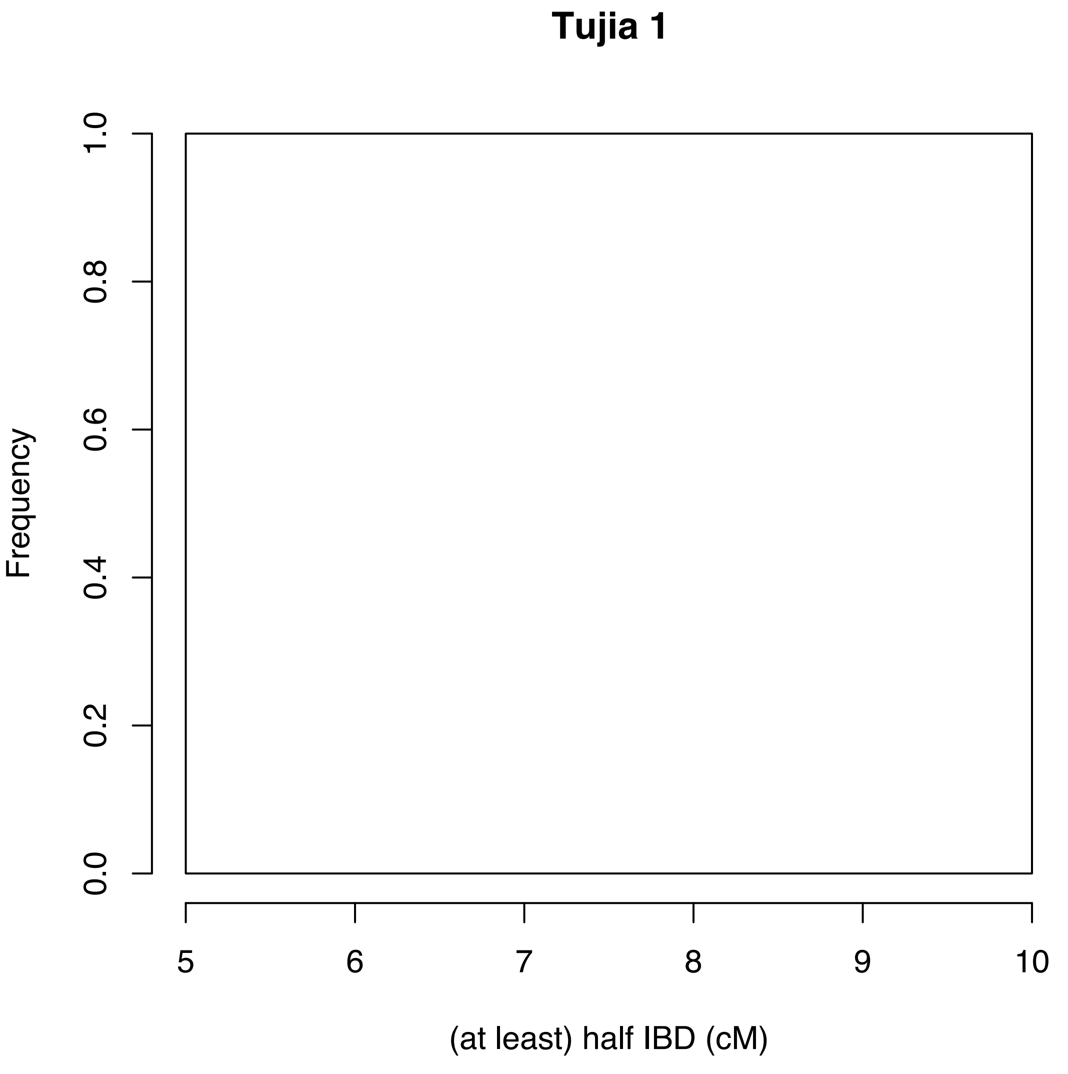
Tujia 1
Frequency
1.0
0.8
0.6
0.4
0.2
0.0
5
6
7
8
9
10
(at least) half IBD (cM)

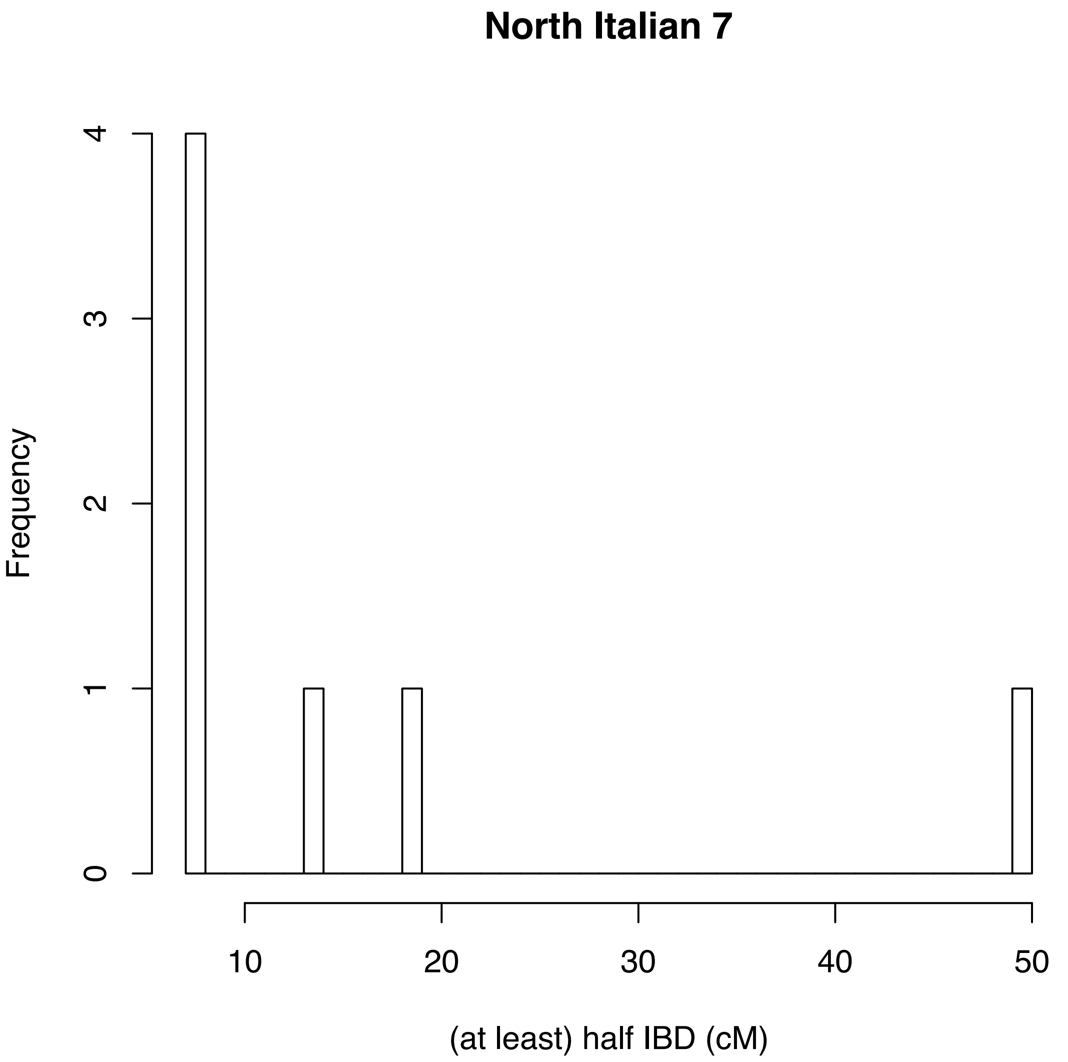
North Italian 7
Frequency
0
1
2
3
4
10
20
30
40
50
(at least) half IBD (cM)

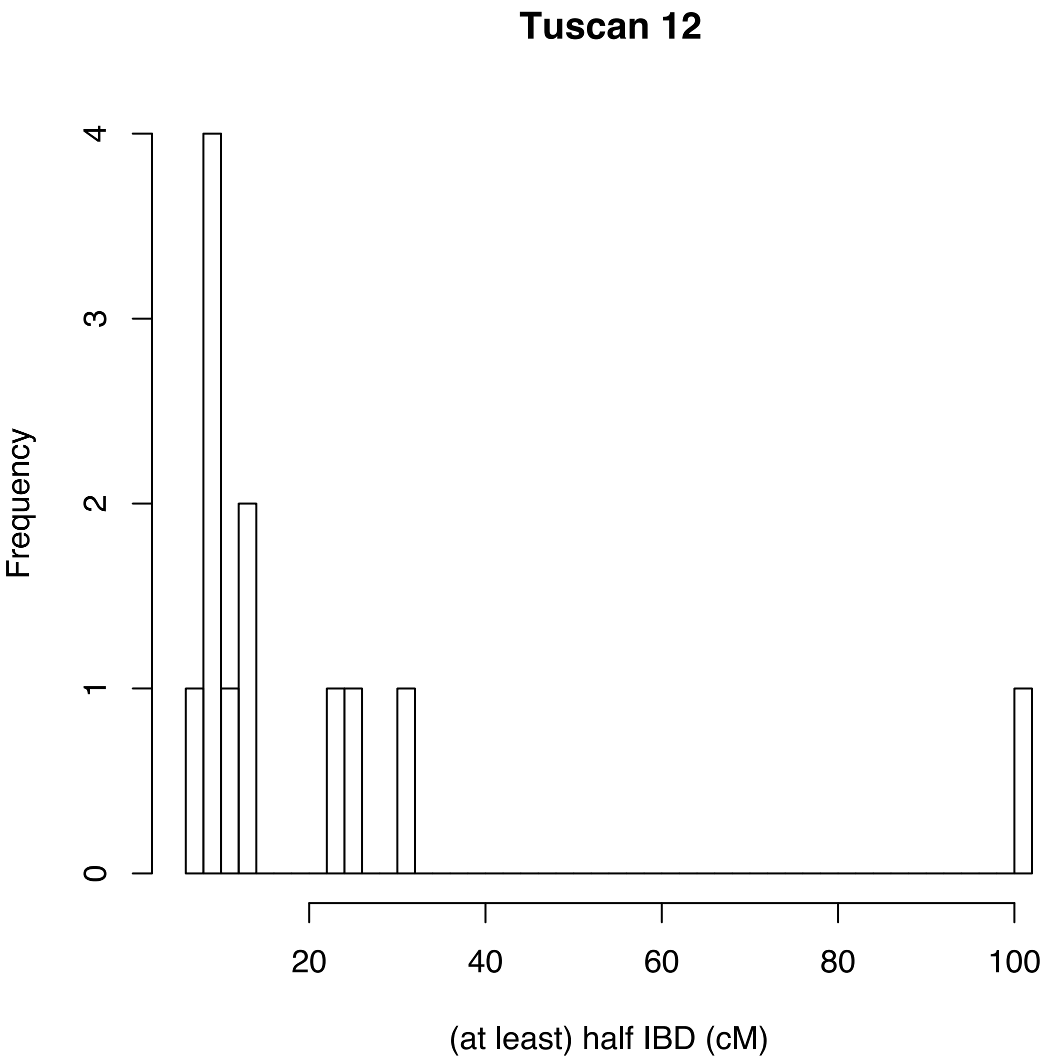

Tuscan 12
Frequency
0
1
2
3
4
20
40
60
80
100
(at least) half IBD (cM)

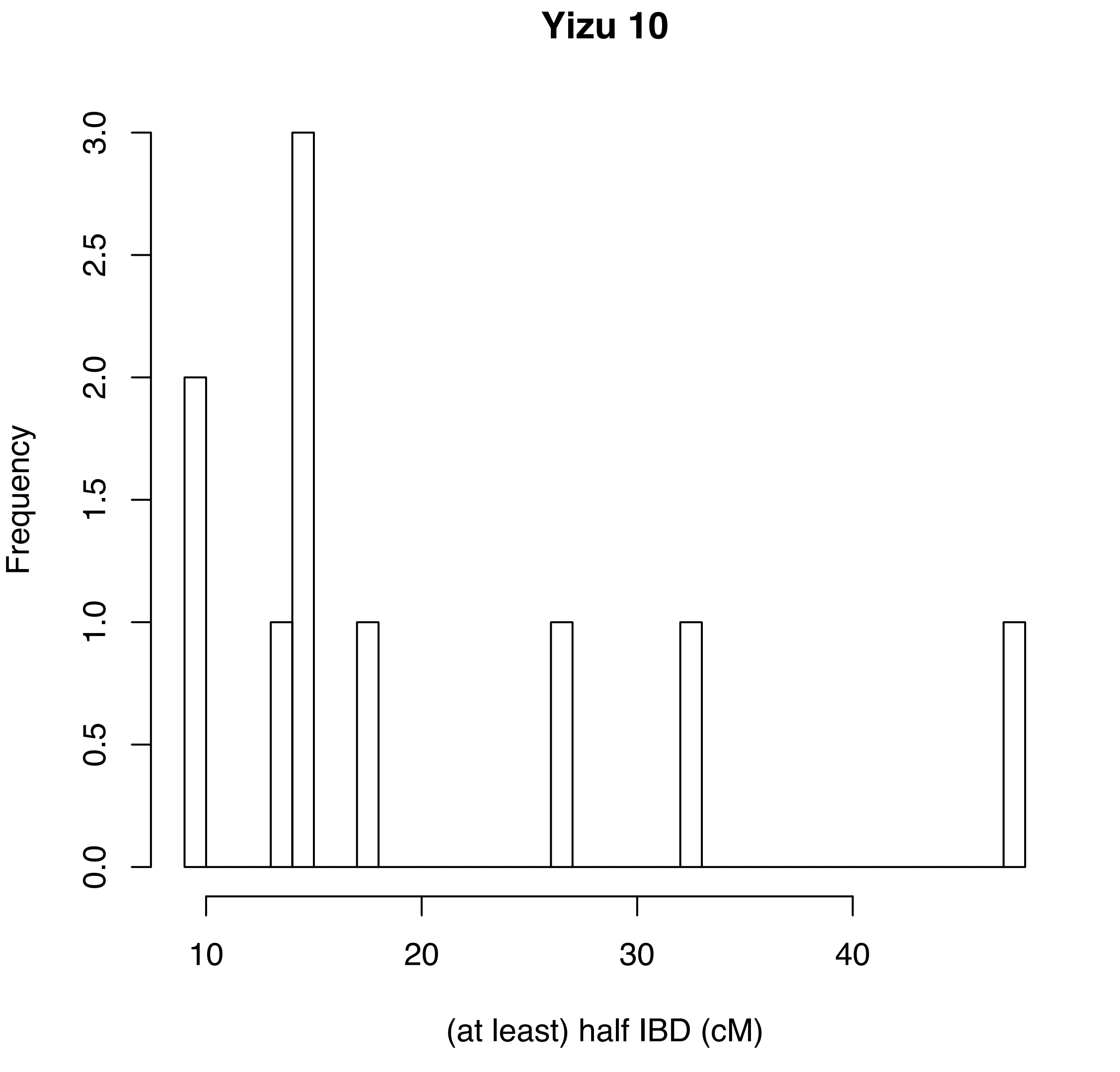

Yizu 10
Frequency
3.0
2.5
2.0
1.5
1.0
0.5
0.0
10
20
30
40
(at least) half IBD (cM)

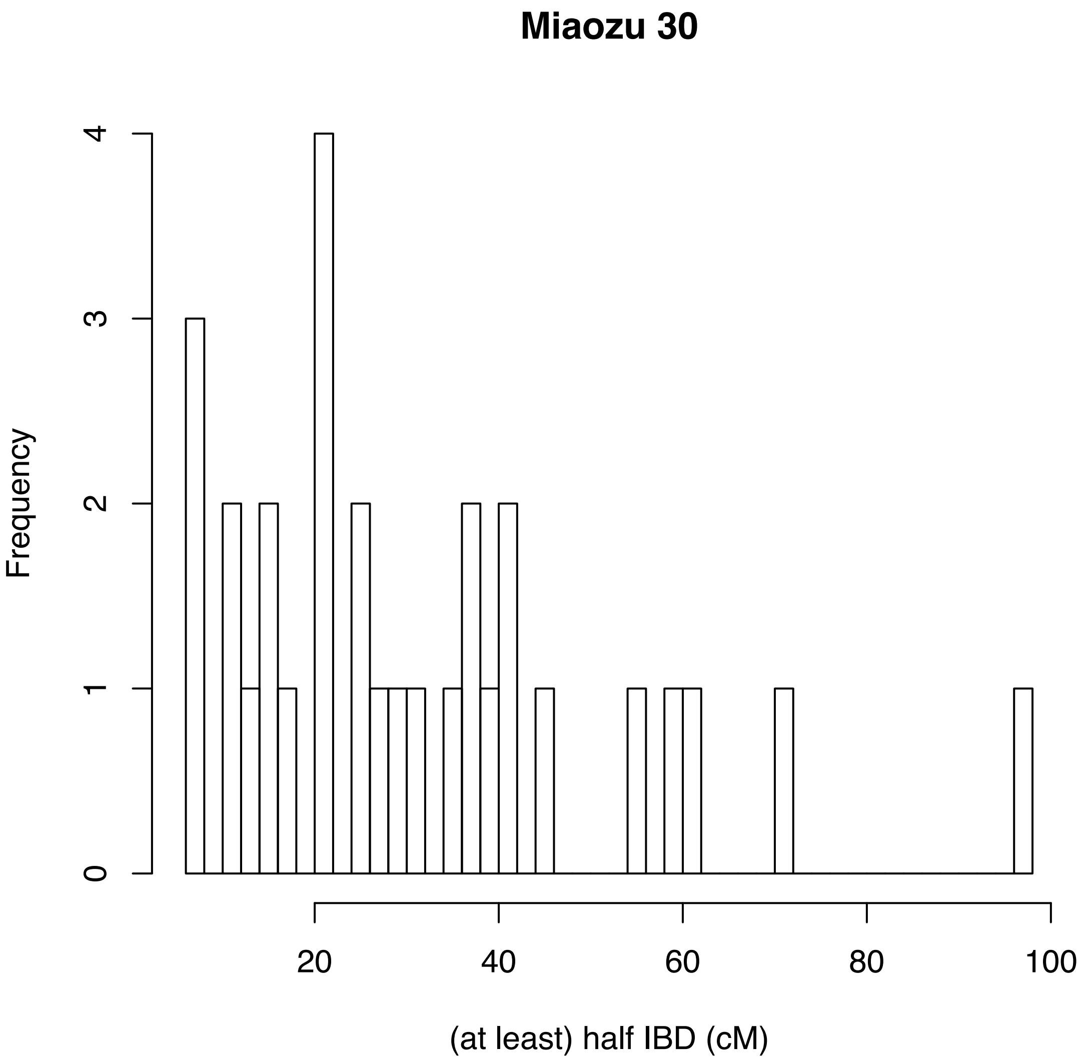

Miaozu 30
Frequency
0
1
2
3
4
20
40
60
80
100
(at least) half IBD (cM)

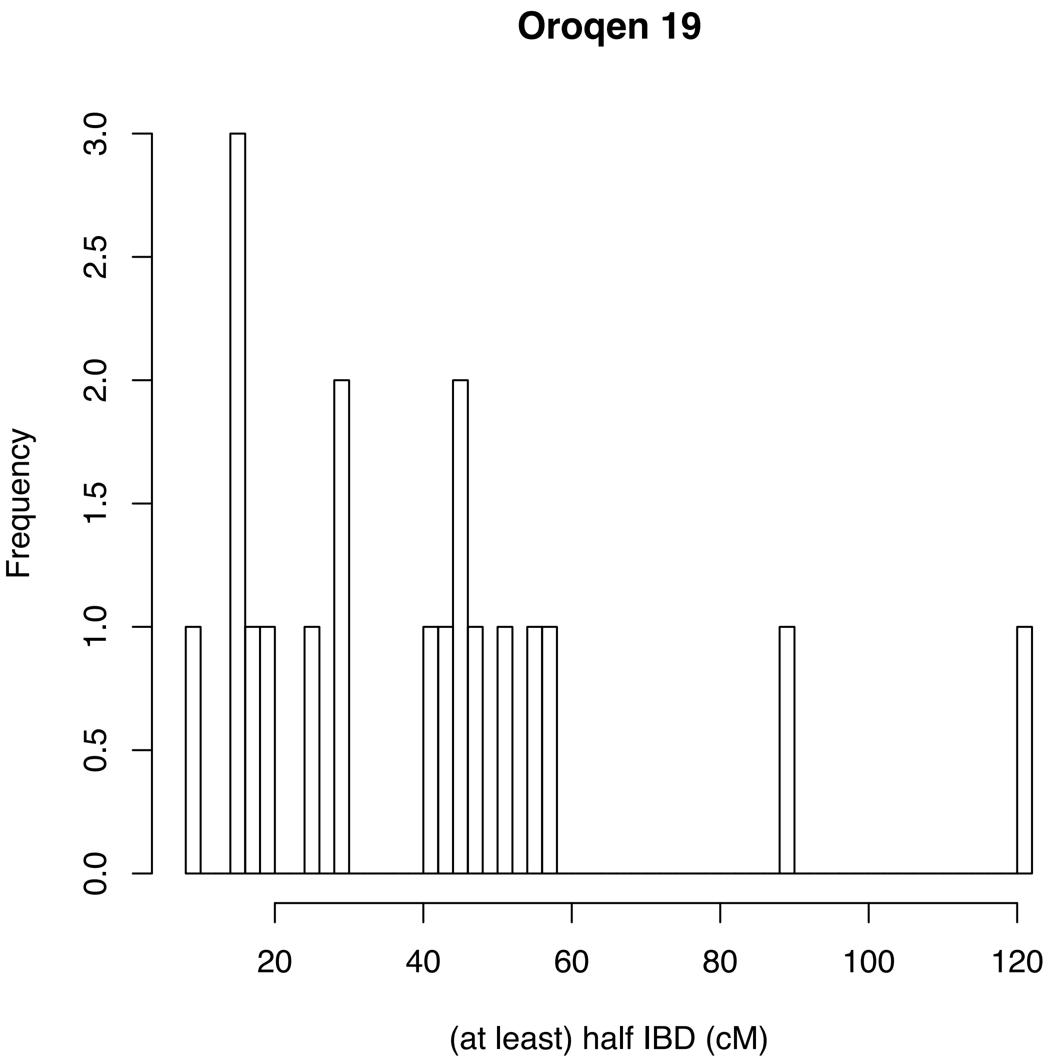

Oroqen 19
Frequency
0.0
0.5
1.0
1.5
2.0
2.5
3.0
20
40
60
80
100
120
(at least) half IBD (cM)

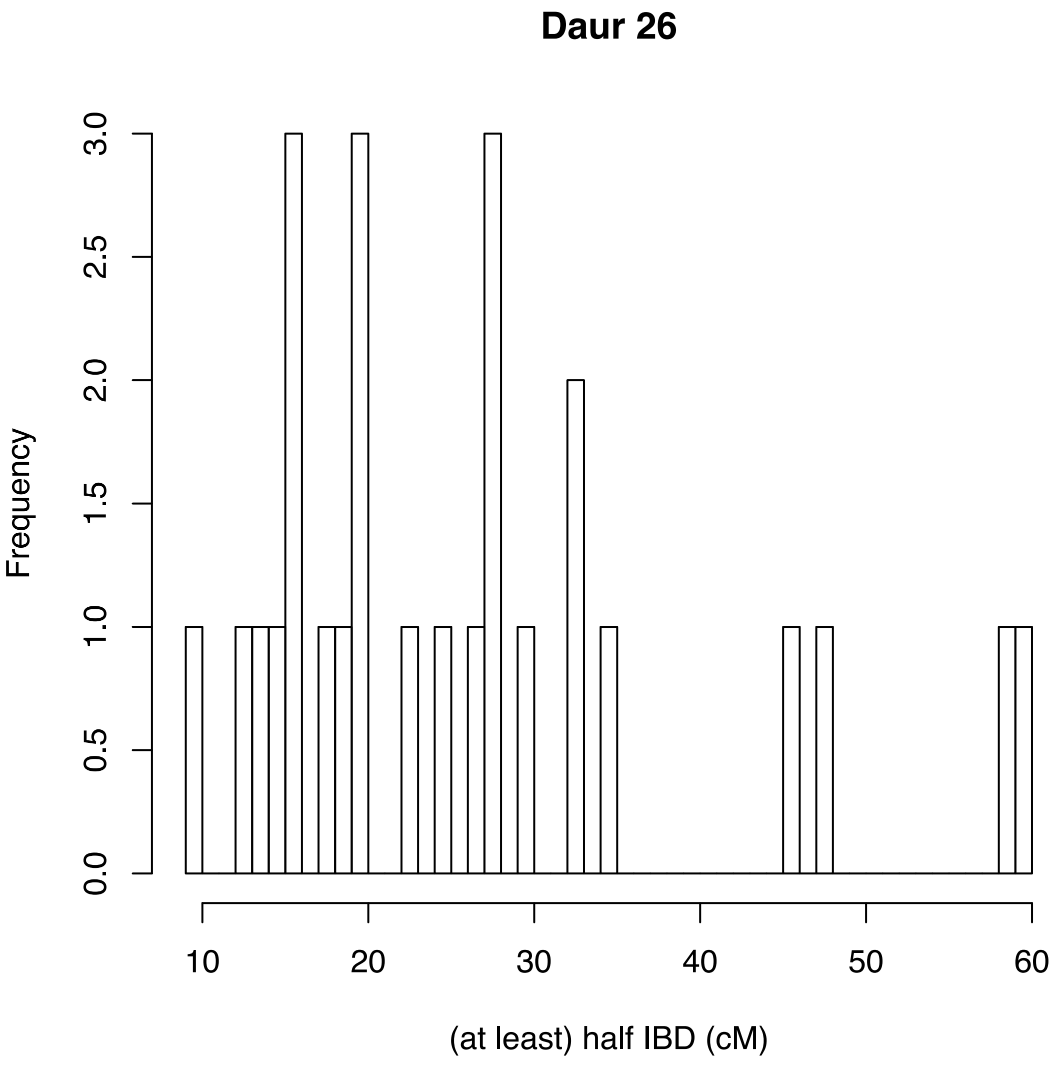
Daur 26
Frequency
0.0
0.5
1.0
1.5
2.0
2.5
3.0
10
20
30
40
50
60
(at least) half IBD (cM)

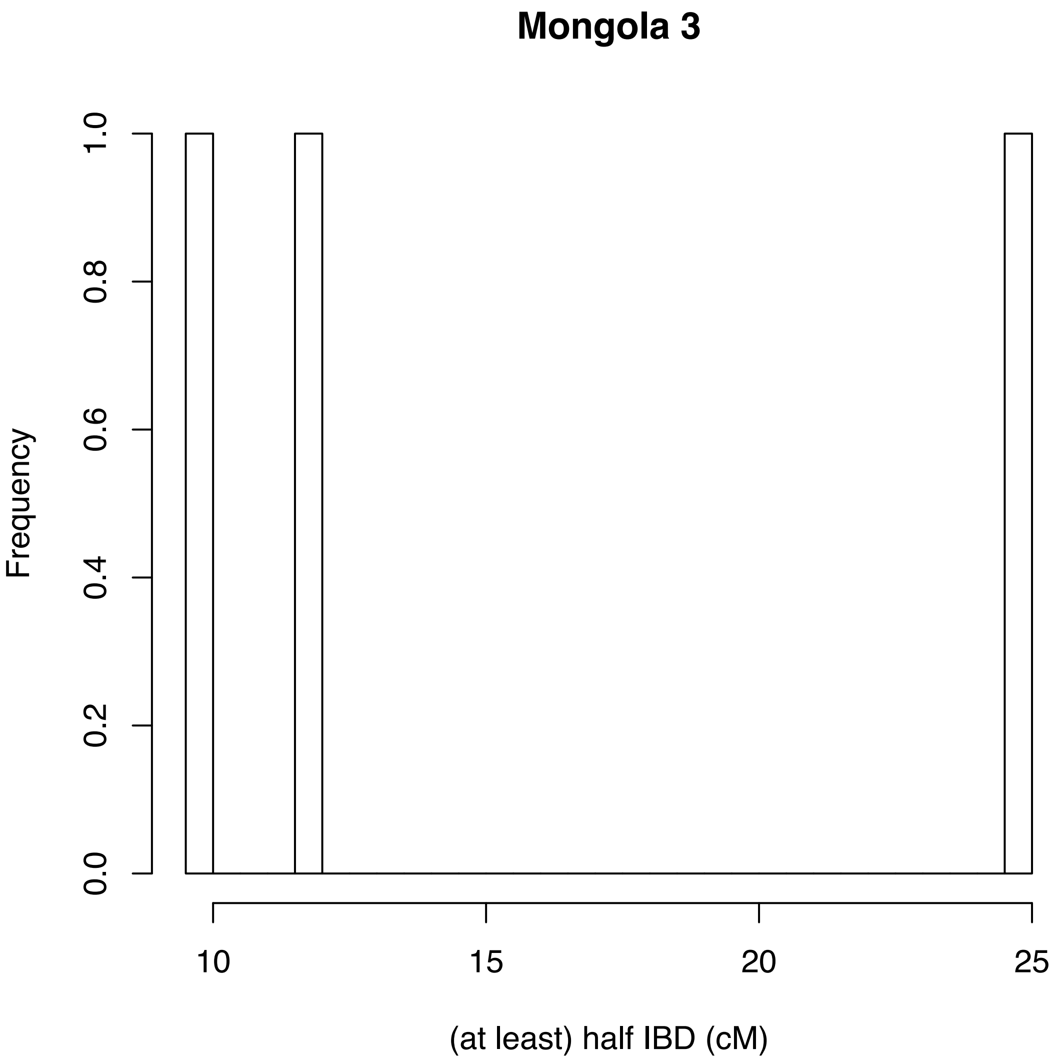
Mongola 3
Frequency
0.0
0.2
0.4
0.6
0.8
1.0
10
15
20
25
(at least) half IBD (cM)

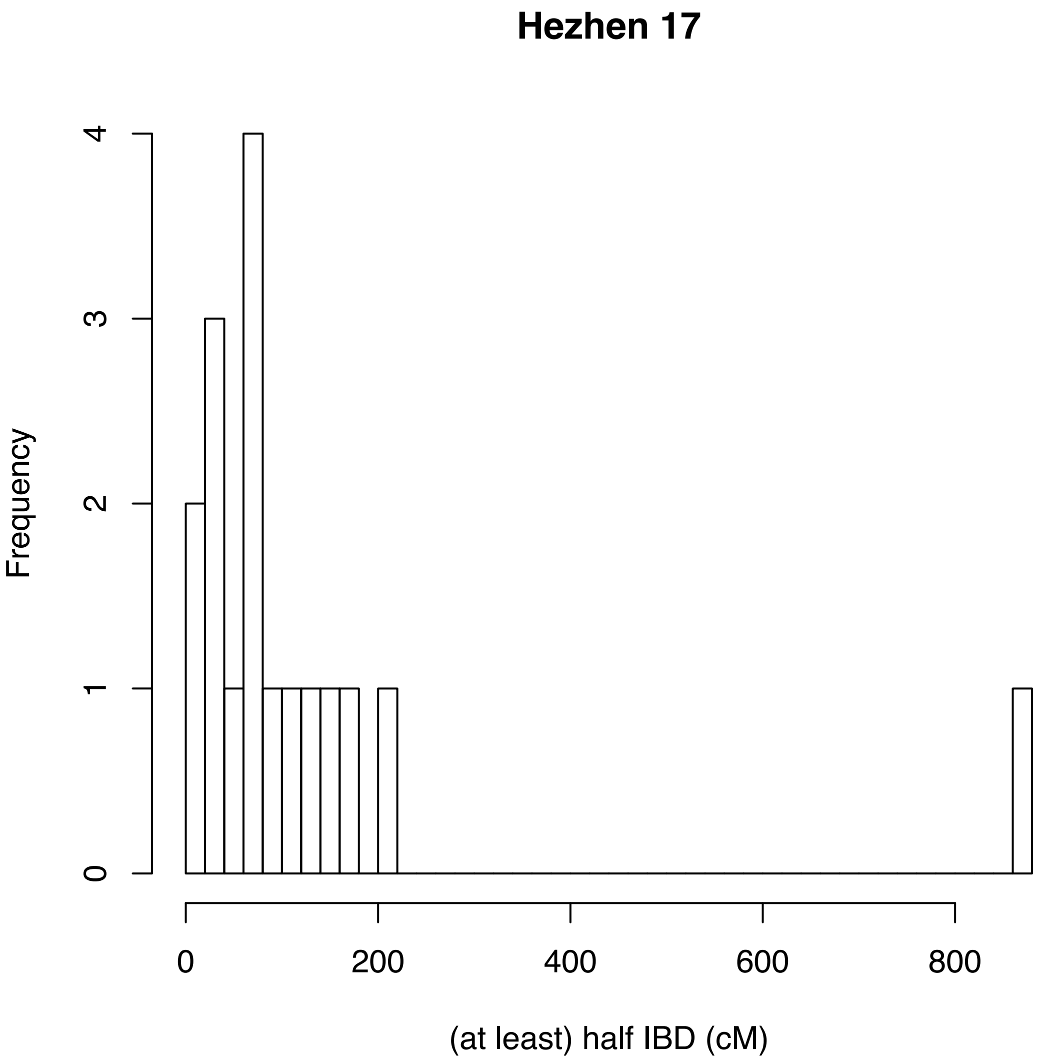

Hezhen 17
Frequency
0
1
2
3
4
0
200
400
600
800
(at least) half IBD (cM)

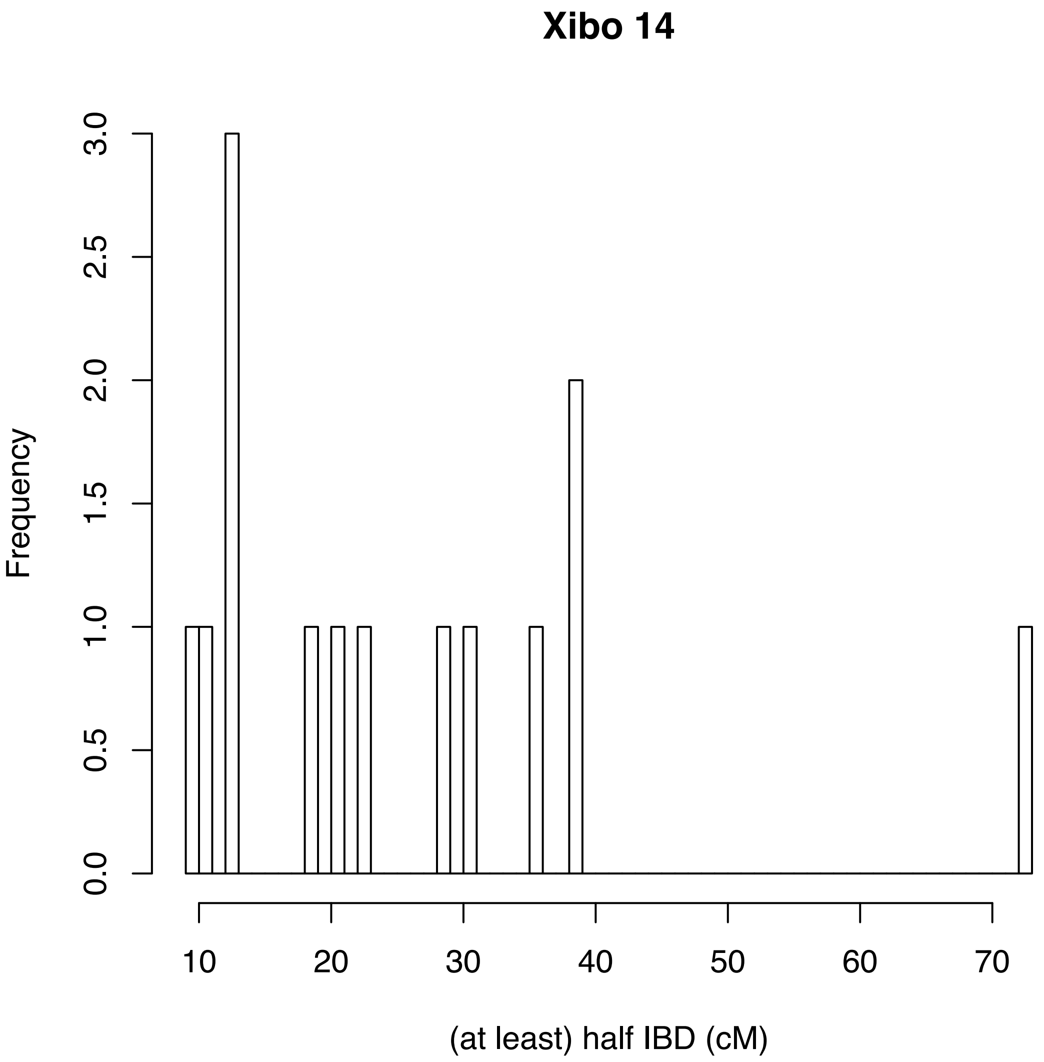

Xibo 14
Frequency
0.0
0.5
1.0
1.5
2.0
2.5
3.0
10
20
30
40
50
60
70
(at least) half IBD (cM)

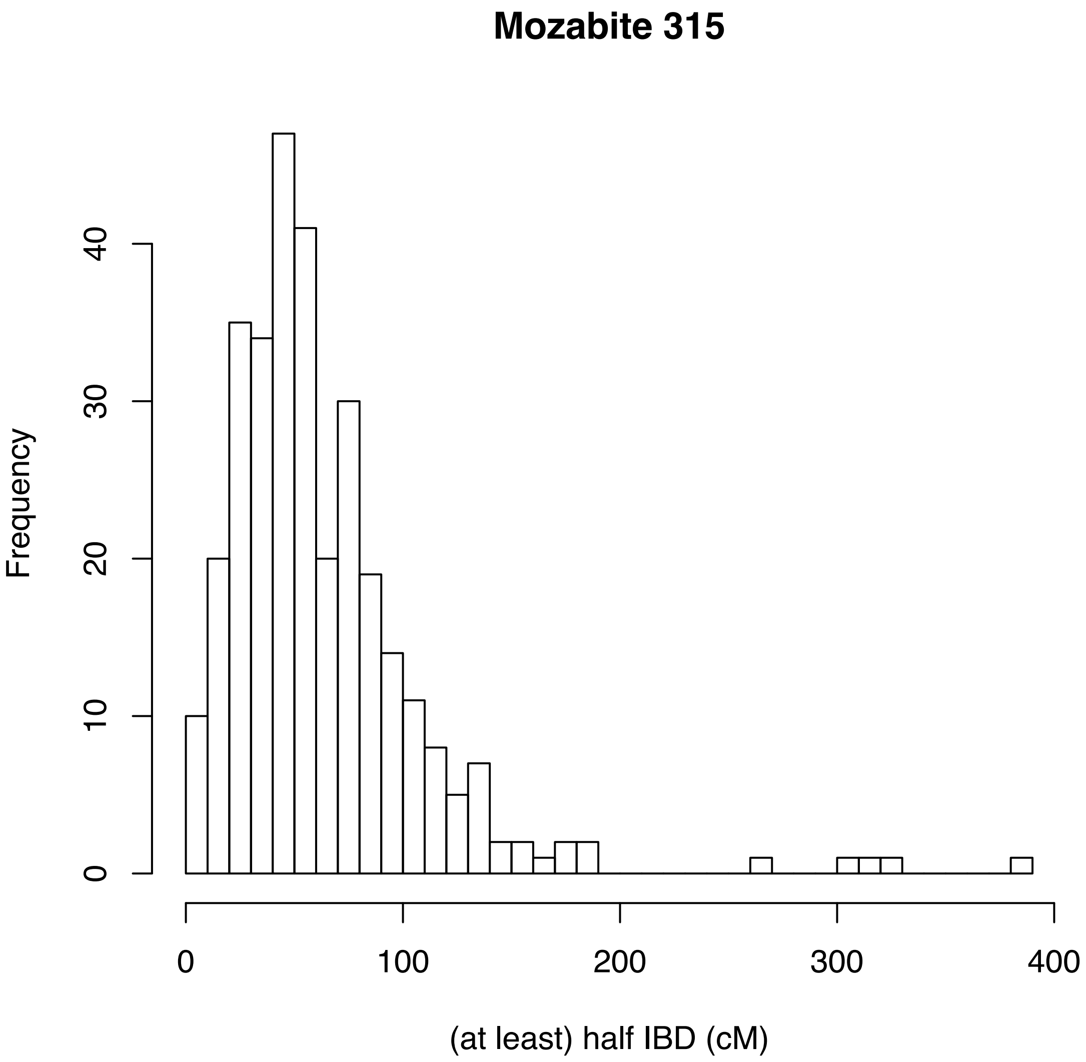

Mozabite 315
Frequency
0
10
20
30
40
0
100
200
300
400
(at least) half IBD (cM)

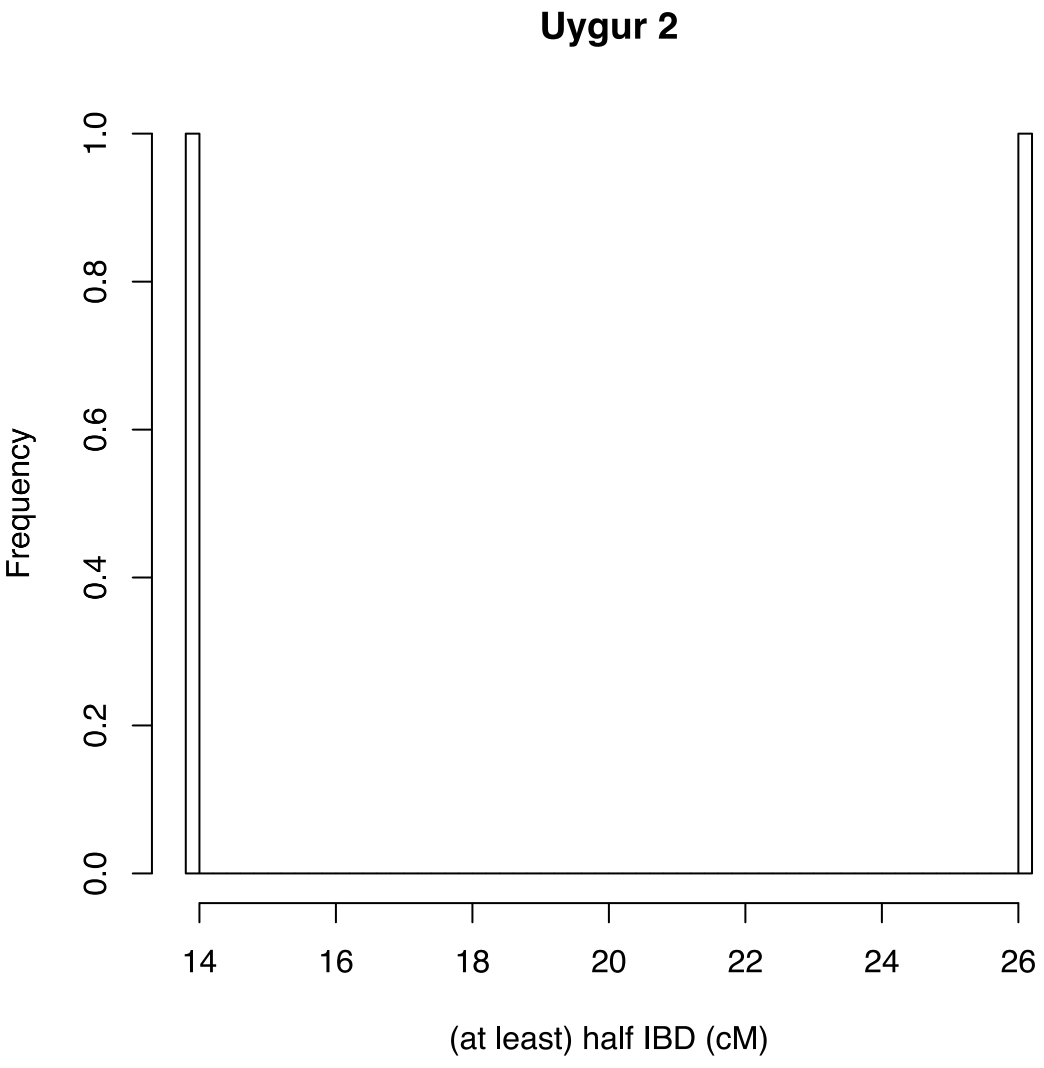

Uygur 2
Frequency
0.0
0.2
0.4
0.6
0.8
1.0
14
16
18
20
22
24
26
(at least) half IBD (cM)

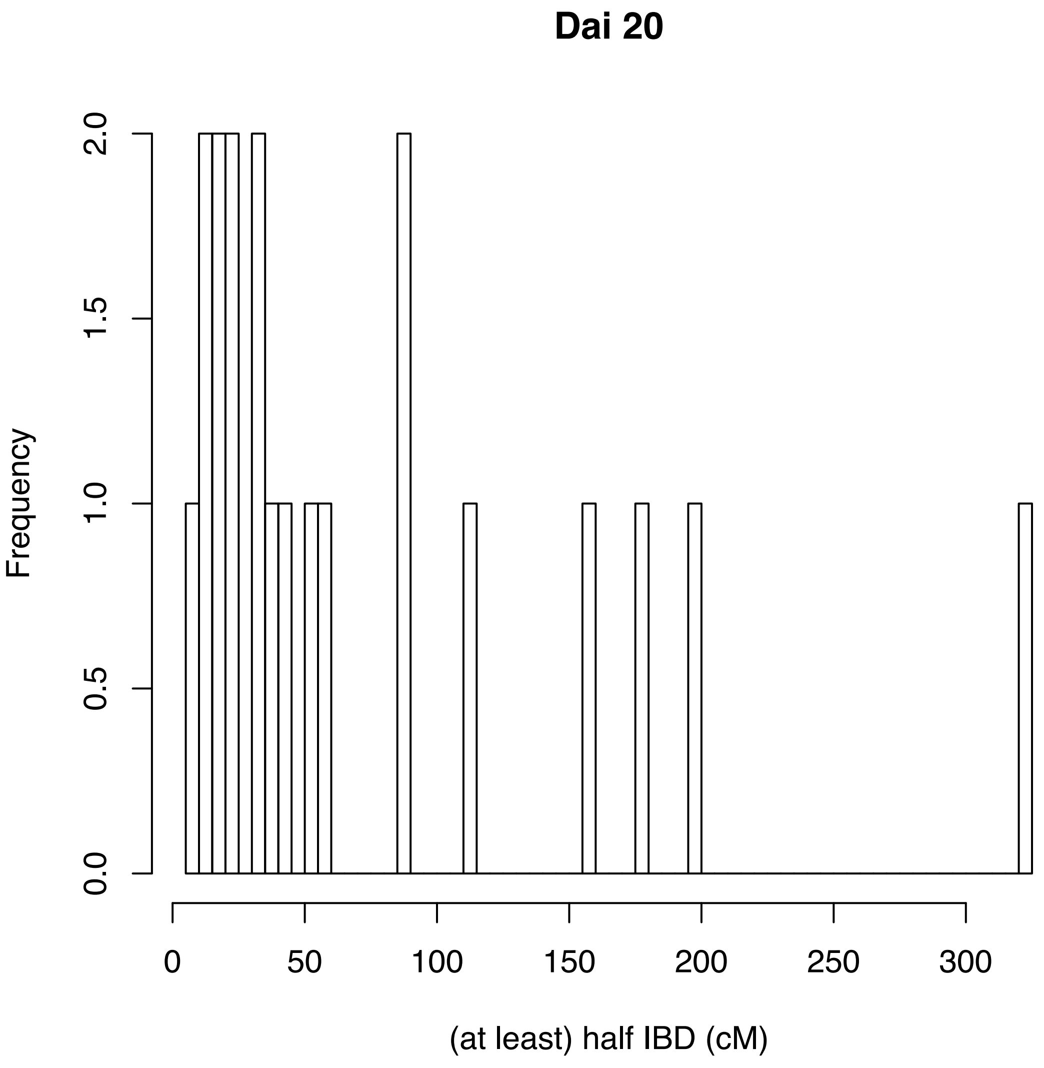

Dai 20
Frequency
0.0
0.5
1.0
1.5
2.0
0
50
100
150
200
250
300
(at least) half IBD (cM)

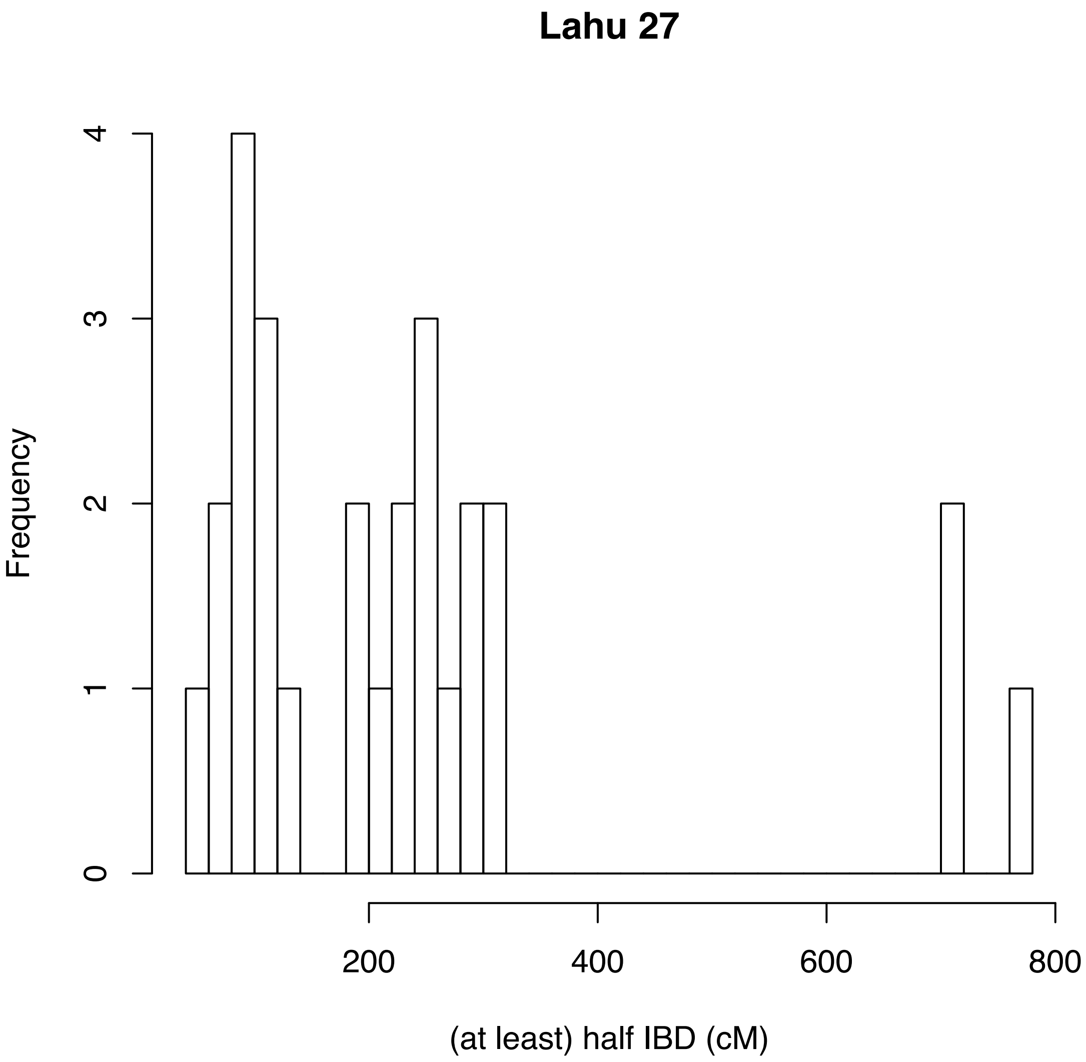

Lahu 27
Frequency
0
1
2
3
4
200
400
600
800
(at least) half IBD (cM)

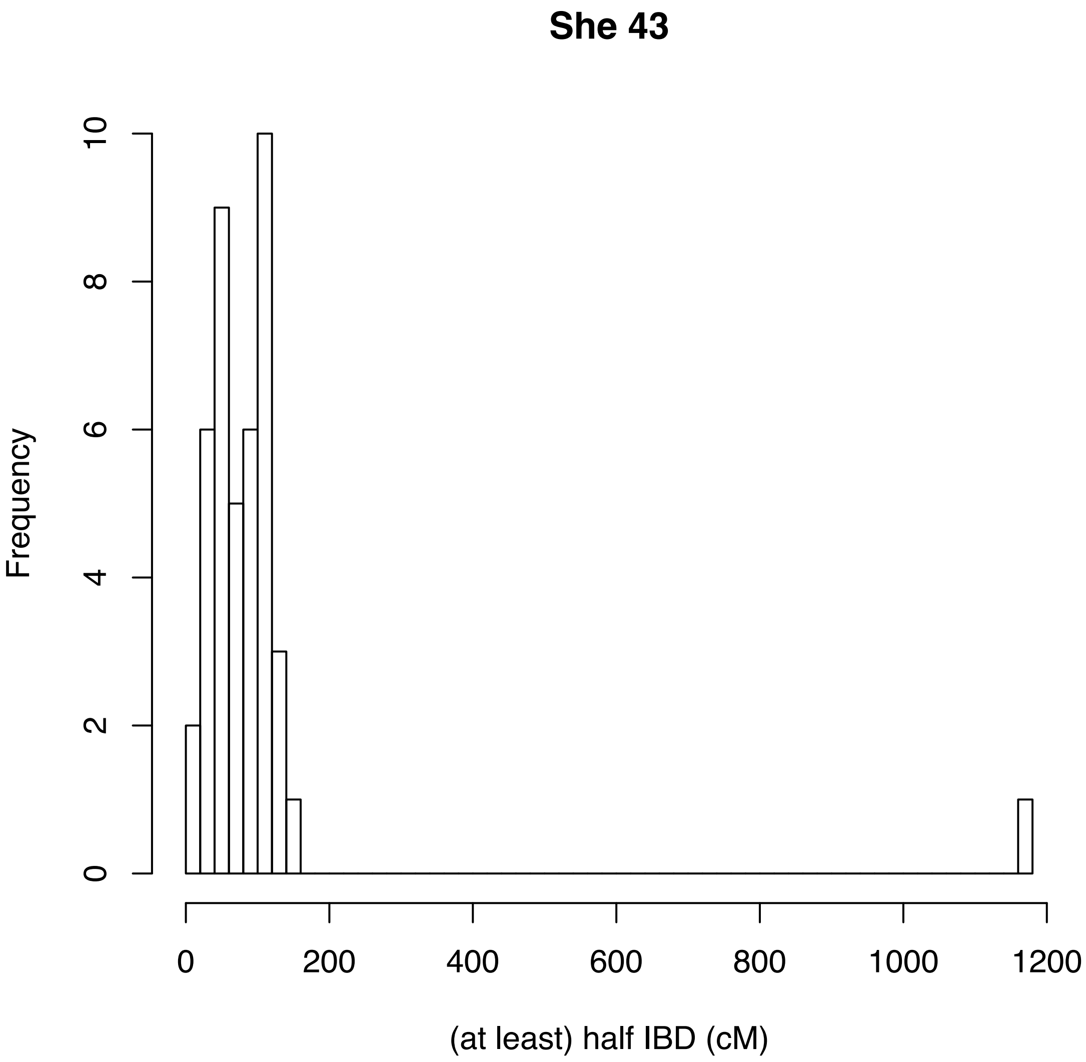

She 43
Frequency
0
2
4
6
8
10
0
200
400
600
800
1000
1200
(at least) half IBD (cM)

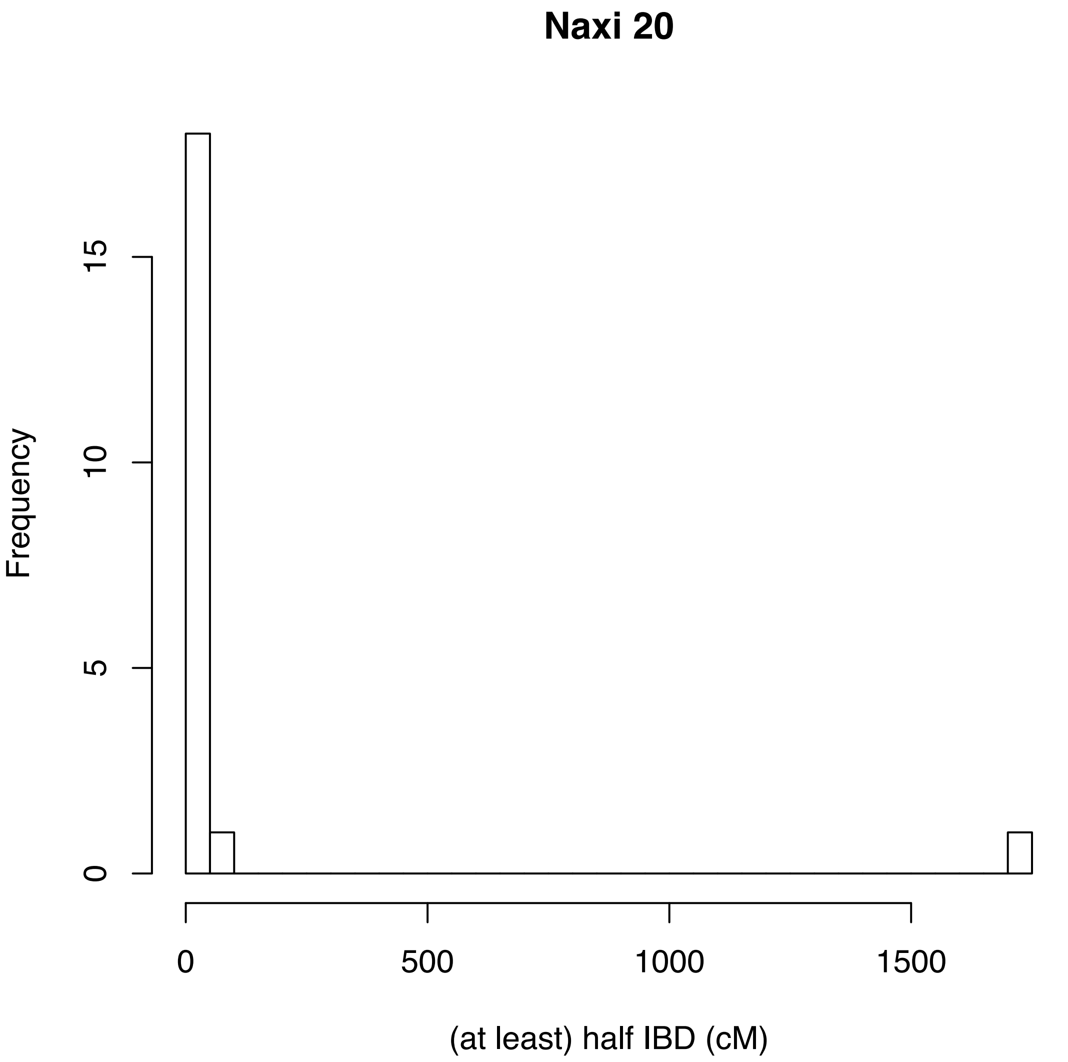
Naxi 20
Frequency
0
5
10
15
0
500
1000
1500
(at least) half IBD (cM)

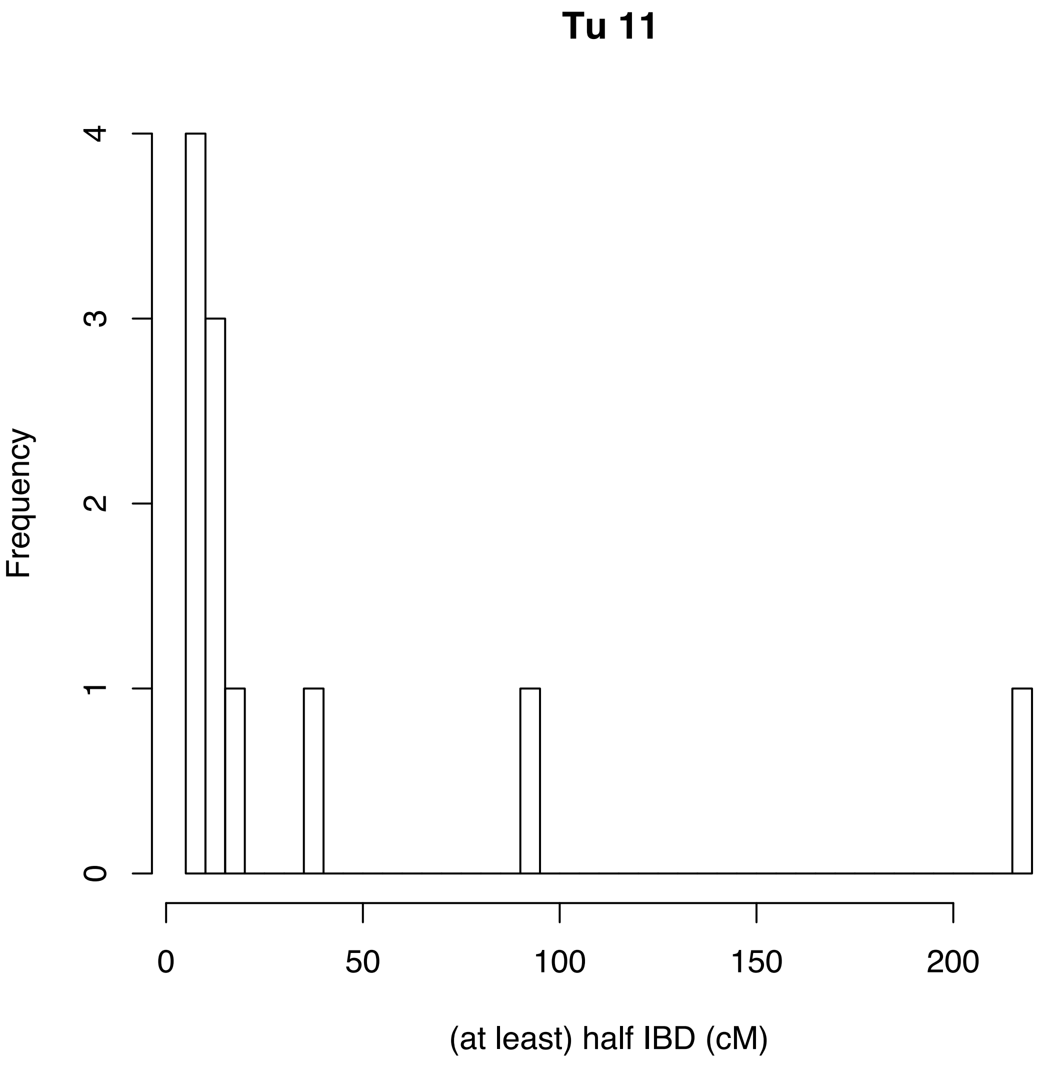

Tu 11
Frequency
0
1
2
3
4
0
50
100
150
200
(at least) half IBD (cM)

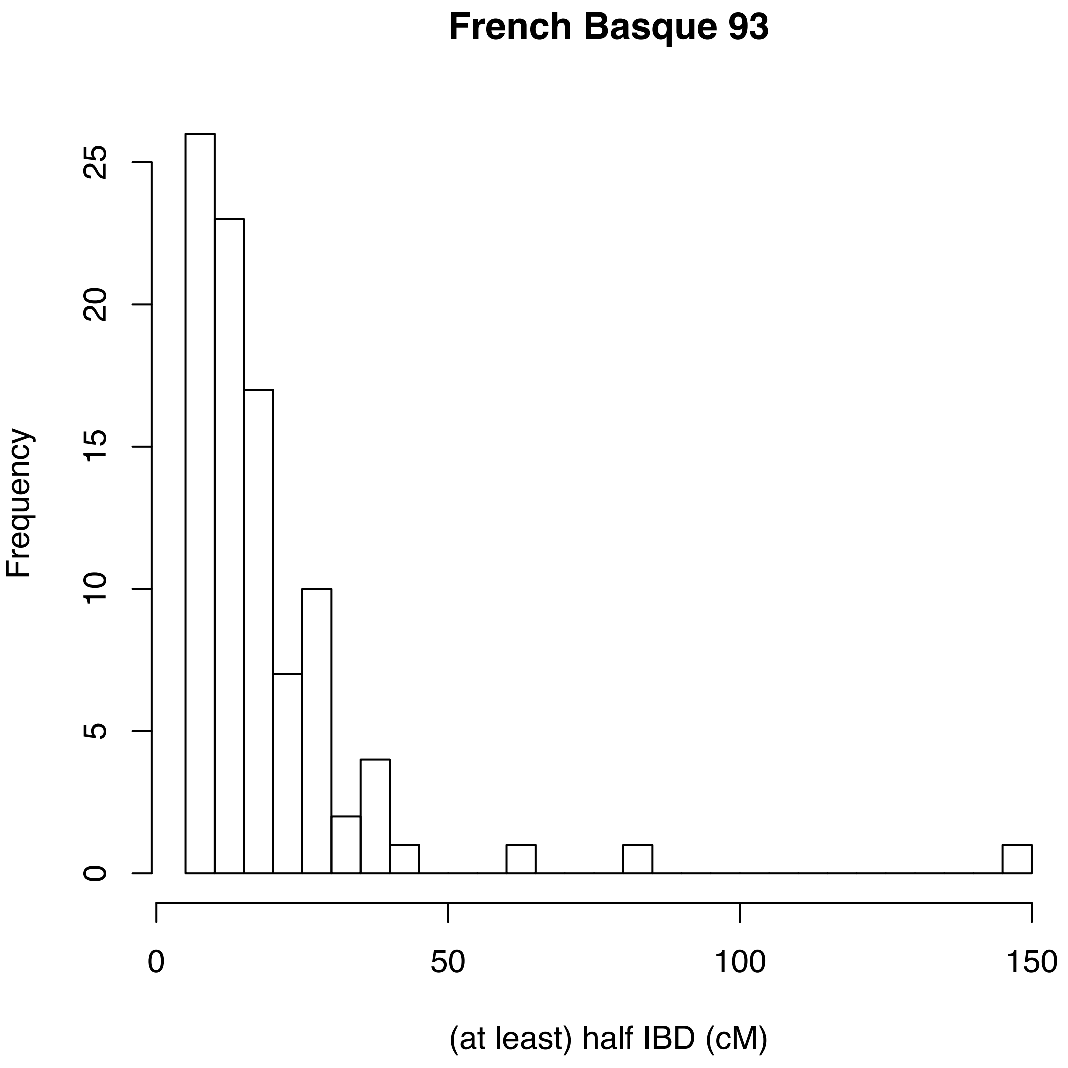
French Basque 93
Frequency
0
5
10
15
20
25
0
50
100
150
(at least) half IBD (cM)

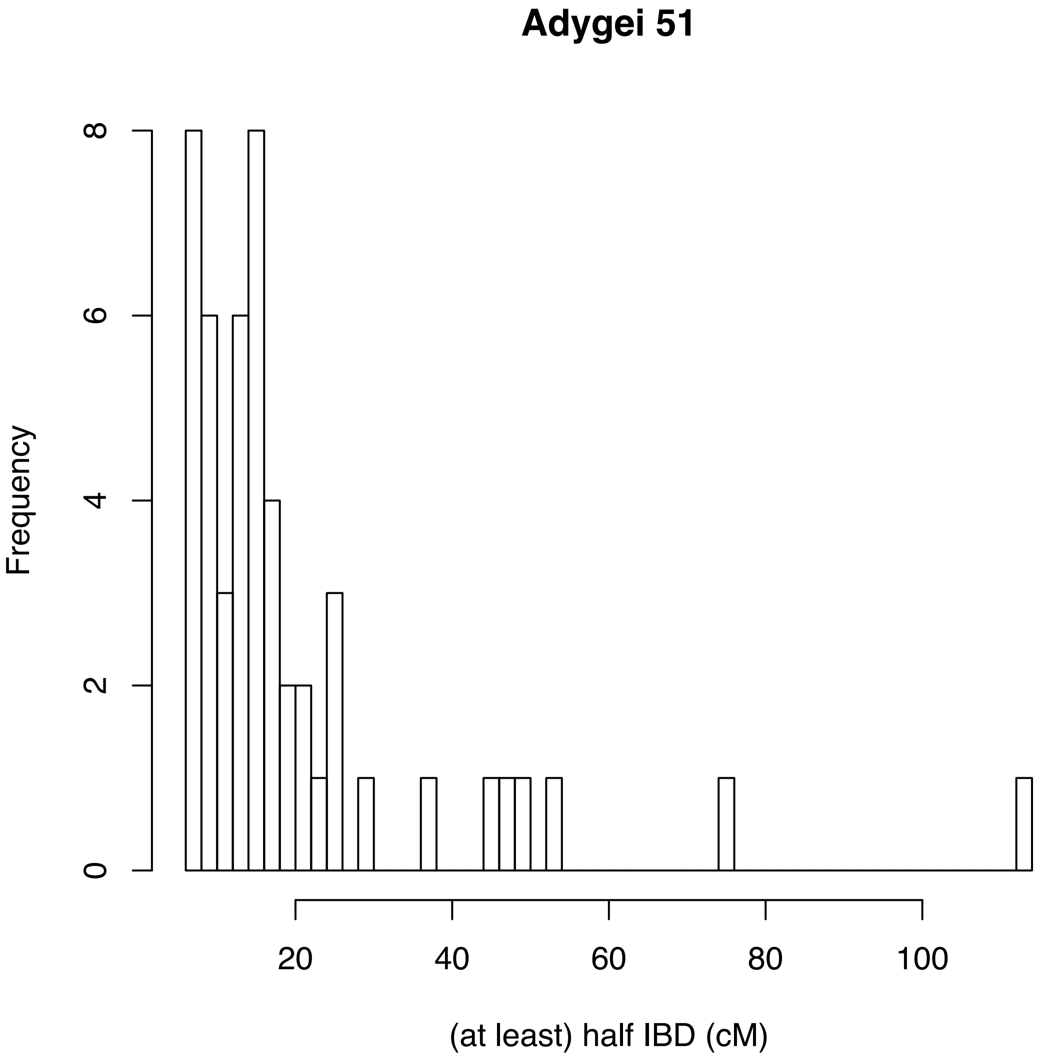

Adygei 51
Frequency
0
2
4
6
8
20
40
60
80
100
(at least) half IBD (cM)

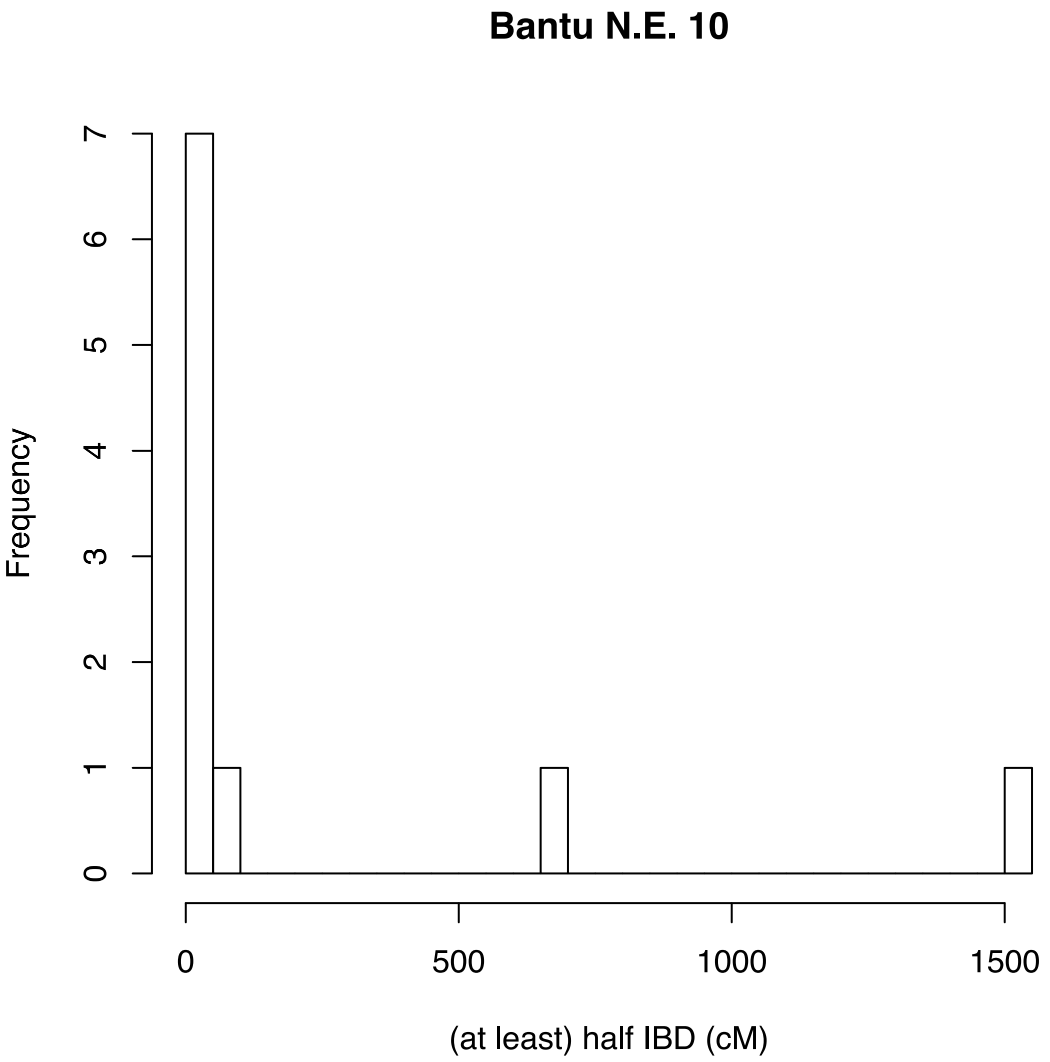
Bantu N.E. 10
Frequency
0
1
2
3
4
5
6
7
0
500
1000
1500
(at least) half IBD (cM)
