# Supplementary figures and images for: Cryptic Distant Relatives Are Common in Both Isolated and Cosmopolitan Genetic Samples
Source: PLoS One. 2012 Apr 3;7(4):e34267. doi: 10.1371/journal.pone.0034267 (PMC3317976; doi:10.1371/journal.pone.0034267)

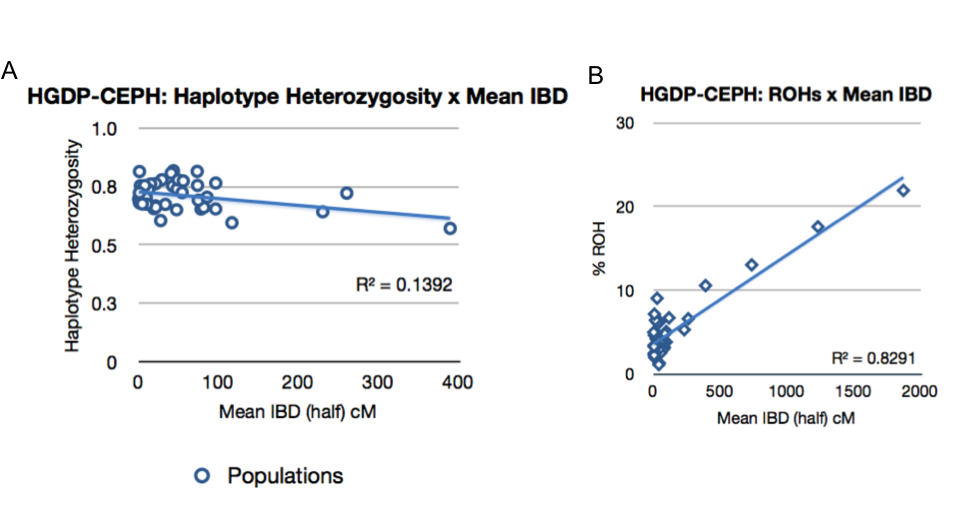

Supplement: Figure S2 — Comparison and correlation of HGDP-CEPH population statistics. a) We compare the mean haplotype heterozygosity statistic obtained from Li et al. [23] with the mean IBDhalf for each of 46 populations from HGDP-CEPH. Native American populations (Karitiana, Surui, Pima) were removed from the analysis due to their extreme levels of IBDhalf, indicating many close relatives within each sample. b) The length of runs of homozygosity (ROHs) for each individual were calculated following the same procedure as Nalls et al. [26]. ROHs were averaged for each population and presented as percent of the genome. We contrast the percent of the genome with ROHs to mean IBDhalf for each population. The correlation between is driven primarily by populations with very high IBDhalf, such as Native Americans. (TIFF) [file pone.0034267.s002.tif]
