# Supplementary material for: Cryptic Distant Relatives Are Common in Both Isolated and Cosmopolitan Genetic Samples
Source: PLoS One. 2012 Apr 3;7(4):e34267. doi: 10.1371/journal.pone.0034267 (PMC3317976; doi:10.1371/journal.pone.0034267)
Supplement: Table S1 — IBD and ROH statistics for HGDP-CEPH and 23andMe population samples. (DOC) [file pone.0034267.s003.doc]

**Table S1:**

*IBD and ROH* statistics for HGDP-CEPH and 23andMe population samples

| **Population (Ethnicity or Country)** | **Sample Size** | **Mean IBD_half_** | **F_IBD_** | **% ROH** | **mean ROH length (cM)** | **Source** |
| --- | --- | --- | --- | --- | --- | --- |
| Surui | 8 | 1870.5 | 1.00 | 22 | 4.08 | CEPH/HGDP |
| Karitiana | 14 | 1229.5 | 0.88 | 18 | 3.13 | CEPH/HGDP |
| Pima | 14 | 734.1 | 1.00 | 13 | 2.90 | CEPH/HGDP |
| Colombian | 7 | 389.4 | 1.00 | 11 | 2.34 | CEPH/HGDP |
| Kalash | 23 | 260.0 | 1.00 | 7 | 2.89 | CEPH/HGDP |
| Lahu | 8 | 230.0 | 0.96 | 5 | 2.35 | CEPH/HGDP |
| Melanesian | 10 | 116.5 | 0.96 | 7 | 1.83 | CEPH/HGDP |
| Druze | 42 | 96.3 | 0.73 | 5 | 3.38 | CEPH/HGDP |
| She | 10 | 96.2 | 0.96 | 4 | 2.15 | CEPH/HGDP |
| Yakut | 25 | 85.4 | 0.92 | 5 | 2.30 | CEPH/HGDP |
| Naxi | 8 | 81.3 | 0.71 | 3 | 1.86 | CEPH/HGDP |
| Hezhen | 8 | 77.9 | 0.61 | 4 | 2.25 | CEPH/HGDP |
| San | 5 | 74.5 | 1.00 | 3 | 2.30 | CEPH/HGDP |
| Mbuti Pygmies | 13 | 73.5 | 0.94 | 3 | 2.35 | CEPH/HGDP |
| Biaka Pygmies | 21 | 73.2 | 0.96 | 3 | 2.71 | CEPH/HGDP |
| Palestinian | 46 | 55.6 | 0.62 | 4 | 3.09 | CEPH/HGDP |
| Orcadian | 15 | 54.3 | 0.68 | 3 | 2.40 | CEPH/HGDP |
| Mozabite | 29 | 49.3 | 0.78 | 4 | 2.79 | CEPH/HGDP |
| Hazara | 22 | 48.0 | 0.52 | 3 | 2.63 | CEPH/HGDP |
| Maya | 21 | 47.3 | 0.47 | 6 | 1.93 | CEPH/HGDP |
| Bantu (N.E.) | 11 | 43.4 | 0.18 | 1 | 2.38 | CEPH/HGDP |
| Burusho | 25 | 42.4 | 0.86 | 3 | 2.78 | CEPH/HGDP |
| Mandenka | 22 | 40.6 | 0.70 | 1 | 2.17 | CEPH/HGDP |
| Dai | 10 | 33.3 | 0.44 | 3 | 2.07 | CEPH/HGDP |
| Bedouin | 46 | 29.0 | 0.31 | 6 | 3.63 | CEPH/HGDP |
| Papuan | 17 | 27.5 | 0.26 | 9 | 2.12 | CEPH/HGDP |
| Ashkenazi ^a^ | 845 | 23.0 | 0.85 | 2 | 2.32 | 23andMe |
| Oroqen | 9 | 21.3 | 0.53 | 4 | 2.06 | CEPH/HGDP |
| Brahui | 25 | 21.2 | 0.65 | 5 | 3.32 | CEPH/HGDP |
| Miaozu | 10 | 20.9 | 0.67 | 4 | 2.18 | CEPH/HGDP |
| Ashkenazi_3GP ^a^ | 178 | 20.1 | 0.80 | 2 | 2.35 | 23andMe |
| Daur | 9 | 19.0 | 0.72 | 3 | 1.93 | CEPH/HGDP |
| Makrani | 25 | 14.9 | 0.26 | 6 | 3.77 | CEPH/HGDP |
| Iceland | 10 | 12.8 | 0.56 | 2 | 2.09 | 23andMe |
| Sardinian | 28 | 12.4 | 0.38 | 3 | 2.35 | CEPH/HGDP |
| Dominican Republic | 26 | 10.6 | 0.45 | 2 | 2.92 | 23andMe |
| Xibo | 9 | 10.1 | 0.39 | 2 | 1.78 | CEPH/HGDP |
| Finland | 149 | 10.0 | 0.53 | 3 | 2.16 | 23andMe |
| Tu | 10 | 9.6 | 0.24 | 3 | 1.92 | CEPH/HGDP |
| Tuscan | 8 | 9.3 | 0.43 | 2 | 2.28 | CEPH/HGDP |
| Ashkenazi_2GP ^a^ | 499 | 8.2 | 0.46 | 2 | 2.35 | 23andMe |
| South Africa | 19 | 7.8 | 0.32 | 2 | 1.97 | 23andMe |
| Colombia | 42 | 7.7 | 0.25 | 2 | 2.40 | 23andMe |
| Adygei | 17 | 7.6 | 0.38 | 2 | 2.31 | CEPH/HGDP |
| Sindhi | 24 | 7.6 | 0.06 | 5 | 3.25 | CEPH/HGDP |
| Balochi | 24 | 7.5 | 0.21 | 7 | 3.89 | CEPH/HGDP |
| French Basque | 24 | 6.4 | 0.34 | 3 | 2.31 | CEPH/HGDP |
| Russian | 25 | 5.6 | 0.32 | 2 | 2.11 | CEPH/HGDP |
| Yizu | 10 | 4.5 | 0.22 | 3 | 1.89 | CEPH/HGDP |
| Lebanon | 27 | 4.2 | 0.24 | 3 | 2.91 | 23andMe |
| Palestine | 14 | 4.0 | 0.10 | 5 | 3.45 | 23andMe |
| Morocco | 13 | 3.9 | 0.15 | 2 | 2.55 | 23andMe |
| Ukraine | 67 | 3.6 | 0.15 | 2 | 2.27 | 23andMe |
| Iraq | 18 | 3.3 | 0.07 | 3 | 2.77 | 23andMe |
| Egypt | 18 | 2.9 | 0.05 | 2 | 2.47 | 23andMe |
| Belarus | 16 | 2.8 | 0.11 | 2 | 2.05 | 23andMe |
| Cyprus | 11 | 2.6 | 0.20 | 2 | 2.52 | 23andMe |
| Slovenia | 11 | 2.5 | 0.17 | 2 | 2.10 | 23andMe |
| Ashkenazi_1GP ^a^ | 473 | 2.5 | 0.15 | 2 | 2.31 | 23andMe |
| Syria | 14 | 2.4 | 0.10 | 2 | 2.40 | 23andMe |
| Bosnia and Herzegovina | 16 | 2.1 | 0.18 | 2 | 2.30 | 23andMe |
| Pathan | 22 | 2.1 | 0.13 | 5 | 3.22 | CEPH/HGDP |
| Chile | 11 | 2.1 | 0.12 | 2 | 1.95 | 23andMe |
| Estonia | 18 | 1.7 | 0.15 | 2 | 2.18 | 23andMe |
| North Italian | 12 | 1.7 | 0.11 | 2 | 2.39 | CEPH/HGDP |
| Serbia | 11 | 1.7 | 0.12 | 2 | 2.57 | 23andMe |
| Cuba | 33 | 1.6 | 0.10 | 2 | 2.26 | 23andMe |
| Norway | 143 | 1.5 | 0.10 | 2 | 2.23 | 23andMe |
| Sri Lanka | 15 | 1.5 | 0.08 | 3 | 2.52 | 23andMe |
| Mexico | 148 | 1.4 | 0.09 | 2 | 2.20 | 23andMe |
| Peru | 15 | 1.4 | 0.08 | 3 | 1.90 | 23andMe |
| Lithuania | 31 | 1.4 | 0.09 | 2 | 2.06 | 23andMe |
| Ecuador | 11 | 1.4 | 0.08 | 3 | 2.24 | 23andMe |
| Jamaica | 22 | 1.2 | 0.04 | 1 | 2.43 | 23andMe |
| Philippines | 70 | 1.2 | 0.09 | 3 | 1.94 | 23andMe |
| Macedonia | 11 | 1.1 | 0.09 | 2 | 2.09 | 23andMe |
| Pakistan | 26 | 1.1 | 0.07 | 4 | 3.17 | 23andMe |
| Croatia | 34 | 1.1 | 0.09 | 2 | 2.38 | 23andMe |
| Mongolian | 10 | 1.0 | 0.07 | 2 | 1.77 | CEPH/HGDP |
| Yoruba | 21 | 1.0 | 0.06 | 1 | 2.22 | CEPH/HGDP |
| Hungary | 64 | 1.0 | 0.04 | 2 | 2.20 | 23andMe |
| Uygur | 10 | 0.9 | 0.04 | 2 | 2.32 | CEPH/HGDP |
| Sweden | 108 | 0.8 | 0.06 | 2 | 2.23 | 23andMe |
| Russia | 161 | 0.8 | 0.04 | 2 | 2.32 | 23andMe |
| Ireland | 180 | 0.7 | 0.06 | 2 | 2.15 | 23andMe |
| Portugal | 85 | 0.7 | 0.05 | 2 | 2.37 | 23andMe |
| Slovakia | 28 | 0.7 | 0.05 | 2 | 2.31 | 23andMe |
| Greece | 92 | 0.7 | 0.05 | 2 | 2.33 | 23andMe |
| Denmark | 88 | 0.6 | 0.05 | 2 | 2.30 | 23andMe |
| Bulgaria | 48 | 0.6 | 0.05 | 2 | 2.27 | 23andMe |
| Switzerland | 64 | 0.6 | 0.05 | 2 | 2.23 | 23andMe |
| Canada | 373 | 0.6 | 0.04 | 2 | 2.18 | 23andMe |
| Romania | 89 | 0.6 | 0.05 | 2 | 2.21 | 23andMe |
| Iran | 97 | 0.6 | 0.02 | 2 | 2.41 | 23andMe |
| Brazil | 75 | 0.5 | 0.03 | 2 | 2.45 | 23andMe |
| Vietnam | 30 | 0.5 | 0.05 | 3 | 1.93 | 23andMe |
| Haiti | 12 | 0.5 | 0.01 | 1 | 2.71 | 23andMe |
| Poland | 174 | 0.5 | 0.03 | 2 | 2.23 | 23andMe |
| Taiwan | 51 | 0.5 | 0.04 | 3 | 1.95 | 23andMe |
| Cambodian | 10 | 0.4 | 0.04 | 2 | 1.87 | CEPH/HGDP |
| Netherlands | 150 | 0.4 | 0.03 | 2 | 2.21 | 23andMe |
| Austria | 33 | 0.4 | 0.02 | 2 | 2.13 | 23andMe |
| Belgium | 55 | 0.4 | 0.03 | 2 | 2.35 | 23andMe |
| Han | 44 | 0.3 | 0.01 | 3 | 1.84 | CEPH/HGDP |
| South Korea | 60 | 0.3 | 0.02 | 3 | 2.10 | 23andMe |
| Japan | 78 | 0.2 | 0.02 | 3 | 1.99 | 23andMe |
| New Zealand | 26 | 0.2 | 0.02 | 2 | 2.29 | 23andMe |
| India | 363 | 0.2 | 0.01 | 2 | 2.33 | 23andMe |
| Japanese | 28 | 0.2 | 0.02 | 3 | 1.85 | CEPH/HGDP |
| Spain | 159 | 0.2 | 0.02 | 2 | 2.37 | 23andMe |
| Czech Republic | 29 | 0.2 | 0.02 | 2 | 2.18 | 23andMe |
| Australia | 139 | 0.2 | 0.01 | 2 | 2.32 | 23andMe |
| Germany | 225 | 0.2 | 0.01 | 2 | 2.26 | 23andMe |
| United Kingdom | 541 | 0.1 | 0.01 | 2 | 2.22 | 23andMe |
| Armenia | 10 | 0.1 | 0.02 | 2 | 2.90 | 23andMe |
| Turkey | 82 | 0.1 | 0.01 | 2 | 2.50 | 23andMe |
| United States of America | 9804 | 0.1 | 0.01 | 2 | 2.22 | 23andMe |
| China | 277 | 0.1 | 0.01 | 3 | 2.02 | 23andMe |
| Italy | 386 | 0.1 | 0.01 | 2 | 2.36 | 23andMe |
| France | 140 | 0.1 | 0.00 | 2 | 2.18 | 23andMe |
| Argentina | 16 | 0.0 | 0.00 | 2 | 2.32 | 23andMe |

^a^ Populations included from the 23andMe database were based on self-reporting by customers of 4 grandparents from the same country. Four Ashkenazi samples are included; each sample reflects between 1 to 4 self-reported Ashkenazi grandparents.
