# Supplementary material for: Cryptic Distant Relatives Are Common in Both Isolated and Cosmopolitan Genetic Samples
Source: PLoS One. 2012 Apr 3;7(4):e34267. doi: 10.1371/journal.pone.0034267 (PMC3317976; doi:10.1371/journal.pone.0034267)
Supplement: Table S2 — Detectability of Ashkenazi Relatives. (DOC) [file pone.0034267.s004.doc]

**Table S2:**

**Detectability of Ashkenazi Relatives**

| **N_pop_^1^** | **Probability of Detecting ≥1 relative^2^** | **Expected # of Detectable Relatives^2^** |
| --- | --- | --- |
| 100,000 | >0.999 | 32 |
| 200,000 | >0.999 | 16 |
| 300,000 | >0.999 | 11 |
| 400,000 | >0.999 | 8 |
| 500,000 | 0.998 | 6 |
| 600,000 | 0.995 | 5 |
| 700,000 | 0.990 | 5 |
| 800,000 | 0.982 | 4 |
| 900,000 | 0.972 | 4 |
| 1,000,000 | 0.959 | 3 |

**^1^** Current population size from which Ashkenazi individuals are sampled. Population size can be less than the census size due to population structure (i.e., non-random mating due to geographic or cultural factors).

**^2^** The probability of detecting at least one distant relative (2^nd^-9^th^ degree cousins) in our dataset for a given Ashkenazi individual. We assume a dataset size of 300 individuals and a specific model of pedigree (described in *Supp*. *Methods*). The probability and expected number of relatives includes both *n*th degree cousins and *n*th degree cousins once removed.
